# Ancient plants with ancient fungi: liverworts associate with early-diverging arbuscular mycorrhizal fungi

**DOI:** 10.1098/rspb.2018.1600

**Published:** 2018-10-10

**Authors:** William R. Rimington, Silvia Pressel, Jeffrey G. Duckett, Katie J. Field, David J. Read, Martin I. Bidartondo

**Affiliations:** 1 Department of Life Sciences, Imperial College London, London SW7 2AZ, UK; 2 Life Sciences Department, Algae, Fungi and Plants Division, Natural History Museum, London SW7 5BD, UK; 3 Jodrell Laboratory, Royal Botanic Gardens, Kew, Richmond TW9 3DS, UK; 4 Centre for Plant Sciences, Faculty of Biological Sciences, University of Leeds, Leeds LS2 9JT, UK; 5 Department of Animal and Plant Sciences, University of Sheffield, Sheffield S10 2TN, UK

**Keywords:** ancestral plant–fungus symbiosis, arbuscular mycorrhizas, Glomeromycotina, liverworts, plant terrestrialization

## Abstract

Arbuscular mycorrhizas are widespread in land plants including liverworts, some of the closest living relatives of the first plants to colonize land 500 million years ago (MYA). Previous investigations reported near-exclusive colonization of liverworts by the most recently evolved arbuscular mycorrhizal fungi, the Glomeraceae, indicating a recent acquisition from flowering plants at odds with the widely held notion that arbuscular mycorrhizal-like associations in liverworts represent the ancestral symbiotic condition in land plants. We performed an analysis of symbiotic fungi in 674 globally collected liverworts using molecular phylogenetics and electron microscopy. Here, we show every order of arbuscular mycorrhizal fungi colonizes early-diverging liverworts, with non-Glomeraceae being at least 10 times more common than in flowering plants. Arbuscular mycorrhizal fungi in liverworts and other ancient plant lineages (hornworts, lycopods, and ferns) were delimited into 58 taxa and 36 singletons, of which at least 43 are novel and specific to liverworts. The discovery that early plant lineages are colonized by early-diverging fungi supports the hypothesis that arbuscular mycorrhizas are an ancestral symbiosis for all land plants.

## 1. Background

Arbuscular mycorrhizas (AM) are the most widespread land plant–fungus mutualisms; members of the AM fungal lineage (Glomeromycotina) colonize at least 72% of flowering plant species [1]. In this mutually beneficial partnership the host plant receives nutrients from the fungus, in particular phosphorus, in exchange for photosynthesis-derived carbohydrates and lipids [2]. This relationship is essential to the functioning of present-day ecosystems and likely played a key role in facilitating the transition of plants onto land [3–5]. The identity of the fungi that formed the ancestral symbioses with land plants is a subject of current debate [4]. Here, we carry out molecular phylogenetic and ultrastructural analyses designed to uncover critical early fungal partners of land plants.

All arbuscular mycorrhizal fungi (AMF), with the possible exception of fine root endophytes [6], belong to the subphylum Glomeromycotina of the Mucoromycota [7]. The Glomeraceae are by far the most diverse and well-studied AMF. This family consists of more than double the number of morphospecies of the second largest AMF family (Gigasporaceae) [8] and represents 242 of the 357 AMF ‘virtual taxa’ (species delimited based on DNA sequences rather than morphology) [9]. The Glomeraceae has the largest global distribution of any AMF family [10] and is by far the most commonly detected in studies of flowering plant roots, where it represents more than 90% of the recorded AMF [11]. Despite the global dominance of Glomeraceae over the other (non-Glomeraceae) AMF families, it was the most recent to diverge; fossil evidence and molecular clock analyses place its origin less than 400 million years ago (MYA) [12,13], over 100 MYA after plant terrestrialization [5].

In addition to flowering plants, AMF regularly colonize non-vascular bryophytes (liverworts and hornworts, except mosses). While uncertainties remain regarding their order of divergence, the position of bryophytes at the base of the land plant tree is well established [14]. Early-diverging liverworts (i.e. extant members of the first liverwort groups to diverge) and hornworts can form associations with Glomeromycotina and Mucoromycotina fungi [15–19]. There is no evidence of mycorrhizal-like associations in mosses, likely due to their unique fungal-like multicellular rhizoids removing the need for symbiosis [4,20]. Unlike vascular plants, bryophytes lack roots so technically cannot form mycorrhizas [21]. However, recent carbon-for-nutrient exchange studies, including early-diverging (Haplomitriopsida and Marchantiopsida) and derived liverwort groups (Jungermanniopsida) and three fungal clades (Mucoromycotina, Glomeromycotina, and Ascomycota), all show mutualistic nutrient-for-carbon exchanges indicating these are ‘mycorrhizal-like’ symbioses [19,21–24]. The three earliest-diverging liverwort groups—Haplomitriopsida, Marchantiopsida (complex thalloids), and Pelliidae (simple thalloids I) [25]—form symbioses with members of the Glomeromycotina and/or Mucoromycotina, with the exception of some derived clades which are asymbiotic [20]. The later-diverging Metzgeriidae and Jungermanniidae instead form associations with Basidiomycota or Ascomycota, the most recent fungal lineages, or do not form fungal symbioses [20].

As one of the earliest-diverging lineages of extant mycorrhizal or ‘mycorrhizal-like’ plants, liverworts present us with unique opportunities to unravel the origin and early evolutionary history of plant–fungus symbioses. However, at odds with the widely held notion of an ancient partnership between liverworts and AMF, from the dawn of land plant evolution are molecular identifications of Glomeraceae as the colonists of this early lineage of land plants [15,16,26]. The exclusive or near-exclusive colonization by Glomeraceae (a family that evolved after plant terrestrialization) reported for early-diverging plants suggests that liverwort AM-like associations do not represent the symbiotic status of the first land plants and instead symbiosis is derived, having host-shifted from flowering plants [27]. If this were the case then there are substantial repercussions for current interpretations of fast accumulating genetic, molecular, and physiological data on AM-like associations in liverworts as representative of an ancestral state of land plants [19,28,29].

Surprisingly, given the key phylogenetic position of liverworts in the plant tree of life, very few studies have attempted to identify molecularly the AMF associated with this group, and only a handful of species have been analysed [15,16,26]. To address this striking paucity of data, we performed a molecular analysis of the AM-like associations of globally collected liverwort samples to test the hypothesis that liverworts exhibit specificity towards Glomeraceae, as suggested by previous studies. Our discovery of a predominance of the more ancient, non-Glomeraceae AMF families in these plants when compared to flowering plants supports the notion that liverwort AM-like associations are not derived but ancient and representative of an ancestral symbiotic condition in land plants.

## 2. Methods

### (a) Plant collection

A total of 674 liverwort specimens (mature gametophytes; each specimen consisting of at least five thalli sampled within a population) were collected from 336 sites from 24 countries, covering every continent except Antarctica. Specimens included 72 samples from Haplomitriopsida, 411 samples from Marchantiopsida, and 191 samples from Pelliidae, representing at least 85 species. These included species from two of the three Haplomitriopsida genera (one from each of the two families Treubiaceae and Haplomitriaceae), 19 of 36 Marchantiopsida genera, and 14 of 22 Pelliidae genera [30]. The missing Marchantiopsida genera are almost certainly fungus-free thus our coverage here may be regarded as comprehensive, but it is perhaps less so for the Pelliidae as the missing genera all potentially harbour symbionts. However, these genera are all members of the Fossombroniales or Pallaviciniales which are well represented by other genera. Some samples could only be confidently identified to the level of genus and as such the number of different species likely exceeds 85.

Full details of plants collected and collection locations are provided in electronic supplementary material, table S1. Global collection sites can be seen in electronic supplementary material, figure S1. Plants were named using the latest nomenclature [30] and specimen vouchers have been deposited at the Natural History Museum, London. The liverworts were processed for molecular analysis within three days of collection, prior to which they were stored at 4°C. Plants were cleaned with water and dissected to produce sections of thallus midrib where fungal colonization is highest [20]. Sections *ca* 3 mm^2^ were placed in cetyltrimethylammonium bromide (CTAB) buffer and stored at −20°C.

### (b) Molecular analysis

Liverwort thalli were analysed to identify their symbiotic fungi by amplifying the fungal 18S ribosomal RNA gene using the universal fungal primers NS1 [31] and EF3 [32] and molecular cloning [18,26,33]. Using the 18S ribosomal RNA gene is standard for studies of Glomeromycotina [9]. The use of universal fungal primers maximizes the diversity of Glomeromycotina captured during PCR. Genomic DNA extraction was performed using chloroform [34] combined with the GeneClean II kit (QBioGene) on single thallus sections with leaflets and meristems removed, previously stored in CTAB. For some samples DNA extraction and sequencing were performed more than once (electronic supplementary material table S1). Amplification of fungal DNA used JumpStart (Sigma) and the following PCR settings: 94°C for 2 min followed by 34 cycles of 94°C for 30 s, 53°C for 30 s and 72°C for 1 min 30 s, finishing with 72°C for 7 min. The TOPO TA cloning kit (Invitrogen) was used and 4–8 colonies per sample were DNA sequenced with NS1 using BigDye v3.1 (Applied Biosystems) on an ABI3730 (Applied Biosystems).

Sequences were initially identified using National Center for Biotechnology Information (NCBI) BLAST. Glomeromycotina sequences were selected for sequencing of the rest of the 18S gene using the primers NS3 and NS5 [31]. If more than one of a sample's clones was Glomeromycotina, sequences were aligned and if pairwise similarity was less than 98% then both sequences were selected for full-length sequencing. All alignments were performed using MUSCLE [35] within MEGA7 [36]. Sequence editing and assembly into contigs used Geneious v.7 [37]. Consensus DNA sequences from the contigs were aligned with references from NCBI GenBank, including sequences from spores representing different Glomeromycotina families and Glomeromycotina sequences previously generated from liverworts [19,26] and other early-diverging plant clades: hornworts [18], lycopods, and ferns [33]. We checked for chimeras using UCHIME [38] and UNOISE2 [39]; neither detected chimeras. Evolutionary models were tested in MEGA7. Phylogenies were produced using maximum likelihood in MEGA7 and Bayesian inference with MrBayes [40]. Phylogenetic trees were visualized using FigTree v.1.4 (http://tree.bio.ed.ac.uk/software/figtree). New Glomeromycotina DNA sequences were accessioned in GenBank (MG829276–829601).

### (c) Species delimitation and comparisons to virtual taxa

Species delimitation methods were employed to group DNA sequences. Sequence alignments were run through ALTER to remove haplotypes [41]. Two delimitation approaches were used. The Poisson Tree Processes (PTP) approach delimits species using branch lengths on a rooted phylogenetic tree to infer when speciation events are likely to have taken place [42]. An update of the original PTP method was used, mptp, which uses a maximum likelihood tree as the input and performs Markov chain Monte Carlo (MCMC) sampling to produce support values for the groupings [43]. The input trees were produced using RAxML [44] run on the CIPRES scientific gateway [45]. The RAxML-HPC2 on XSEDE was used with 1 000 bootstrap iterations. The Generalized Mixed Yule Coalescent (GMYC) approach uses an ultrametric, time-calibrated phylogenetic tree to model speciation events and within-population coalescence based on differences in branching rates [46]. Unrooted, ultrametric trees were produced using BEAST2 [47], which was run on CIPRES. BEAST2 analyses used the ‘Relaxed Clock Log Normal’ molecular clock and Birth Death population model. The evolutionary model for each analysis was selected using bModelTest [48]. The MCMC chain length varied between 10 000 000 and 200 000 000 depending on the size of the alignment and the success of convergence. Chain length determined the frequency of tree sampling so on completion of the analysis, 1 000 trees had been sampled. The BEAST outputs were observed in Tracer v.1.6 (http://beast.bio.ed.ac.uk/Tracer) to determine convergence. If all the effective sample size values were greater than 200 the analysis was taken forward, if not then the MCMC chain length was increased and the BEAST analysis run again. The 1 000 output trees produced were converted to one consensus tree using TreeAnnotator with a 10% burn-in with Maximum Clade Credibility as the target tree type and Common Ancestor Heights [47]. TreeAnnotator was run on CIPRES and the output consensus tree produced was viewed using FigTree. The GMYC analysis was performed on the consensus tree in RStudio v.0.99 (https://www.rstudio.com) using the ‘splits’ package. As singletons can influence the analysis, if these were found in GMYC they were removed from the original alignment and the analysis was run again from the beginning [49]. The confidence of the species groupings produced by GMYC was also calculated using ‘splits’. Sequences were assigned to groups called epGT (early-diverging plant Glomeromycotina taxa) by comparing the results of mptp and GMYC. In cases where the analyses did not agree, the confidence levels were compared and the most confident grouping was selected. The reference DNA sequences previously acquired from GenBank were also included in the species delimitation analyses to allow comparisons between AMF in the different plant groups. A species accumulation curve was produced using ‘vegan’ in RStudio and extrapolation was performed using the bootstrap method.

To determine how AMF in liverworts compare to those from previous studies, type DNA sequences for each of the 357 Glomeromycotina virtual taxa were acquired from the MaarjAM database [10]. These type sequences are referred to as GVT (Glomeromycotina virtual taxa) to distinguish them from the taxa found in this study in early-diverging plants (epGT), delimited using a different method. The operational taxonomic unit (OTU) clustering analysis used to assign virtual taxa was performed using CD-HIT [50]. A similarity cut-off of 99% was used to allow proper delimitation of GVT because cut-offs of 97% and 98% grouped many GVT into clusters.

### (d) Ancestral reconstruction

Ancestral state analysis was performed using Mesquite v.3.31 (http://mesquiteproject.org). The character selected for ancestral reconstruction was the presence or absence of ‘colonization by Glomeromycotina fungi that originated before the host’. This character was reconstructed in the major plant lineages; liverworts, hornworts, lycopods, ferns, gymnosperms, and angiosperms. Mosses were not included as they have not been found to enter into mycorrhizal-like symbiosis. In any given plant group, if AM are exclusively formed by a Glomeromycotina lineage that diverged later than the plant group, then a symbiosis with that Glomeromycotina lineage cannot be an ancestral character of the plant group. However, if the plant group is colonized by Glomeromycotina lineages that originated earlier than the plant group, then this can be an ancestral association. For example, if liverworts were exclusively colonized by Glomeraceae, as previously reported [15], then association with Glomeromycotina fungi could not be ancestral because liverworts predate Glomeraceae by at least 100 MY [27]. Glomerales is considered to have an origin less than 400 MYA, while Glomeromycotina originated over 500 MYA, before plant terrestrialization [12]. Plant group ages were based on the monophyletic scenario of Morris *et al.* [5]: hornworts, 506–460 MY; liverworts, 443–405 MY; lycopods, 432–392 MY; ferns, 411–384 MY; gymnosperms, 337–308 MY; angiosperms, 246–197 MY. In all error ranges, liverworts and hornworts evolved after the origin of Glomeromycotina, but before Glomerales divergence. The character of whether the symbiotic Glomeromycotina predates the plant group was scored twice, once based on knowledge before this study and once on new understanding. Results for hornworts, lycopods, and ferns are from reanalysis of previous data [18,33].

Prior to ancestral reconstruction, a phylogenetic tree was produced using the *rbcL* gene of a liverwort (*Marchantia paleacea*—DQ286015), hornwort (*Anthoceros punctatus*—U87063.1), lycopod (*Lycopodium clavatum*—AB574626), fern (*Psilotum nudum*—KR816696.1), gymnosperm (*Prumnopitys taxifolia*—AF249658.1), and an angiosperm (*Nicotiana tabacum*—KC825342.1). Sequences were aligned in MEGA7 and a phylogeny produced in MrBayes using the nst = 2 model, gamma rates and 10 000 000 MCMC generations. The tree was visualized in Mesquite using the mirror tree window. Ancestral reconstruction used the maximum-likelihood Markov 1-parameter model, with default settings, selected based on the Asymmetry Likelihood Ratio Test within Mesquite.

### (e) Cytology

Representatives of Marchantiopsida and Pelliidae liverworts shown by molecular analyses to harbour Glomeromycotina symbionts (electronic supplementary material, table S1) were processed for standard and cryo-scanning electron microscopy (SEM, cryo-SEM). Preparation of samples followed established protocols for SEM [16] and cryo-SEM [51]. Ultrastructural analyses of the fungal symbionts of Haplomitriopsida liverworts were not performed as these have been described extensively before [22] and these plants are not colonized by Glomeromycotina fungi (electronic supplementary material, table S1).

## 3. Results

### (a) Glomeromycotina in liverworts

**Figure 1.**
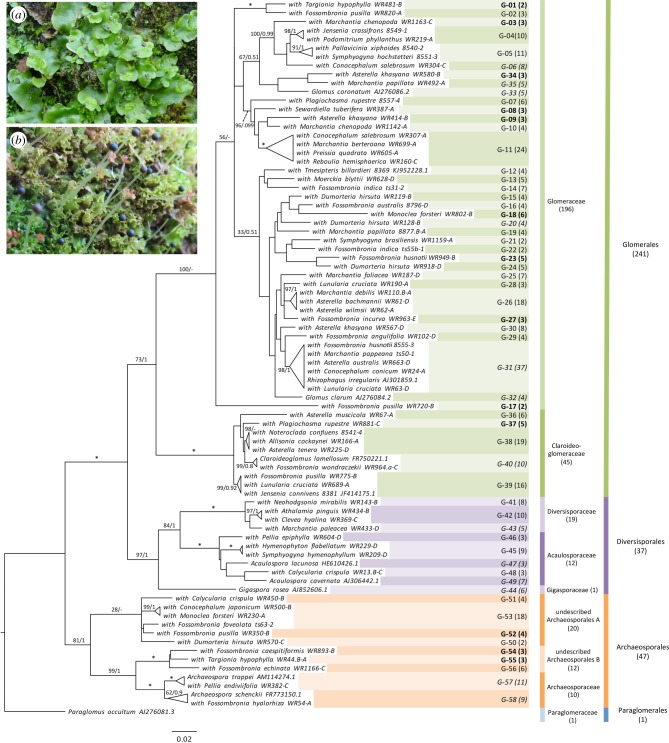
Glomeromycotina in liverworts. Maximum-likelihood tree summarizing fungi detected in liverworts, produced using the 18S gene. Sequences have been delimited into 58 epGT (shortened to G in the figure). The number in brackets after the epGT is the number of sequences contained in that group. Those in bold are specific to liverworts and contained no sequences from other plants, Glomeromycotina spores, or GVT. The italicized epGT contain sequences from Glomeromycotina spores. The number of new liverwort sequences for each Glomeromycotina family and order is given in brackets. Support values give the results of maximum-likelihood bootstrapping and Bayesian inference. Only support values for the main branches and clades that contain more than one epGT sequence are shown. An asterisk indicates full support (100/1) from both analyses while a dash signifies the grouping was not found in Bayesian inference. Inset are examples of Marchantiopsida and Pelliidae liverworts; (*a*) *Lunularia cruciata*, (*b*) *Fossombronia echinata*.

A total of 257 liverwort specimens from 21 countries and 182 collection locations, representing 55 species, were colonized by Glomeromycotina. Full details of the liverworts analysed are given in electronic supplementary material, table S1 and summarized in electronic supplementary material, table S2. Other fungi identified were members of Ascomycota (most frequently Helotiales), Basidiomycota, Chytridiomycota, Mortierellomycotina, and Mucoromycotina. Glomeromycotina colonized 40% of Marchantiopsida samples and 49% of Pelliidae (electronic supplementary material, table S3) but were not found in Haplomitriopsida. Every order of Glomeromycotina was detected in Marchantiopsida, and three in Pelliidae. Simultaneous colonization of the same thallus section by different AMF families occurred in 23 samples. The liverwort model genus *Marchantia* was colonized by members of the Claroideoglomeraceae, Diversisporaceae, and Archaeosporaceae in addition to diverse Glomeraceae. The phylogenetic position of the 326 DNA sequences produced is shown in electronic supplementary material, figure S2 and summarized in figure 1 (support values are provided in electronic supplementary material, figures S3 and S4).

Glomeromycotina DNA sequences from liverworts and other early-diverging plant clades (hornworts [18], lycopods, and ferns [33]) were delimited into 58 ‘species’ groups, or epGT (figure 1). An OTU cluster analysis of the sequences used to produce epGT and the 357 type representatives of GVT found only 45 GVT cluster with the Glomeromycotina sequences from early-diverging plants (electronic supplementary material, table S4). When GVT sequences were included in the GMYC/mptp species delimitation analysis, only 33 of the 58 epGTs were found to contain GVT representatives. The remaining 25 epGTs are newly discovered and exclusive to early-diverging plants (including 13 exclusive to liverworts). In addition to the 58 epGT, 36 Glomeromycotina sequences from liverworts were delimited as singletons. As these were unambiguous, they can also be considered true taxa, as shown by six of these singletons clustering with GVT. Accordingly, the number of fungal taxa in early-diverging plants increases to 94 and the number of specific early-diverging taxa to 55, 43 of which were liverwort-specific. Extrapolation of a species accumulation curve (electronic supplementary material, figure S5) suggests that between 82% (when including singletons) and 89% (when only including epGT) of the taxa that colonize these liverworts has been detected.

### (b) Non-Glomeraceae AMF in liverworts and ancestral reconstruction

Using available published data from relevant surveys of AMF in flowering plants, we calculated the proportion of non-Glomeraceae DNA sequences and, where possible, the proportion of samples colonized by AMF exclusively harbouring non-Glomeraceae fungi (electronic supplementary material, table S5). In liverworts, these values are 40% and 36%, respectively; the difference between these values reflects some liverworts being colonized by both Glomeraceae and non-Glomeraceae fungi. In two studies using similar molecular cloning to ours, the proportion of non-Glomeraceae sequences in flowering plants were 7% [52] and 4% [53] (electronic supplementary material, table S5). Furthermore, a global survey of AMF in 153 flowering plant species (1 014 samples) that used 454 DNA sequencing found only 0.2% of plant samples were exclusively colonized by non-Glomeraceae AMF and only 6% of AMF DNA sequences were non-Glomeraceae [11] (electronic supplementary material, table S5). We analysed data from 11 different studies of flowering plants and found that all reported similar proportions of non-Glomeraceae sequences across methods (mean: 4%, range: 1–7%, electronic supplementary material, table S5). The non-Glomeraceae detection rate was significantly higher in liverworts than in all 11 studies of flowering plants, with categorical *Z*-test *p*-values of less than 0.00001 in all cases.

Ancestral state reconstruction using the data produced in this study and reanalysis of previous studies on hornworts [18], lycopods, and ferns [33] provides significant support for an association with Glomeromycotina representing an ancestral state for all land plants (electronic supplementary material, figure S6). This notion is supported by the colonization of liverworts, hornworts, and ferns by lineages of Glomeromycotina that diverged earlier than these plant groups (electronic supplementary material, figure S6B).

### (c) Cytology of Glomeromycotina associations in liverworts

**Figure 2.**
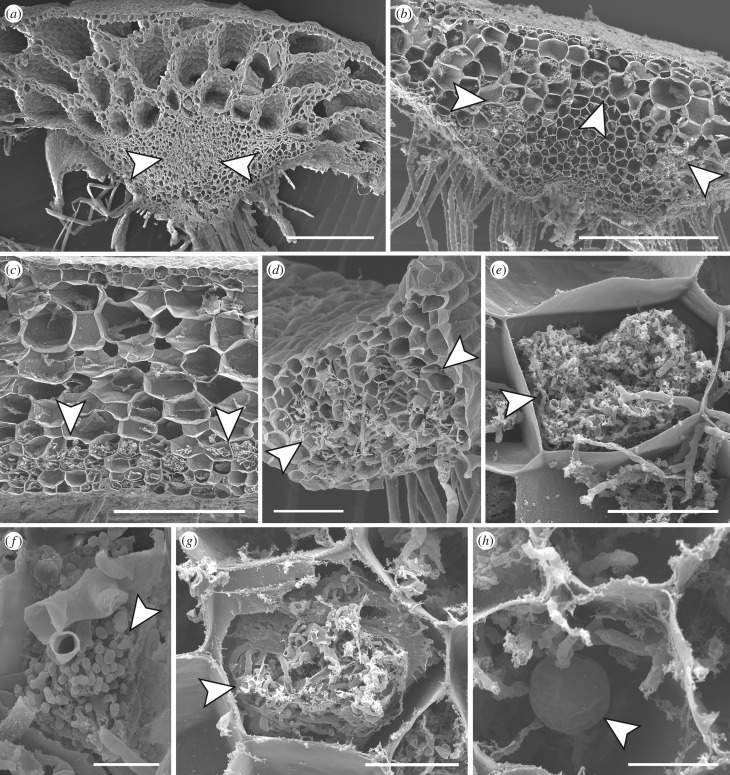
Cytology of Glomeromycotina associations in liverworts. Scanning electron micrographs of cross sections through thalli of (*a*) *Neohodgsonia mirabilis*; (*b*,*f*,*g*) *Dumortiera hirsuta*; (*c*,*e*) *Monoclea forsteri*; (*d*) *Fossombronia foveolata*; (*f*) *Marchantia pappeana*. Fungal colonization usually occupies the thallus central midrib (arrowed) (*a*,*d*). In some Marchantiopsida liverworts colonization is sometimes confined to a region overarching the midrib hyaline strand (arrowed) (*b*), or is restricted to the thallus ventral cell layers (arrowed) (*c*). Fungal structures include arbusculated coils (arrowed) (*e*), arbuscules terminal on trunk hyphae (arrowed) (*f*), coils (arrowed) (*g*), and vesicles of varying diameters (arrowed) (*h*). Scale bars: (*a–c*) 500 µm; (*d*) 100 µm; (*e*) 50 µm; (*g*,*h*) 20 µm; (*f*) 10 µm.

Marchantiopsida and Pelliidae liverworts lack intercellular spaces in their thalli [54], therefore fungal colonization is strictly intracellular. Fungal colonization in Marchantiopsida occupies specific regions of the thallus; the central midrib (figure 2*a*; electronic supplementary material, figures S7A and S8D), sometimes overarching the midrib hyaline strand (figure 2*b*; electronic supplementary material, figure S8A), or the ventral cell layers (figure 2*c*; electronic supplementary material, figure S8E,F). Our results confirm reports of distinct zonation of fungi in some Marchantiopsida [16], with coils and vesicles predominantly occupying a lower area in the thallus and arbuscules proliferating above, but not in several others where the distribution of fungal structures appears less structured (e.g. electronic supplementary material, figure S7A,E). This was also observed in Pelliidae (figure 2*d*; electronic supplementary material, figure S10A,G), where fungal colonization generally occupies the central midrib area of the thallus. Glomeromycotina structures in Marchantiopsida and Pelliidae included terminal arbuscules on trunk hyphae (figure 2*f*; electronic supplementary material, figure S10J), arbusculated coils with intercalary arbuscules (figure 2*e*; electronic supplementary material, figure S9C) and coils (figure 2*g*; electronic supplementary material, figure S10E,I). In both Marchantiopsida and Pelliidae, arbuscules and/or arbusculated coils were constant features of Glomeromycotina colonization (electronic supplementary material, table S6). Vesicles of varying size were also common, but inconsistent (figure 2*g*), ranging from 8 µm to 45 µm depending on the species, with the biggest range within *Fossombronia* (14–30 µm) (electronic supplementary material, table S6). *Fossombronia* also produced the greatest range of structures (electronic supplementary material, figure S10B–F), including tightly wound coils with hyphal diameters less than 2 µm and small terminal swellings—in line with detection of the most diverse epGT in this genus. Overall, our results greatly extend previous observations of colonization by Glomeromycotina in Marchantiopsida and Pelliidae liverworts [16,17,20], and the following general features were observed: (i) fungal entry is invariably via the rhizoids whereas the other epidermal cells are fungus-free (electronic supplementary material, figure S8G), (ii) fungal structures are consistently absent from meristematic cells, storage organs, and oil bodies (electronic supplementary material, figure S8H), (iii) colonized host cells generally contain considerably less starch deposits than non-colonized cells, (iv) as in vascular plants, the fungi exhibit multiple waves of colonization and degeneration, (v) the mosaics of colonized and fungus-free cells seen in some leafy liverworts with basidiomycetous symbionts are absent, and (vi) the overgrowths of host cell walls that restrict the spread of hyphae into uncolonized cells seen in many leafy liverworts [20], were not encountered.

## 4. Discussion

We found that liverworts, some of the closest living relatives of the first land plants, contrary to previous reports, are more frequently associated with ancient lineages of AMF than flowering plants and harbour diverse, newly described and specific symbionts. Thus, we can reject the hypothesis that liverworts exhibit specificity towards Glomeraceae [15].

### (a) Ancient fungi in ancient plants

Our study shows for the first time that liverworts can be colonized by the full diversity of Glomeromycotina and are not limited to a narrow set. While Glomeraceae were the most frequently detected symbionts, being represented by 55 of 94 taxa encountered (35 epGT and 20 singletons), colonization by Glomeraceae is much lower in liverworts compared to flowering plants. In flowering plants *ca* 96% of AMF are reported to be members of the Glomeraceae, but in liverworts this value is only 60%, the remaining 40% consisting of non-Glomeraceae AMF (electronic supplementary material, table S5). This comparison of flowering plants and liverworts is complicated by a lack of uniform DNA sequencing methods across surveys or a global survey of AMF using the molecular cloning method of our study (electronic supplementary material, table S5). However, this does not invalidate comparisons as the values for flowering plants were consistently close to 4% across studies, regardless of the method used. There is an order of magnitude difference between non-Glomeraceae colonization rates; while the precise difference is to be determined, it is clear that liverworts and hornworts are considerably more colonized by ‘older’ Glomeromycotina than flowering plants. The molecular cloning method may underestimate the diversity of AMF that colonize plants; however, it appears the vast majority of AMF that colonize liverworts has been detected (electronic supplementary material, figure S5).

The widespread occurrence in liverworts of colonization by members of more ancient families of Glomeromycotina together with the results of ancestral reconstruction (electronic supplementary material, figure S6), support the hypothesis that liverwort–Glomeromycotina partnerships represent an ancestral symbiotic condition. Despite being a widely held hypothesis, evidence in support of AM as an ancestral state for all land plants—including conservation of symbiosis genes [55], congruence between the origin of liverworts and Glomeromycotina [12], and exceptionally broad host range of AMF [56]—has been partial and fossils of the first land plants (that could be used to unequivocally confirm an association) do not exist. In fact, the available molecular evidence only supports AM as ancestral in seed plants (electronic supplementary material, figure S6A), and fossil evidence of arbuscule-like structures in the early vascular plant *Aglaophyton major* [57] only supports Glomeromycotina colonization as an ancestral character of vascular plants. Thus, there is a discrepancy between the widely accepted hypothesis and the available data [15,16,19,26]. Our discovery of widespread colonization by non-Glomeraceae fungi in liverworts and other early-diverging plant clades removes this discrepancy and lends considerable weight to the hypothesis that Glomeromycotina associations are ancestral in liverworts and all extant land plants. In the absence of fossils of the earliest land plants showing associations with Glomeromycotina, these molecular results represent some of the best available evidence in support of this hypothesis. The absence of early fossils showing plant–fungal symbiosis and enough Glomeromycotina calibration points prevent robust comparisons of land plant and Glomeromycotina diversifications.

Inferring an ancestral association with Glomeromycotina through the abundance of non-Glomeraceae in liverworts is more appropriate than the alternative interpretation of these results, i.e. that Glomeromycotina in liverworts are recent and the result of host shifts from flowering plants [27]. If this were the case, we would expect to detect (i) only a subset of derived Glomeromycotina in liverworts and hornworts, and/or (ii) similar dominance of the Glomeraceae in liverworts and hornworts as seen in flowering plants. Both disagree sharply with our global analysis (figure 1). Furthermore, mechanisms would be needed for earlier-diverging lineages of Glomeromycotina becoming more prevalent in liverworts and hornworts than flowering plants despite having been acquired from flowering plants, and for Glomeraceae failing to fully dominate liverworts and hornworts as it does in flowering plants. We are not aware of evidence of such mechanisms. Therefore, the recent-origin hypothesis [27] is unlikely, and it can no longer use Glomeraceae-specificity in liverworts as its key argument.

Nonetheless, it is possible that some early-diverging members of the Glomeromycotina have been replaced by more derived AMF in liverworts as a result of host-shifting from flowering plants. This is expected given the global dominance of flowering plants, in which Glomeraceae is prevalent, during the last 100 MY (electronic supplementary material, table S5). For example, the single most frequently detected epGT was *Rhizophagus irregularis* (epGT31, figure 1)—the most common and well-studied AMF in flowering plants [58] and globally dominant in all land plant lineages.

### (b) Novel and liverwort-specific AMF taxa

Comparisons of AMF that colonize liverworts worldwide with those in higher plants using MaarjAM, the most comprehensive AMF database [10], uncovered a hitherto ‘hidden’ diversity of Glomeromycotina in early-diverging land plants. These analyses revealed 55 new AMF taxa that are apparently unique to early-diverging land plants (liverworts, hornworts, lycopods, and ferns). These taxa were regularly found throughout the plants analysed and the second most commonly detected taxon (epGT38—Claroideoglomeraceae) was unique to liverworts and hornworts. It is unclear how much of this novel AMF diversity detected results from analysing new plants and how much from sampling new locations. Given 20 of the 24 countries sampled here are already in MaarjAM, and among the other four countries, only Ascension Island contained a country-specific epGT, new countries seem unlikely to have had a large influence on the diversity detected. The potential influence of habitat on AMF diversity and proportion of new taxa detected, as suggested for higher plants [59] is more difficult to determine in liverworts. Lack of fungal colonization can certainly be explained by habitat preference; Glomeromycotina are absent in saxicolous (e.g. Cyathodiaceae) and submerged aquatic (e.g. *Monoselenium*) genera, and populations growing in very wet habitats (*Conocephalum* spp*.*). However, thalloid liverworts (and hornworts) tend to occupy specific microhabitats, thus habitat-specificity by bryophyte-Glomeromycotina, if present, is likely to occur at a finer spatial scale than in vascular plants (see electronic supplementary material for more information). While this certainly deserves further investigation now, it was beyond the scope of our study.

### (c) Comparisons to other ancient plant lineages

Reanalysis of a previous study of Glomeromycotina in hornworts [18] revealed that hornworts are even more frequently colonized by members of non-Glomeraceae families than liverworts. Of 86 hornwort samples, 52% were colonized exclusively by non-Glomeraceae versus 36% in liverworts (electronic supplementary material, table S5). Remarkably, unlike for liverworts, none of the epGT were hornwort-specific, suggesting these plants are opportunistic in their symbioses with AMF. In contrast with liverworts and hornworts, early-diverging vascular plants (lycopods and ferns) tend to associate preferentially with members of the Glomeraceae (electronic supplementary material, table S5). Lycopods typically have limited fungal colonization and molecular methods have exclusively detected Glomeraceae fungi [33,60], in addition to Mucoromycotina, though sample sizes have been small. Ferns have exclusive or near-exclusive Glomeraceae colonization [33,61]; a recent analysis of 19 fern species had a non-Glomeraceae AMF detection rate of 12.5% [33], intermediate between non-vascular and flowering plants.

### (d) Cytology of Glomeromycotina associations in liverworts

Marchantiopsida and Pelliidae liverworts contain a considerable diversity of fungal colonization patterns and structures that parallels that in AM of flowering plants [17,62]. The latter fall into *Arum*- and *Paris*-types, with a key distinguishing feature being intercellular hyphae in the *Arum*- but not the *Paris*-type, alongside arbuscules terminal on trunk hyphae in the former and extensive arbusculated coils in the latter [62]. Liverworts, except Haplomitriopsida, do not develop gametophytic intercellular spaces [54] so AMF colonization in liverworts is considered *Paris*-type [16]. However, discounting intercellular hyphae, our results indicate the structures produced by Glomeromycotina in liverworts encompass all those of both types. As with flowering plants [62], colonization can exhibit distinct zonation of fungal structures or not. But, while in flowering plants AMF do not always produce arbuscules/arbusculated coils [62], in all liverworts analysed the presence of either or both of these structures was ubiquitous.

## Supplementary Material

Supplementary Figures S1-10

## Supplementary Material

Supplementary Tables S1-S6

## Supplementary Material

Habitats: Ecological and morphological considerations

## Data accessibility

New sequences have been accessioned in GenBank (MG829276 – 829601). The sequence alignment and newick file used to produce Figure 1 have been archived in the Dryad Digital Repository: https://doi.org/10.5061/dryad.n4709m8 [63].
