# Supplementary material for: Ancient plants with ancient fungi: liverworts associate with early-diverging arbuscular mycorrhizal fungi
Source: Proc Biol Sci. 2018 Oct 10;285(1888):20181600. doi: 10.1098/rspb.2018.1600 (PMC6191707; doi:10.1098/rspb.2018.1600)
Supplement: Supplementary Figures S1-10 [file rspb20181600supp1.pdf]

Supplementary material

Figures S1 to S10

Figure S1. Global map showing liverwort collection sites. Collection sites are highlighted in green. Map produced in RStudio using the 'ggmap' package.

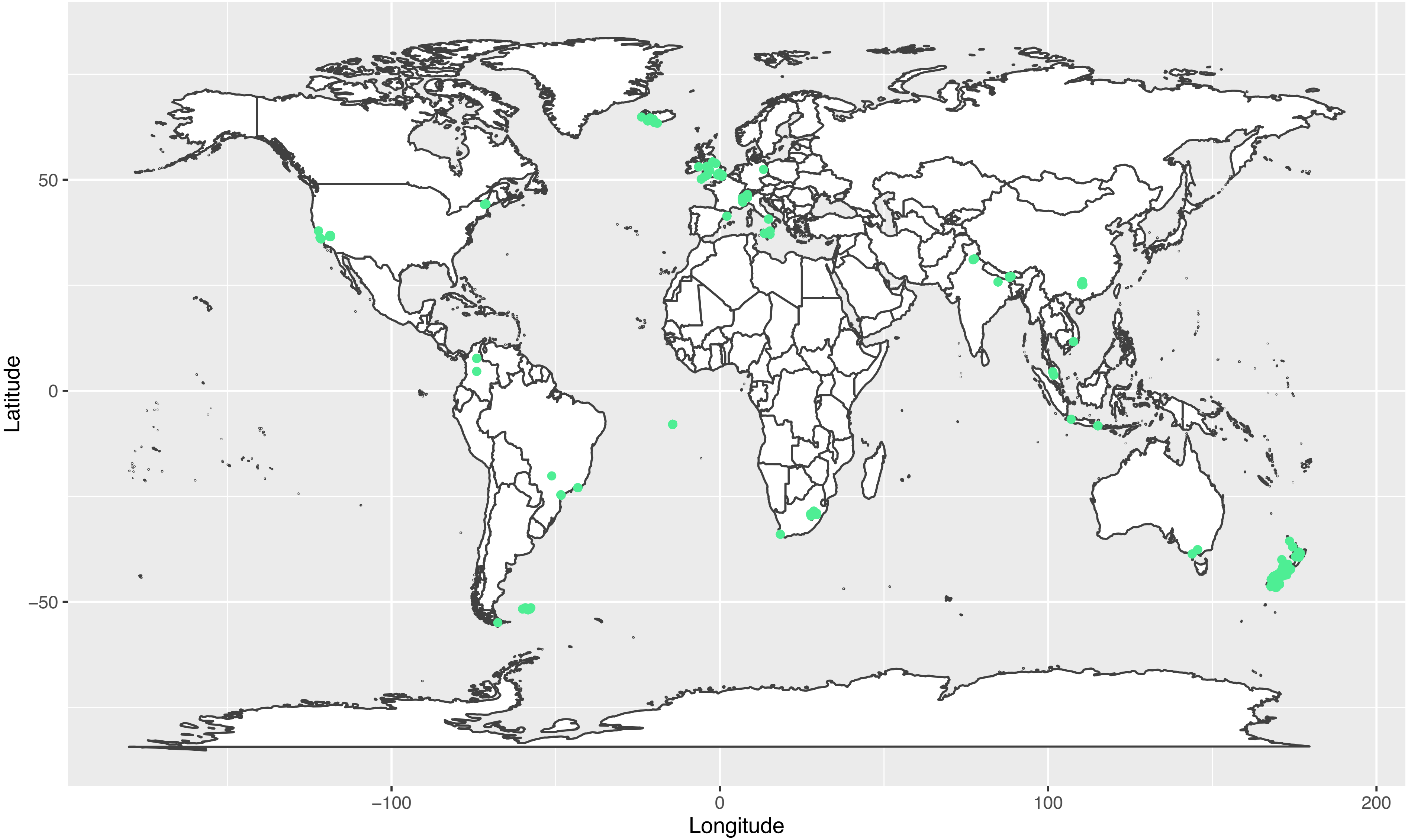

Latitude
50
0
−50
Longitude
−100
0
100
200

Figure S2. Maximum likelihood analysis of Glomeromycotina sequences. All 326 new sequences produced in this study are included in this phylogeny with reference sequences from Glomeromycotina spores. Maximum likelihood was run with 1,000 bootstrap replicates and a generalised time reversible model with invariant gamma rates. An asterisk indicates the DNA sequence is not the full-length 18S.

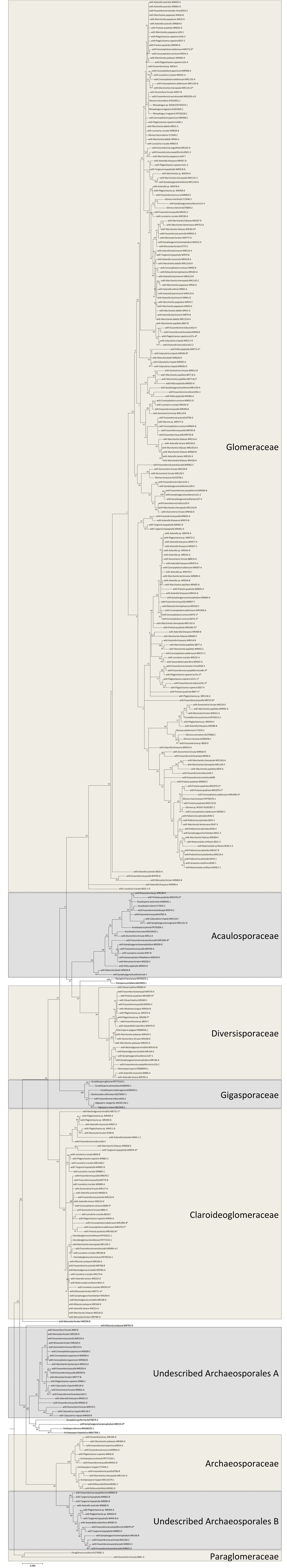
Glomeraceae
Acaulosporaceae
Diversisporaceae
Gigasporaceae
Claroideoglomeraceae
Undescribed Archaeosporales A
Archaeosporaceae
Undescribed Archaeosporales B
Paraglomeraceae
0.050

Figure S3. Summary maximum likelihood phylogeny of Glomeromycotina fungi.

This phylogenetic tree was used to produce figure 1. The analysis used a generalised time reversible model with invariant gamma rates and 1,000 bootstrap replicates.

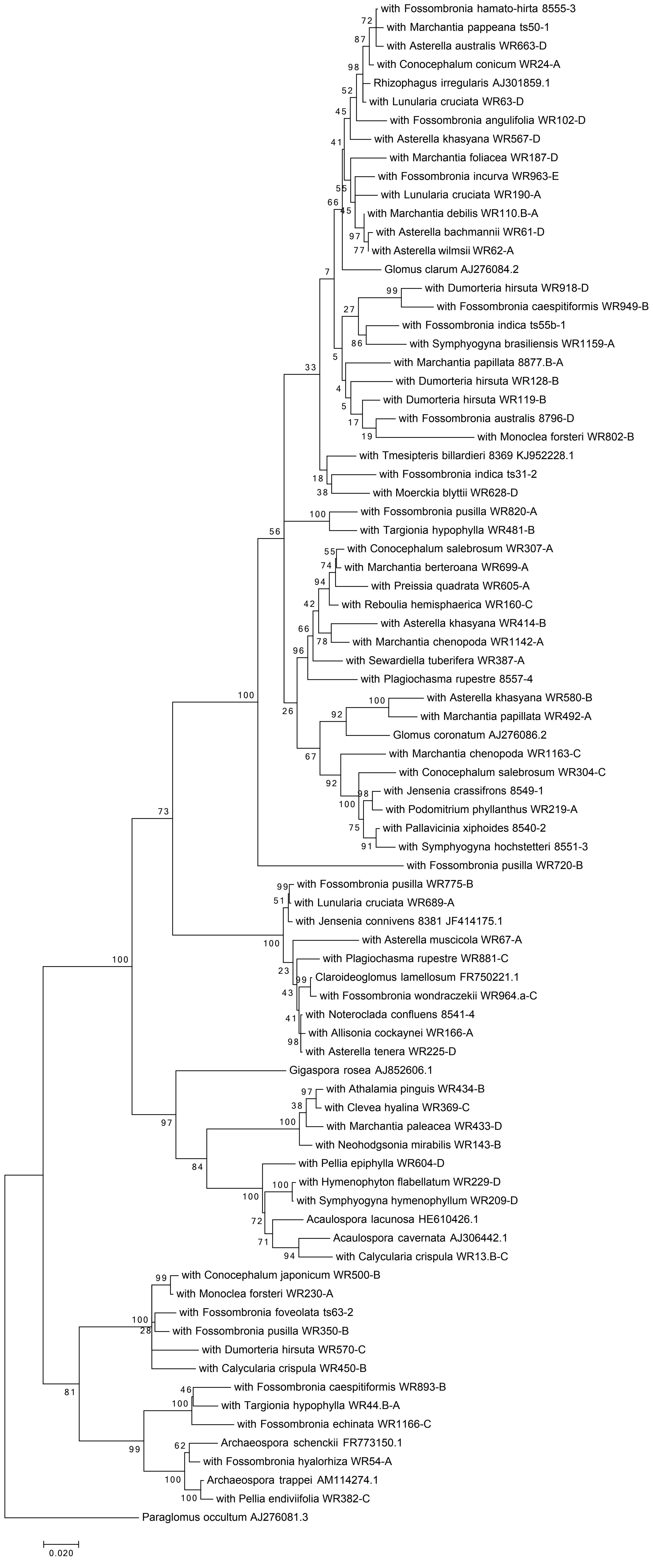

with Fossombronia hamato-hirta 8555-3
with Marchantia pappeana ts50-1
with Asterella australis WR663-D
with Conocephalum conicum WR24-A
Rhizophagus irregularis AJ301859.1
with Lunularia cruciata WR63-D
with Fossombronia angulifolia WR102-D
with Asterella khasyana WR567-D
with Marchantia foliacea WR187-D
with Fossombronia incurva WR963-E
with Lunularia cruciata WR190-A
with Marchantia debilis WR110.B-A
with Asterella bachmannii WR61-D
with Asterella wilmsii WR62-A
Glomus clarum AJ276084.2
with Dumorteria hirsuta WR918-D
with Fossombronia caespitiformis WR949-B
with Fossombronia indica ts55b-1
with Symphyogyna brasiliensis WR1159-A
with Marchantia papillata 8877.B-A
with Dumorteria hirsuta WR128-B
with Dumorteria hirsuta WR119-B
with Fossombronia australis 8796-D
with Monoclea forsteri WR802-B
with Tmesipteris billardieri 8369 KJ952228.1
with Fossombronia indica ts31-2
with Moerckia blyttii WR628-D
with Fossombronia pusilla WR820-A
with Targionia hypophylla WR481-B
with Conocephalum salebrosum WR307-A
with Marchantia berteroana WR699-A
with Preissia quadrata WR605-A
with Reboulia hemisphaerica WR160-C
with Asterella khasyana WR414-B
with Marchantia chenopoda WR1142-A
with Sewardiella tuberifera WR387-A
with Plagiochasma rupestre 8557-4
with Asterella khasyana WR580-B
with Marchantia papillata WR492-A
Glomus coronatum AJ276086.2
with Marchantia chenopoda WR1163-C
with Conocephalum salebrosum WR304-C
with Jensenia crassifrons 8549-1
with Podomitrium phyllanthus WR219-A
with Pallavicinia xiphoides 8540-2
with Symphyogyna hochstetteri 8551-3
with Fossombronia pusilla WR720-B
with Fossombronia pusilla WR775-B
with Lunularia cruciata WR689-A
with Jensenia connivens 8381 JF414175.1
with Asterella muscicola WR67-A
with Plagiochasma rupestre WR881-C
Claroideoglomus lamellosum FR750221.1
with Fossombronia wondraczekii WR964.a-C
with Noteroclada confluens 8541-4
with Allisonia cockaynei WR166-A
with Asterella tenera WR225-D
Gigaspora rosea AJ852606.1
with Athalamia pinguis WR434-B
with Clevea hyalina WR369-C
with Marchantia paleacea WR433-D
with Neohodgsonia mirabilis WR143-B
with Pellia epiphylla WR604-D
with Hymenophyton flabellatum WR229-D
with Symphyogyna hymenophyllum WR209-D
Acaulospora lacunosa HE610426.1
Acaulospora cavernata AJ306442.1
with Calycularia crispula WR13.B-C
with Conocephalum japonicum WR500-B
with Monoclea forsteri WR230-A
with Fossombronia foveolata ts63-2
with Fossombronia pusilla WR350-B
with Dumorteria hirsuta WR570-C
with Calycularia crispula WR450-B
with Fossombronia caespitiformis WR893-B
with Targionia hypophylla WR44.B-A
with Fossombronia echinata WR1166-C
Archaeospora schenckii FR773150.1
with Fossombronia hyalorhiza WR54-A
Archaeospora trappei AM114274.1
with Pellia endiviifolia WR382-C
Paraglomus occultum AJ276081.3
72
87
98
52
45
41
55
66
45
97
77
7
99
27
86
5
4
5
17
19
33
18
38
100
56
55
74
94
42
66
78
96
100
100
92
26
67
92
98
100
75
91
73
99
51
100
23
99
43
41
98
100
97
38
100
97
84
100
100
72
71
94
99
100
28
81
46
100
99
62
100
100
0.020

Figure S4. Summary Bayesian inference phylogeny of Glomeromycotina fungi. This phylogenetic tree was used to provide support values for Figure 1. The MrBayes analysis was run for 10,000,000 million generations with the nst=6 model and invgamma rates.

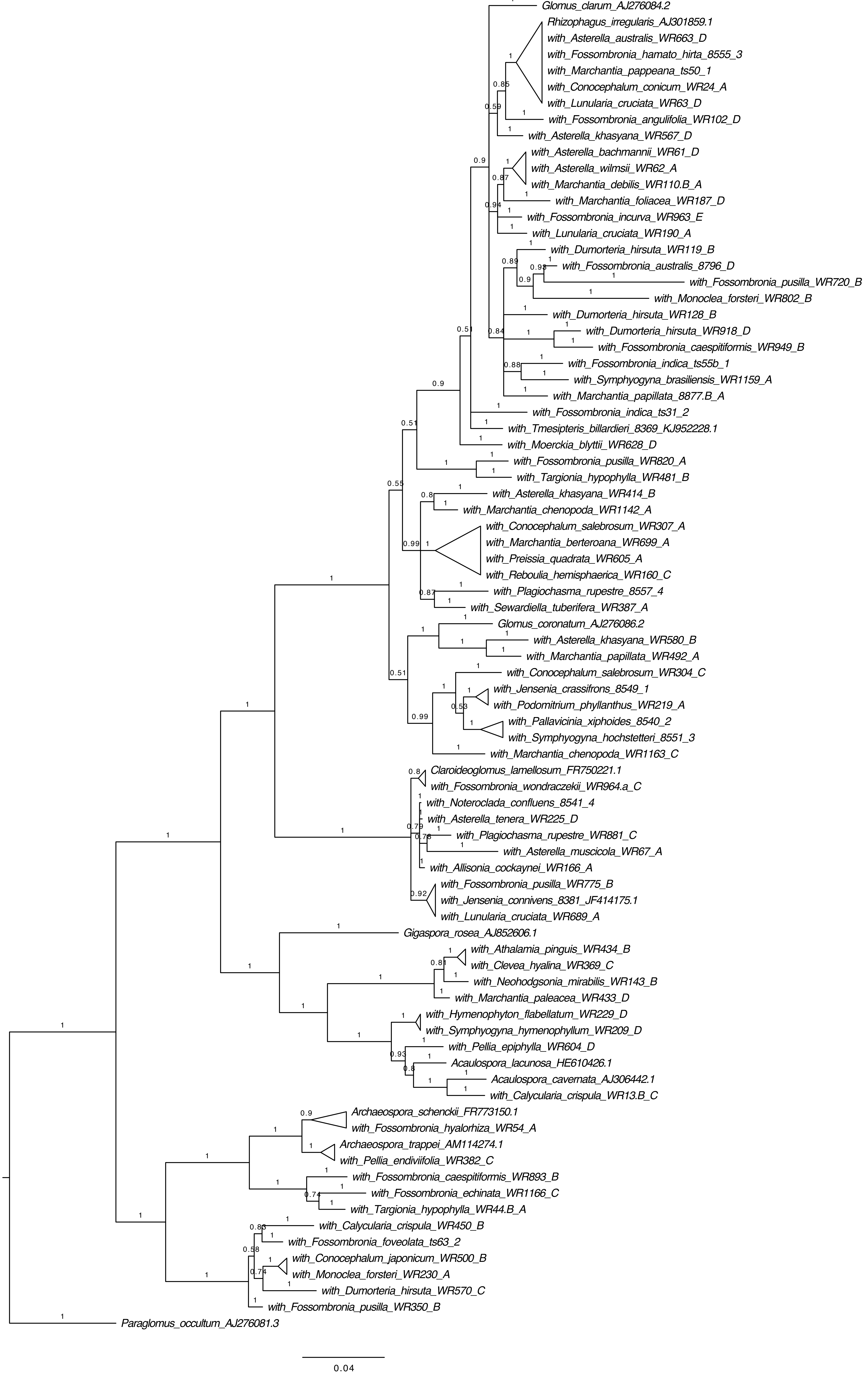
Glomus_clarum_AJ276084.2
Rhizophagus_irregularis_AJ301859.1
with_Asterella_australis_WR663_D
with_Fossombronia_hamato_hirta_8555_3
with_Marchantia_pappeana_ts50_1
with_Conocephalum_conicum_WR24_A
with_Lunularia_cruciata_WR63_D
with_Fossombronia_angulifolia_WR102_D
with_Asterella_khasyana_WR567_D
with_Asterella_bachmannii_WR61_D
with_Asterella_wilmsii_WR62_A
with_Marchantia_debilis_WR110.B_A
with_Marchantia_foliacea_WR187_D
with_Fossombronia_incurva_WR963_E
with_Lunularia_cruciata_WR190_A
with_Dumorteria_hirsuta_WR119_B
with_Fossombronia_australis_8796_D
with_Fossombronia_pusilla_WR720_B
with_Monoclea_forsteri_WR802_B
with_Dumorteria_hirsuta_WR128_B
with_Dumorteria_hirsuta_WR918_D
with_Fossombronia_caespitiformis_WR949_B
with_Fossombronia_indica_ts55b_1
with_Symphyogyna_brasiliensis_WR1159_A
with_Marchantia_papillata_8877.B_A
with_Fossombronia_indica_ts31_2
with_Tmesipteris_billardieri_8369_KJ952228.1
with_Moerckia_blyttii_WR628_D
with_Fossombronia_pusilla_WR820_A
with_Targionia_hypophylla_WR481_B
with_Asterella_khasyana_WR414_B
with_Marchantia_chenopoda_WR1142_A
with_Conocephalum_salebrosum_WR307_A
with_Marchantia_berteroana_WR699_A
with_Preissia_quadrata_WR605_A
with_Reboulia_hemisphaerica_WR160_C
with_Plagiochasma_rupestre_8557_4
with_Sewardiella_tuberifera_WR387_A
Glomus_coronatum_AJ276086.2
with_Asterella_khasyana_WR580_B
with_Marchantia_papillata_WR492_A
with_Conocephalum_salebrosum_WR304_C
with_Jensenia_crassifrons_8549_1
with_Podomitrium_phyllanthus_WR219_A
with_Pallavicinia_xiphoides_8540_2
with_Symphyogyna_hochstetteri_8551_3
with_Marchantia_chenopoda_WR1163_C
Claroideoglomus_lamellosum_FR750221.1
with_Fossombronia_wondraczekii_WR964.a_C
with_Noteroclada_confluens_8541_4
with_Asterella_tenera_WR225_D
with_Plagiochasma_rupestre_WR881_C
with_Asterella_muscicola_WR67_A
with_Allisonia_cockaynei_WR166_A
with_Fossombronia_pusilla_WR775_B
with_Jensenia_connivens_8381_JF414175.1
with_Lunularia_cruciata_WR689_A
Gigaspora_rosea_AJ852606.1
with_Athalamia_pinguis_WR434_B
with_Clevea_hyalina_WR369_C
with_Neohodgsonia_mirabilis_WR143_B
with_Marchantia_paleacea_WR433_D
with_Hymenophyton_flabellatum_WR229_D
with_Symphyogyna_hymenophyllum_WR209_D
with_Pellia_epiphylla_WR604_D
Acaulospora_lacunosa_HE610426.1
Acaulospora_cavernata_AJ306442.1
with_Calycularia_crispula_WR13.B_C
Archaeospora_schenckii_FR773150.1
with_Fossombronia_hyalorhiza_WR54_A
Archaeospora_trappei_AM114274.1
with_Pellia_endiviifolia_WR382_C
with_Fossombronia_caespitiformis_WR893_B
with_Fossombronia_echinata_WR1166_C
with_Targionia_hypophylla_WR44.B_A
with_Calycularia_crispula_WR450_B
with_Fossombronia_foveolata_ts63_2
with_Conocephalum_japonicum_WR500_B
with_Monoclea_forsteri_WR230_A
with_Dumorteria_hirsuta_WR570_C
with_Fossombronia_pusilla_WR350_B
Paraglomus_occultum_AJ276081.3
0.04

Figure S5. Species accumulation curve of Glomeromycotina that colonise liverworts. In blue are the results of using epGT and singletons and in red is the curve of only using epGT. The confidence intervals from 1,000 random permutations can be seen in the highlighted areas.

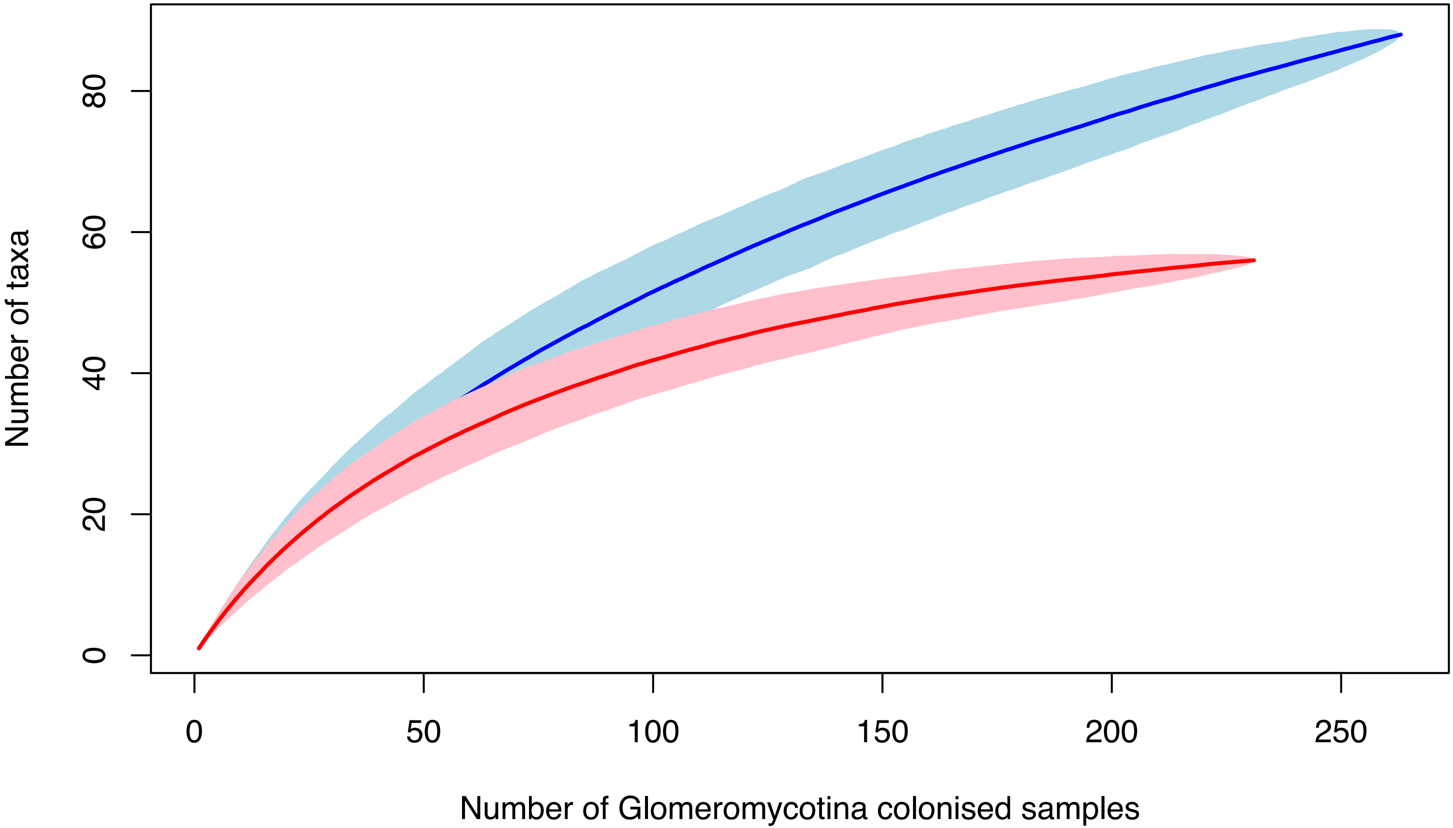

Number of taxa
0
20
40
60
80
0
50
100
150
200
250
Number of Glomeromycotina colonised samples

Figure S6. Ancestral reconstruction of early-diverging Glomeromycotina lineages in plants. Black circles indicate the plant group has been shown, using molecular methods, to contain Glomeromycotina fungi that originated before the plant while white indicates they have not. (a) Our state of knowledge before this study and (b) our knowledge now.

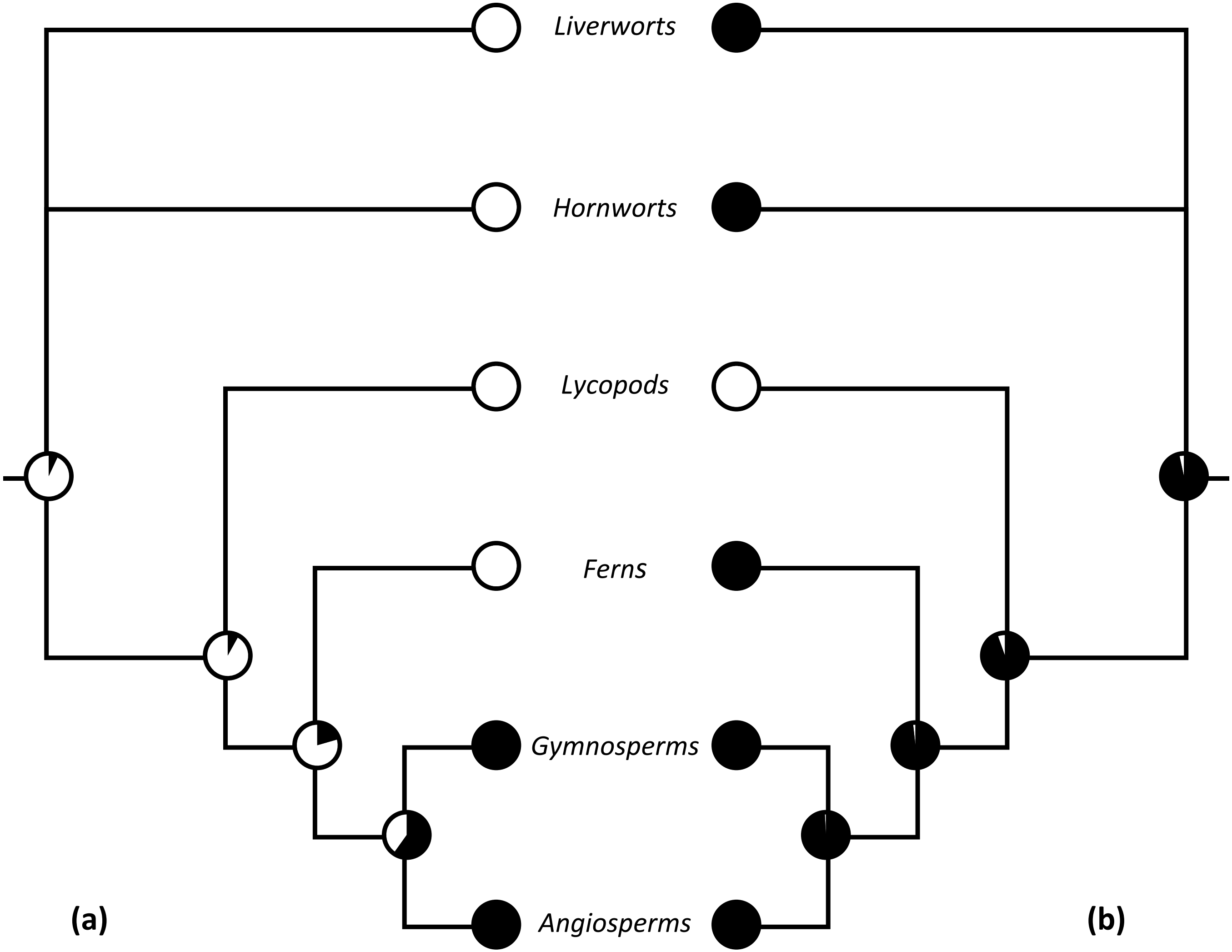
Liverworts
Hornworts
Lycopods
Ferns
Gymnosperms
Angiosperms
(a)
(b)

Figure S7. Cytology of Glomeromycotina associations in Marchantiopsida (complex thalloid) liverworts. Scanning electron micrographs of cross sections through thalli of (A) *Neohodgsonia mirabilis*; (B, C) *Lunularia cruciata*; (D, E) *Marchantia pappeana*; (F) *M. foliacea*; (G) *M. paleacea*. Fungal colonisation usually occupies the thallus central midrib (A, E, F, G) and is sometimes confined to a region overarching the midrib hyaline strand (F). In some taxa, e.g. *L. cruciata* (B), fungal colonisation is restricted to the thallus ventral cell layers. (C, D) 'Typical' arbuscules terminal on trunk hyphae in *L. cruciata* (C) and *M. pappeana* (D). Scale bars: (A, B, E, G) 500 μm; (F) 250 μm; (C, D) 10 μm.

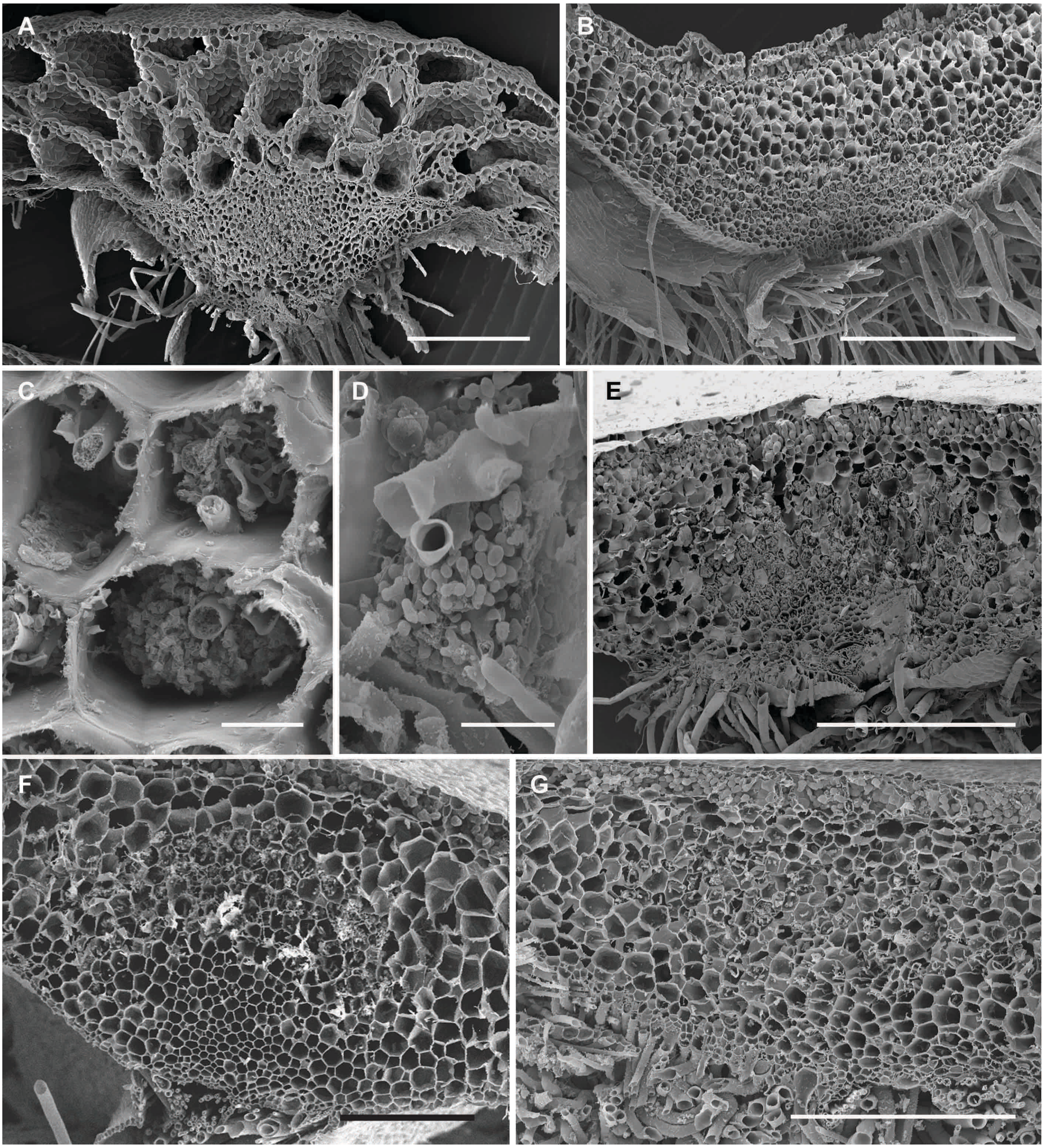
A
B
C
D
E
F
G

Figure S8. Cytology of Glomeromycotina associations in Marchantiopsida (complex thalloid) liverworts, cont. All scanning electron micrographs (except (H) cryo-scanning electron micrograph) of cross sections through thalli (except (G) rhizoid) of (A-C) *Dumortiera hirsuta*; (D) *Asterella australis*; (E) *Plagiochasma rupestris*; (F-I) *Monoclea forsteri*; (J) *Conocephalum conicum*. Fungal colonisation in the region overarching the midrib hyaline strand in *D. hirsuta* (A), the central midrib in *A. australis* (D) and the ventral cell layers in *P. rupestris* (E) and *M. forsteri* (F). In *M. forsteri* the fungal zone extends right across the midribless thallus (F). Fungal structures include vesicles (B, arrowed), coils (C), arbusculated coils (I) and hyphae of varying diameters (J). Fungal entry is invariably via rhizoids (G) while oil bodies idioblasts are always free of fungal structures, shown here in an idioblast in *M. forsteri* (H). Scale bars: (A, D, F) 500 µm; (E) 100 µm; (I, J) 50 µm; (B, C, G) 20 µm; (H) 10 µm.

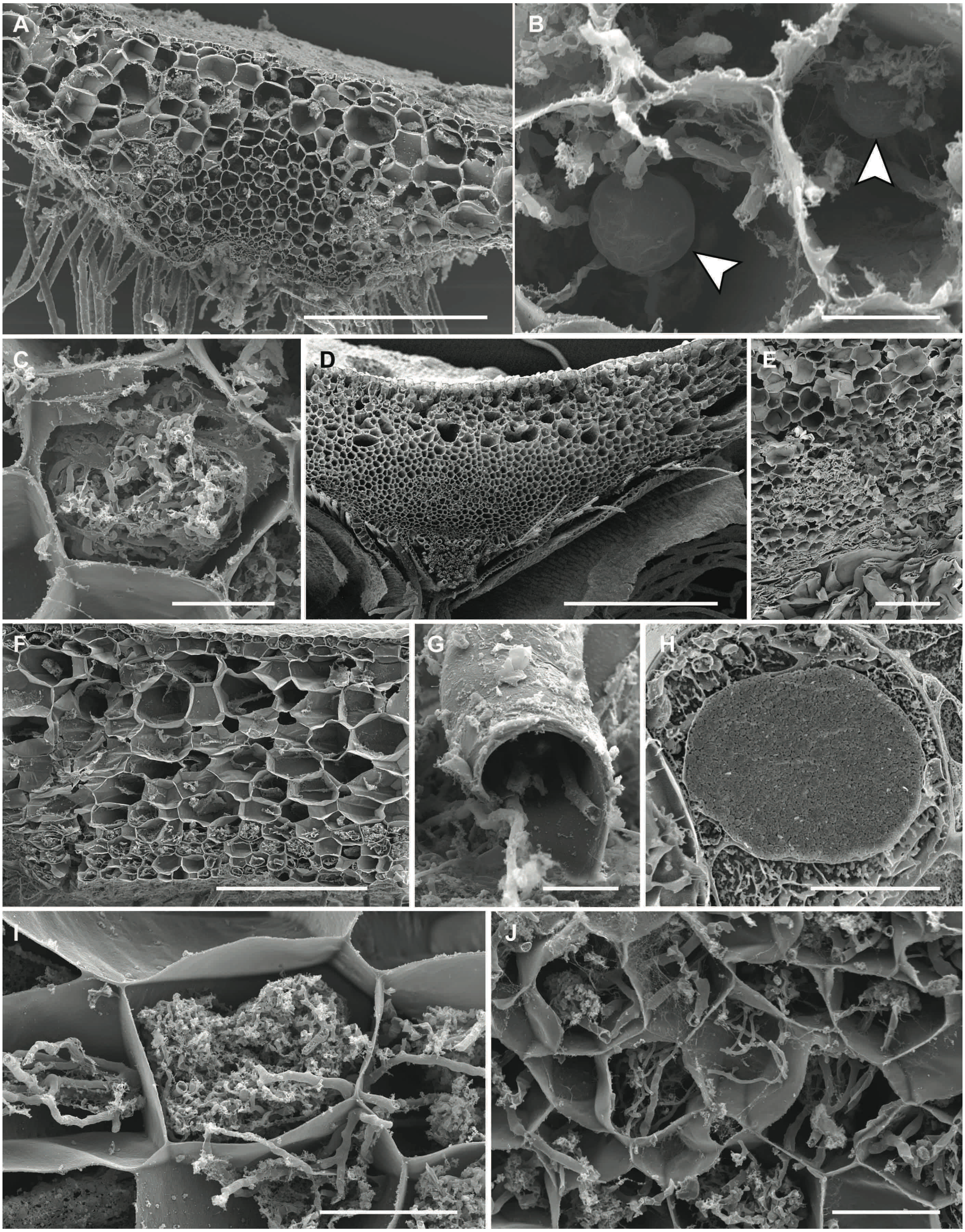
A
B
C
D
E
F
G
H
I
J

Figure S9. Cytology of Glomeromycotina associations in Pelliidae (simple thalloid I) liverworts. Scanning electron micrographs of cross sections through thalli of (A-C) *Pellia epiphylla*; (D) *Sewardiella tuberifera*. Fungal colonisation in Pelliidae liverworts generally occupies the thallus midrib (A) and produces the same range of structures as in Marchantiopsida liverworts, including vesicles of varying sizes, with the largest observed in *P. epiphylla* (B) and the smallest in *S. tuberifera* (D, arrowed), and arbusculated coils (C). Scale bars: (A) 500 μm; (B-D) 20 μm.

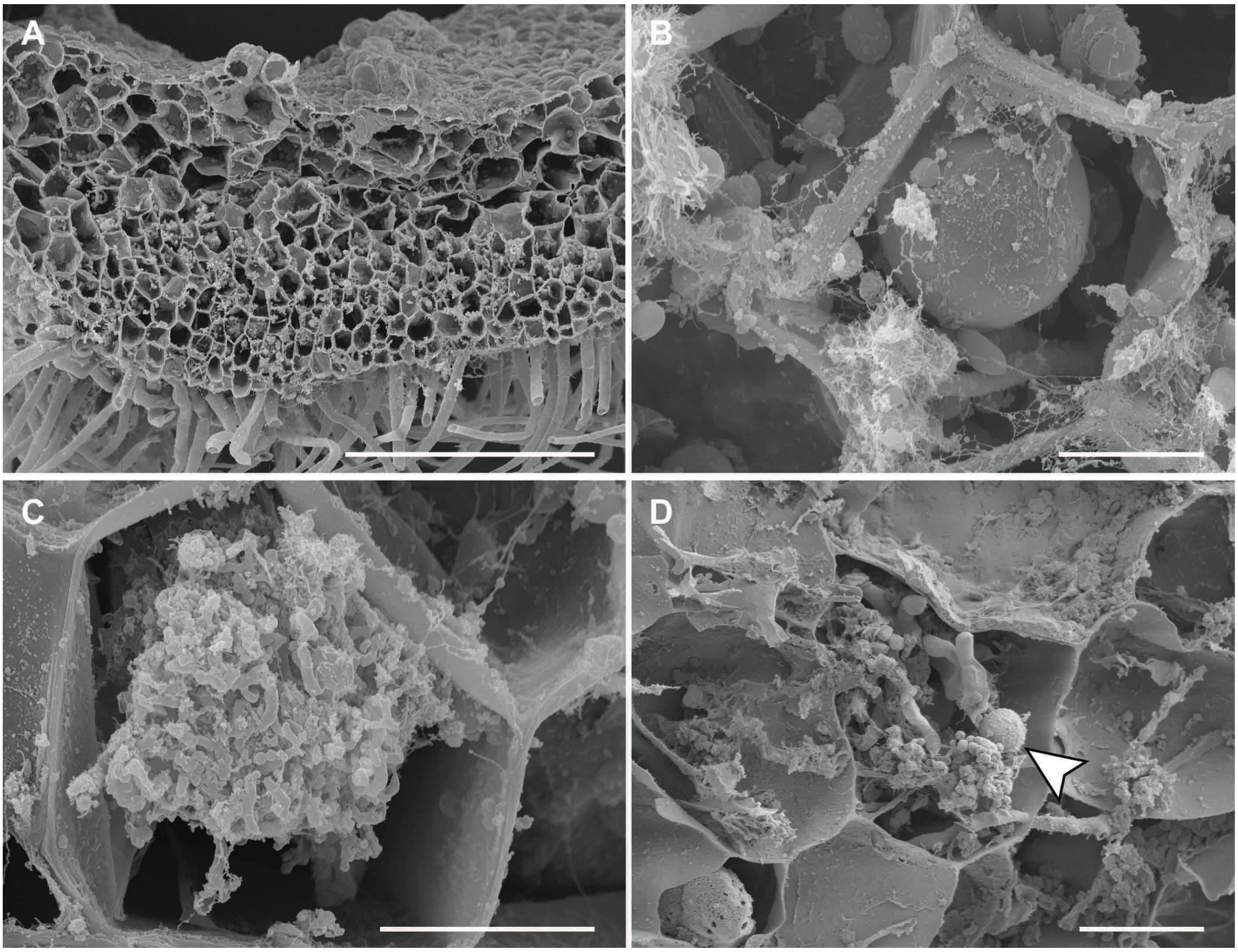
A
B
C
D

Figure S10. Cytology of Glomeromycotina associations in Pelliidae (simple thalloid I) liverworts, cont. Scanning electron micrographs of cross sections through thalli of (A, C-F) *Fossombronia foveolata*; (B) *F. husnotii*; (G) *Allisonia cockaynei*; (H, I) *Pallavicinia xiphoides*; (J) *Symphyogyna brasiliensis*. Fungal structures are randomly distributed in the thallus midrib (A, G) and in *Fossombronia* consist of arbuscules with terminal ramification that are either fine (B) or resembling miniature cauliflowers (C, arrowed) and hyphae of varying dimensions (D), sometimes forming tightly wound coils (E) or with small terminal swellings (F, arrowed). In members of the Pallaviciniaceae fungal colonisation is restricted to the one hemisphere of the subterranean radial axes from which the rhizoids develop (H) and consists of both coils (I) and arbuscules (J). Scale bars: (G) 500 μm; (H) 250 μm; (A, I) 100 μm; (B, D-F, J) 20 μm; (C) 10 μm.

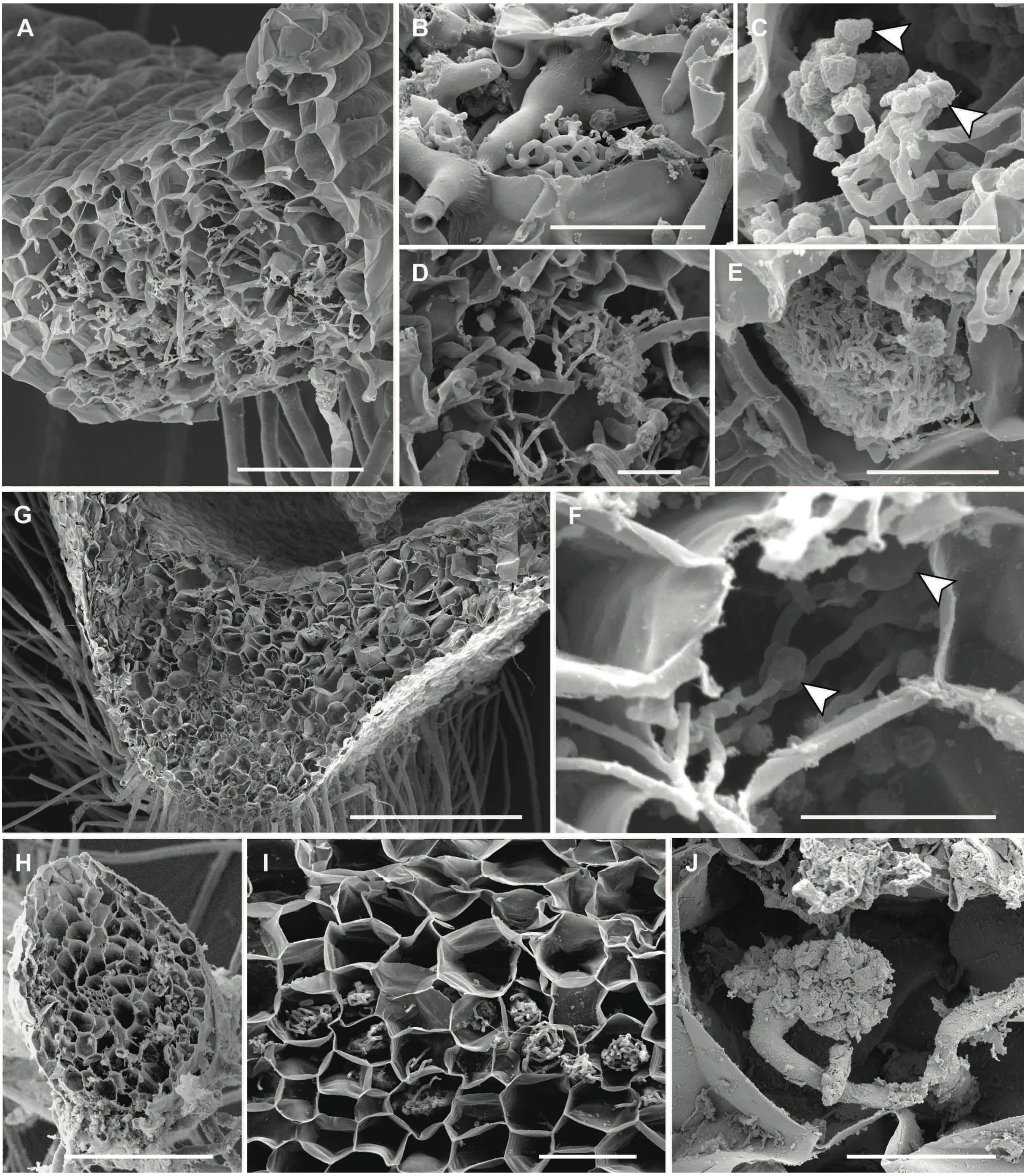
A
B
C
D
E
G
F
H
I
J
