# Supplementary material for: Ancient plants with ancient fungi: liverworts associate with early-diverging arbuscular mycorrhizal fungi
Source: Proc Biol Sci. 2018 Oct 10;285(1888):20181600. doi: 10.1098/rspb.2018.1600 (PMC6191707; doi:10.1098/rspb.2018.1600)
Supplement: Supplementary Tables S1-S6 [file rspb20181600supp2.docx]

**Supplementary material**

**Table S1 to S6**

**Table S1. Liverwort collection details and Glomeromycotina family and epGT detected.** Those samples labeled ‘sp.’ could not be accurately identified to species level. Collectors initials: DJR - David J Read, JGD - Jeffrey G Duckett, KJF - Katie J Field, MIB - Martin I Bidartondo, SP - Silvia Pressel, WRR - William R Rimington. Habitat information for liverwort species can be found in regional floras.

| **Plant species (and authority)** | **Country** | **Latitude** | **Longitude** | **Collector(s)** | **Collection ID** | **Sequencing ID** | **AMF detected?** | **Sequence names** | **epGT identity** | **Glomeromycotina family (or order)** |
| --- | --- | --- | --- | --- | --- | --- | --- | --- | --- | --- |
| *Allisonia cockaynei (Steph.) Schust.* | New Zealand | -42.80000 | 171.55750 | JGD & SP | 2N_2 | 8764, 8765 | No | - | - | - |
| *Allisonia cockaynei (Steph.) Schust.* | New Zealand | -41.22500 | 172.70000 | JGD, KJF & MIB | 3N_51 | WR166 | Yes | WR166-A, WR166-D | epGT_38, epGT_38 | Claroideoglomeraceae, Claroideoglomeraceae |
| *Allisonia cockaynei (Steph.) Schust.* | New Zealand | -41.22500 | 172.70000 | JGD, KJF & MIB | 3N_54 | WR169 | No | - | - | - |
| *Allisonia cockaynei (Steph.) Schust.* | New Zealand | -41.21667 | 172.71667 | JGD, KJF & MIB | 3N_56 | WR171 | No | - | - | - |
| *Allisonia cockaynei (Steph.) Schust.* | New Zealand | -42.80000 | 171.55750 | JGD, KJF & WRR | 4N_148 | WR841 | No | - | - | - |
| *Allisonia cockaynei (Steph.) Schust.* | New Zealand | -42.15000 | 171.77167 | JGD, KJF & WRR | 4N_95 | WR792 | Yes | WR792-D | Singleton | Archaeosporales |
| *Allisonia cockaynei (Steph.) Schust.* | New Zealand | -39.15500 | 175.76667 | JGD, KJF & WRR | 4NN_19 | WR659 | No | - | - | - |
| *Asterella australis (Taylor) Verd.* | New Zealand | -35.60049 | 173.52793 | JGD & SP | 2NN_2 | 8816 | Yes | 8816-A | epGT_31 | Glomeraceae |
| *Asterella australis (Taylor) Verd.* | New Zealand | -41.22500 | 172.76667 | JGD, KJF & MIB | 3N_50 | WR165 | No | - | - | - |
| *Asterella australis (Taylor) Verd.* | New Zealand | -38.60500 | 176.85000 | JGD, KJF & WRR | 4NN_13 | WR655 | No | - | - | - |
| *Asterella australis (Taylor) Verd.* | New Zealand | -39.15500 | 175.76667 | JGD, KJF & WRR | 4NN_22 | WR662 | No | - | - | - |
| *Asterella australis (Taylor) Verd.* | New Zealand | -39.15500 | 175.76667 | JGD, KJF & WRR | 4NN_23 | WR663 | Yes | WR663-D | epGT_31 | Glomeraceae |
| *Asterella australis (Taylor) Verd.* | New Zealand | -39.15500 | 175.76667 | JGD, KJF & WRR | 4NN_24 | WR664 | Yes | WR664-A, WR664-D | epGT_39, epGT_31 | Claroideoglomeraceae, Glomeraceae |
| *Asterella australis (Taylor) Verd.* | New Zealand | -39.26667 | 175.38667 | JGD, KJF & WRR | 4NN_28 | WR668 | Yes | WR668-D | Singleton | Archaeosporales |
| *Asterella australis (Taylor) Verd.* | New Zealand | -39.38333 | 176.43889 | JGD, KJF & WRR | 4NN_45 | WR678 | No | - | - | - |
| *Asterella australis (Taylor) Verd.* | New Zealand | -38.22222 | 176.40000 | JGD, KJF & WRR | 4NN_61 | WR693 | Yes | WR693-A | epGT_31 | Glomeraceae |
| *Asterella bachmannii (Stephani) S.W. Arnell* | Lesotho | -28.76333 | 28.65106 | JGD & SP | L10 | WR75 | Yes | WR75-B | epGT_26 | Glomeraceae |
| *Asterella bachmannii (Stephani) S.W. Arnell* | Lesotho | -29.34617 | 27.85144 | JGD & SP | L30 | WR97 | No | - | - | - |
| *Asterella bachmannii (Stephani) S.W. Arnell* | Lesotho | -28.77033 | 28.63581 | JGD & SP | L4 | WR69 | No | - | - | - |
| *Asterella bachmannii (Stephani) S.W. Arnell* | Lesotho | -29.38443 | 27.92087 | JGD & SP | L48 | WR114 | Yes | WR114-A | epGT_26 | Glomeraceae |
| *Asterella bachmannii (Stephani) S.W. Arnell* | South Africa | -29.27182 | 29.52069 | JGD & SP | SA21 | WR49, WR49.a | No | - | - | - |
| *Asterella bachmannii (Stephani) S.W. Arnell* | South Africa | -28.51008 | 28.63686 | JGD & SP | SA33 | WR61 | Yes | WR61-D | epGT_26 | Glomeraceae |
| *Asterella bachmannii (Stephani) S.W. Arnell* | South Africa | -29.01932 | 29.48285 | JGD & SP | SA5 | WR32, WR32.b | Yes | WR32.B-A, WR32.B-B | epGT_26, epGT_26 | Glomeraceae, Glomeraceae |
| *Asterella bolanderi (Austin) Underw.* | USA | 35.99889 | -121.46814 | JGD | CA16 | 8637 | No | - | - | - |
| *Asterella bolanderi (Austin) Underw.* | USA | 36.53714 | -118.78233 | JGD | CA22 | 8643 | No | - | - | - |
| *Asterella bolanderi (Austin) Underw.* | USA | 36.48533 | -118.56623 | JGD | CA23 | 8644 | Yes | 8644.1.1 | epGT_36 | Claroideoglomeraceae |
| *Asterella californica (Hampe ex Austin) Underw.* | USA | 36.53714 | -118.78233 | JGD | CA20 | 8641 | No | - | - | - |
| *Asterella californica (Hampe ex Austin) Underw.* | USA | 36.53714 | -118.78233 | JGD | CA21 | 8642 | No | - | - | - |
| *Asterella californica (Hampe ex Austin) Underw.* | USA | 36.79609 | -118.59667 | JGD | CA24 | 8645 | No | - | - | - |
| *Asterella californica (Hampe ex Austin) Underw.* | USA | 36.80570 | -118.58365 | JGD | CA26 | 8646 | No | - | - | - |
| *Asterella californica (Hampe ex Austin) Underw.* | USA | 36.82147 | -118.82683 | JGD | CA27 | 8647 | No | - | - | - |
| *Asterella californica (Hampe ex Austin) Underw.* | USA | 36.25653 | -121.78162 | JGD | CA8 | 8629 | No | - | - | - |
| *Asterella drummondii (Taylor) R.M. Schust. ex D.G. Long* | Australia | -37.64317 | 145.54547 | JGD | V_2 | 8824, 8824.b | No | - | - | - |
| *Asterella drummondii (Taylor) R.M. Schust. ex D.G. Long* | New Zealand | -36.96331 | 174.47362 | JGD & SP | 2NN_7 | 8821, 8812.b | No | - | - | - |
| *Asterella grollei D.G. Long* | India | 31.24347 | 77.50050 | JGD & SP | IW100 | wr447 | No | - | - | - |
| *Asterella khasyana (Griff.)* | India | 27.08961 | 88.66194 | JGD & SP | IE108 | wr567 | Yes | WR567-C, WR567-D | epGT_11, epGT30 | Glomeracea, Glomeraceae |
| *Asterella khasyana (Griff.)* | India | 27.08314 | 88.69336 | JGD & SP | IE118 | wr573 | No | - | - | - |
| *Asterella khasyana (Griff.)* | India | 31.11275 | 77.15022 | JGD & SP | IE127 | wr580 | Yes | WR580-A, WR580-B | epGT_34, epGT_34 | Glomerceae, Glomerceae |
| *Asterella khasyana (Griff.)* | India | 27.01175 | 88.22886 | JGD & SP | IE28 | wr504 | No | - | - | - |
| *Asterella khasyana (Griff.)* | India | 26.86794 | 88.28483 | JGD & SP | IE3 | wr483 | No | - | - | - |
| *Asterella khasyana (Griff.)* | India | 27.04003 | 88.21850 | JGD & SP | IE35 | wr510 | Yes | WR510-A | Singleton | Glomeraceae |
| *Asterella khasyana (Griff.)* | India | 31.24347 | 77.50050 | JGD & SP | IW101 | wr448 | No | - | - | - |
| *Asterella khasyana (Griff.)* | India | 31.11372 | 77.15108 | JGD & SP | IW128 | wr474 | Yes | WR474-A, WR474-B | epGT_11, epGT_02 | Glomeraceae, Glomeraceae |
| *Asterella khasyana (Griff.)* | India | 31.11275 | 77.15022 | JGD & SP | IW133 | wr477 | Yes | WR477-A | epGT_11 | Glomeraceae |
| *Asterella khasyana (Griff.)* | India | 31.09756 | 77.18058 | JGD & SP | IW32 | wr372 | No | - | - | - |
| *Asterella khasyana (Griff.)* | India | 31.09756 | 77.18058 | JGD & SP | IW35 | wr375 | No | - | - | - |
| *Asterella khasyana (Griff.)* | India | 31.09803 | 77.18667 | JGD & SP | IW39b | wr380 | No | - | - | - |
| *Asterella khasyana (Griff.)* | India | 31.10508 | 77.14392 | JGD & SP | IW52 | wr391 | No | - | - | - |
| *Asterella khasyana (Griff.)* | India | 31.10778 | 77.25389 | JGD & SP | IW59a | wr401 | No | - | - | - |
| *Asterella khasyana (Griff.)* | India | 31.09931 | 77.26333 | JGD & SP | IW64b | wr409 | Yes | WR409-B | Singleton | Glomeraceae |
| *Asterella khasyana (Griff.)* | India | 31.10778 | 77.25389 | JGD & SP | IW69 | wr413 | No | - | - | - |
| *Asterella khasyana (Griff.)* | India | 31.10778 | 77.25389 | JGD & SP | IW70 | wr414 | Yes | WR414-A, WR414-B | epGT_11, epGT_09 | Glomeraceae, Glomeraceae |
| *Asterella khasyana (Griff.)* | India | 31.11847 | 77.14308 | JGD & SP | IW75 | wr420 | No | - | - | - |
| *Asterella khasyana (Griff.)* | India | 31.11847 | 77.14308 | JGD & SP | IW76a | wr422 | Yes | WR422-D | Singleton | Archaeosporales |
| *Asterella khasyana (Griff.)* | India | 31.09967 | 77.18286 | JGD & SP | IW9 | wr355 | No | - | - | - |
| *Asterella lindenbergiana (Corda) Lindb.* | Switzerland | 46.48255 | 8.38711 | JGD | IS8 | WR626 | No | - | - | - |
| *Asterella muscicola (Stephani) S.W. Arnell* | Lesotho | -28.75772 | 28.53264 | JGD & SP | L2 | WR67 | Yes | WR67-A | epGT_36 | Claroideoglomeraceae |
| *Asterella muscicola (Stephani) S.W. Arnell* | Lesotho | -28.81570 | 28.72020 | JGD & SP | L20.a | WR86 | Yes | WR86-A | epGT_41 | Diversisporaceae |
| *Asterella muscicola (Stephani) S.W. Arnell* | South Africa | -29.00419 | 29.42408 | JGD & SP | SA13 | WR41, WR41.b | No | - | - | - |
| *Asterella muscicola (Stephani) S.W. Arnell* | South Africa | -29.00531 | 29.42344 | JGD & SP | SA15 | WR43, WR43.b | Yes | WR43.B-A | epGT_26 | Glomeraceae |
| *Asterella pringlei Underw.* | Colombia | 7.67903 | -74.02178 | JGD | Co15 | WR1147 | No | - | - | - |
| *Asterella pringlei Underw.* | Colombia | 7.67864 | -74.02344 | JGD | Co19 | WR1149 | No | - | - | - |
| *Asterella pringlei Underw.* | Colombia | 7.69972 | -74.02917 | JGD | Co4 | WR1164 | No | - | - | - |
| *Asterella sp.* | India | 27.06839 | 88.38844 | JGD & SP | IE63 | wr533 | Yes | WR533-A | epGT_11 | Glomeraceae |
| *Asterella sp.* | India | 27.06839 | 88.38844 | JGD & SP | IE63a | wr534 | Yes | WR534-B, WR534-D | epGT_11, epGT_11 | Glomeraceae, Glomeraceae |
| *Asterella sp.* | India | 27.03861 | 88.26361 | JGD & SP | IE77 | wr543 | No | - | - | - |
| *Asterella sp.* | India | 25.78563 | 84.73277 | JGD & SP | IW111 | wr457 | No | - | - | - |
| *Asterella sp.* | India | 31.11275 | 77.15022 | JGD & SP | IW132 | wr476 | Yes | WR476-A, WR476-C | epGT_11, epGT_11 | Glomeraceae, Glomeraceae |
| *Asterella sp.* | India | 31.11275 | 77.15022 | JGD & SP | IW134 | wr478 | Yes | WR478-A | epGT_27 | Glomeraceae |
| *Asterella sp.* | India | 31.09756 | 77.18058 | JGD & SP | IW27 | wr368 | No | - | - | - |
| *Asterella sp.* | India | 31.10508 | 77.14392 | JGD & SP | IW54a | wr394 | No | - | - | - |
| *Asterella sp.* | India | 31.10778 | 77.25389 | JGD & SP | IW66 | wr410 | No | - | - | - |
| *Asterella sp.* | India | 31.09967 | 77.18286 | JGD & SP | IW8 | wr354 | No | - | - | - |
| *Asterella sp.* | India | 31.21769 | 77.43119 | JGD & SP | IW90 | wr436 | No | - | - | - |
| *Asterella tenera (Mitt.) R.M. Schust.* | New Zealand | -43.90833 | 171.25833 | JGD, KJF & MIB | 3N_143 | WR222 | Yes | WR222-A | epGT_38 | Claroideoglomeraceae |
| *Asterella tenera (Mitt.) R.M. Schust.* | New Zealand | -43.89583 | 171.22917 | JGD, KJF & MIB | 3N_149 | WR225 | Yes | WR225-D | epGT_38 | Claroideoglomeraceae |
| *Asterella tenera (Mitt.) R.M. Schust.* | New Zealand | -43.89583 | 171.22500 | JGD, KJF & MIB | 3N_152 | WR228 | Yes | WR228-D | epGT_18 | Glomeraceae |
| *Asterella tenera (Mitt.) R.M. Schust.* | New Zealand | -41.20833 | 172.83889 | JGD, KJF & MIB | 3N_46 | WR163 | Yes | WR163-A | epGT_18 | Glomeraceae |
| *Asterella tenera (Mitt.) R.M. Schust.* | New Zealand | -46.58333 | 169.35000 | JGD, KJF & WRR | 4N_14 | WR715 | No | - | - | - |
| *Asterella tenera (Mitt.) R.M. Schust.* | New Zealand | -41.42417 | 172.10000 | JGD, KJF & WRR | 4N_141 | WR836 | No | - | - | - |
| *Asterella tenera (Mitt.) R.M. Schust.* | New Zealand | -46.50308 | 169.48669 | JGD, KJF & WRR | 4N_25 | WR726 | No | - | - | - |
| *Asterella tenera (Mitt.) R.M. Schust.* | New Zealand | -45.80852 | 170.49072 | JGD, KJF & WRR | 4N_4 | WR705 | Yes | WR705-A | epGT_41 | Diversisporaceae |
| *Asterella tenera (Mitt.) R.M. Schust.* | New Zealand | -38.60500 | 176.85000 | JGD, KJF & WRR | 4NN_14 | WR656 | No | - | - | - |
| *Asterella tenera (Mitt.) R.M. Schust.* | New Zealand | -44.06250 | 169.35833 | JGD, KJF & MIB | 3N_122 | WR210 | Yes | WR210-D | epGT_39 | Claroideoglomeraceae |
| *Asterella wilmsii (Stephani) S.W. Arnell* | Lesotho | -29.12936 | 27.67156 | JGD & SP | L21 | WR88 | No | - | - | - |
| *Asterella wilmsii (Stephani) S.W. Arnell* | South Africa | -29.27283 | 29.51942 | JGD & SP | SA25 | WR53, WR53.b | No | - | - | - |
| *Asterella wilmsii (Stephani) S.W. Arnell* | South Africa | -29.26975 | 29.51867 | JGD & SP | SA28 | WR56 | No | - | - | - |
| *Asterella wilmsii (Stephani) S.W. Arnell* | South Africa | -28.51008 | 28.63686 | JGD & SP | SA34 | WR62 | Yes | WR62-A | epGT_26 | Glomeraceae |
| *Athalamia pinguis Falc.* | India | 31.21769 | 77.43119 | JGD & SP | IW88 | wr434 | Yes | WR434-B | epGT_42 | Diversisporaceae |
| *Calycularia crispula Mitt.* | China | 25.884389 | 110.465333 | SP | CH117a | WR117 | Yes | WR117-H | epGT_14 | Glomeraceae |
| *Calycularia crispula Mitt.* | China | 25.884389 | 110.465333 | SP | CH117b | WR118 | Yes | WR118-A, WR118-C | epGT_53, epGT_51 | Undescribed Archeaosporales A, Undescribed Archeaosporales A |
| *Calycularia crispula Mitt.* | China | 25.39359 | 110.159592 | SP | CH136 | WR20, WR20.b | No | - | - | - |
| *Calycularia crispula Mitt.* | China | 25.14433 | 110.57483 | SP | Ch20 | WR18, WR18.b | No | - | - | - |
| *Calycularia crispula Mitt.* | China | 25.392005 | 110.136049 | SP | CH3 | WR13, WR13.b | Yes | WR13.B-C | epGT_48 | Acaulosporaceae |
| *Calycularia crispula Mitt.* | India | 27.08961 | 88.66194 | JGD & SP | IE106 | wr566 | No | - | - | - |
| *Calycularia crispula Mitt.* | India | 31.09878 | 77.18156 | JGD & SP | IE13 | wr493 | No | - | - | - |
| *Calycularia crispula Mitt.* | India | 27.01175 | 88.22886 | JGD & SP | IE29 | wr505 | Yes | WR505-A, WR505-B, WR505-D | epGT_13, epGT_13, epGT_13 | Glomeraceae, Glomeraceae, Glomeraceae |
| *Calycularia crispula Mitt.* | India | 26.99433 | 88.28578 | JGD & SP | IE48 | wr521 | No | - | - | - |
| *Calycularia crispula Mitt.* | India | 31.24469 | 77.50289 | JGD & SP | IW104 | wr450 | Yes | WR450-B | epGT_51 | Undescribed Archeaosporales A |
| *Clevea hyalina (Sommerf.) Lindb.* | India | 31.09756 | 77.18058 | JGD & SP | IW28 | wr369 | Yes | WR369-C | epGT_42 | Diversisporaceae |
| *Clevea hyalina (Sommerf.) Lindb.* | India | 31.10592 | 77.14097 | JGD & SP | IW43 | wr383 | Yes | WR383-D | Singleton | Diversisporales |
| *Conocephalum conicum (L.) Dumort.* | China | 25.39066 | 110.130461 | SP | CH19 | WR17 | No | - | - | - |
| *Conocephalum conicum (L.) Dumort.* | China | 25.39066 | 110.130461 | SP | CH41 | WR24 | Yes | WR24-A | epGT_31 | Glomerceae |
| *Conocephalum conicum (L.) Dumort.* | England | 51.48207 | -0.29025 | JGD | 8472 | 8472 | Yes | 8472-1, 8472-2 | epGT_11, epGT_11 | Glomeraceae, Glomeraceae |
| *Conocephalum conicum (L.) Dumort.* | England | 50.86947 | 0.62812 | JGD | Cc1 | WR243 | No | - | - | - |
| *Conocephalum conicum (L.) Dumort.* | England | 51.24723 | 0.11961 | JGD & WRR | Con(UK) | WR1171 | No | - | - | - |
| *Conocephalum conicum (L.) Dumort.* | England | 53.85945 | -1.19192 | JGD | T8 | 8608 | Yes | 8608-3 | epGT_39 | Claroideoglomeraceae |
| *Conocephalum conicum (L.) Dumort.* | England | 51.06578 | -0.08905 | JGD | WA1 | WR137, WR137.b | No | - | - | - |
| *Conocephalum conicum (L.) Dumort.* | India | 31.11111 | 77.14764 | JGD & SP | IW124 | wr470 | No | - | - | - |
| *Conocephalum conicum (L.) Dumort.* | India | 31.11847 | 77.14308 | JGD & SP | IW79 | wr425 | No | - | - | - |
| *Conocephalum conicum (L.) Dumort.* | India | 31.09667 | 77.27481 | JGD & SP | IW86 | wr432 | No | - | - | - |
| *Conocephalum conicum (L.) Dumort.* | Italy | 40.70767 | 14.84332 | JGD | Con | 8617 | No | - | - | - |
| *Conocephalum conicum (L.) Dumort.* | Italy | 45.79529 | 8.40823 | JGD | IS13 | WR631 | Yes | WR631-D | epGT_15 | Glomerceae |
| *Conocephalum conicum (L.) Dumort.* | Lesotho | -29.48409 | 27.75147 | JGD & SP | L28 | WR95 | Yes | WR95-B | epGT_26 | Glomerceae |
| *Conocephalum conicum (L.) Dumort.* | Wales | 53.10275 | -3.845714 | JGD & SP | NW4 | WR603 | Yes | WR603-B | epGT_16 | Glomerceae |
| *Conocephalum japonicum (Thunb.)* | India | 27.04494 | 88.26283 | JGD & SP | IE22 | wr500 | Yes | WR500-A, WR500-B, WR500-C | epGT_53, epGT_53, epGT_53 | Undescribed Archeaosporales A, Undescribed Archeaosporales A, Undescribed Archeaosporales A |
| *Conocephalum japonicum (Thunb.)* | India | 27.04003 | 88.21850 | JGD & SP | IE33 | wr508 | Yes | WR508-A, WR508-C | epGT_31, epGT_31 | Glomeraceae, Glomeraceae |
| *Conocephalum salebrosum Szweyk., Buczk. & Odrzyk.* | England | 54.14226 | -2.25334 | JGD | T10 | 8610 | No | - | - | - |
| *Conocephalum salebrosum Szweyk., Buczk. & Odrzyk.* | England | 50.16378 | -5.65459 | JGD | T14o | 8614 | No | - | - | - |
| *Conocephalum salebrosum Szweyk., Buczk. & Odrzyk.* | England | 50.16378 | -5.65459 | JGD | T14y | 8615 | No | - | - | - |
| *Conocephalum salebrosum Szweyk., Buczk. & Odrzyk.* | England | 54.24382 | -2.18939 | JGD | T4 | 8604 | No | - | - | - |
| *Conocephalum salebrosum Szweyk., Buczk. & Odrzyk.* | Iceland | 63.61594 | -19.99230 | SP | IC007 | WR1061, WR1061.a | No | - | - | - |
| *Conocephalum salebrosum Szweyk., Buczk. & Odrzyk.* | Iceland | 63.61594 | -19.99230 | SP | IC019 | WR1056 | Yes | WR1056-A | epGT_11 | Glomerceae |
| *Conocephalum salebrosum Szweyk., Buczk. & Odrzyk.* | Iceland | 63.61594 | -19.99231 | SP | IC030 | WR1064, WR1064.a | No | - | - | - |
| *Conocephalum salebrosum Szweyk., Buczk. & Odrzyk.* | Iceland | 63.61594 | -19.99230 | SP | IC130 | WR1070 | Yes | WR1070-F | epGT_39 | Claroideoglomeraceae |
| *Conocephalum salebrosum Szweyk., Buczk. & Odrzyk.* | Iceland | 63.61594 | -19.99230 | SP | IC198 | WR1083 | Yes | WR1083-A, WR1083-B | epGT_06, epGT_39 | Glomeraceae, Claroideoglomeraceae |
| *Conocephalum salebrosum Szweyk., Buczk. & Odrzyk.* | Ireland | 53.06284 | -6.45480 | JGD | 12H12 | WR1133 | Yes | WR1133-A, WR1133-D | epGT_31, epGT_31 | Glomeraceae, Glomeraceae |
| *Conocephalum salebrosum Szweyk., Buczk. & Odrzyk.* | USA | 44.14843 | -71.68395 | JGD | NH128 | WR307 | Yes | WR307-A | epGT_11 | Glomerceae |
| *Conocephalum salebrosum Szweyk., Buczk. & Odrzyk.* | USA | 44.25943 | -71.25592 | JGD | NH28 | WR271 | Yes | WR271-C | epGT_08 | Glomerceae |
| *Conocephalum salebrosum Szweyk., Buczk. & Odrzyk.* | USA | 44.26072 | -71.25715 | JGD | NH41 | WR273 | Yes | WR273-D | epGT_31 | Glomerceae |
| *Conocephalum salebrosum Szweyk., Buczk. & Odrzyk.* | USA | 44.26330 | -71.29758 | JGD | NH95 | WR304 | Yes | WR304-C | epGT_06 | Glomerceae |
| *Corsinia coriandrina (Spreng.) Lindb.* | Italy | 45.78996 | 8.42169 | JGD | 14cc | WR941 | No | - | - | - |
| *Corsinia coriandrina (Spreng.) Lindb.* | Italy | 45.79529 | 8.40823 | JGD | IS5 | WR623 | No | - | - | - |
| *Corsinia coriandrina (Spreng.) Lindb.* | Italy | 45.79466 | 8.40980 | JGD | IS6 | WR624 | No | - | - | - |
| *Corsinia coriandrina (Spreng.) Lindb.* | Italy | 37.65773 | 14.89341 | JGD | sc19 | WR890 | No | - | - | - |
| *Cryptomitrium himalayense Kashyap* | India | 31.09619 | 77.17853 | JGD & SP | IW116 | wr462 | No | - | - | - |
| *Cryptomitrium himalayense Kashyap* | India | 31.09619 | 77.17853 | JGD & SP | IW118 | wr464 | No | - | - | - |
| *Cryptomitrium himalayense Kashyap* | India | 31.09967 | 77.18286 | JGD & SP | IW7 | wr353 | No | - | - | - |
| *Cryptomitrium himalayense Kashyap* | India | 31.11481 | 77.24853 | JGD & SP | IW81 | wr427 | No | - | - | - |
| *Cryptomitrium oreades Perold* | Lesotho | -28.77408 | 28.67419 | JGD & SP | L19.a | WR84, WR84.b | No | - | - | - |
| *Cryptomitrium oreades Perold* | Lesotho | -28.77408 | 28.67419 | JGD & SP | L19.b | WR85, WR85.b | No | - | - | - |
| *Cyathodium aureonitens (Griff.) Mitt.* | India | 31.11847 | 77.14308 | JGD & SP | IW77 | wr423 | No | - | - | - |
| *Cyathodium cavernarum Kunze* | Brazil | -24.636489 | -48.404389 | SP | BR8 | WR910 | No | - | - | - |
| *Cyathodium cavernarum Kunze* | Colombia | 7.67903 | -74.02178 | JGD | Co18 | WR1148 | No | - | - | - |
| *Cyathodium sp.* | India | 27.04003 | 88.21850 | JGD & SP | IE40 | wr514 | No | - | - | - |
| *Cyathodium sp.* | India | 27.03803 | 88.26317 | JGD & SP | IE7 | wr487 | No | - | - | - |
| *Cyathodium tuberosum Kashyap* | India | 31.09967 | 77.18286 | JGD & SP | IW2 | wr348 | No | - | - | - |
| *Cyathodium tuberosum Kashyap* | India | 31.09756 | 77.18058 | JGD & SP | IW26 | wr367 | No | - | - | - |
| *Dumortiera hirsuta (Sw.) Nees* | Brazil | -22.95892 | -43.27717 | SP | 2BR3 | WR1158 | No | - | - | - |
| *Dumortiera hirsuta (Sw.) Nees* | Brazil | -20.163247 | -51.207393 | SP | BR1 | WR902 | Yes | WR902-A, WR902-B | epGT_53, Singleton | Undescribed Archeaosporales A, Glomeraceae |
| *Dumortiera hirsuta (Sw.) Nees* | Brazil | -24.636489 | -48.404389 | SP | BR10 | WR912 | Yes | WR912-B | epGT_19 | Glomeraceae |
| *Dumortiera hirsuta (Sw.) Nees* | Brazil | -24.735617 | -48.335817 | SP | BR16 | WR918 | Yes | WR918-D | epGT_24 | Glomeraceae |
| *Dumortiera hirsuta (Sw.) Nees* | Brazil | -24.636489 | -48.404389 | SP | BR7a | WR908 | No | - | - | - |
| *Dumortiera hirsuta (Sw.) Nees* | Brazil | -24.636489 | -48.404389 | SP | BR7b | WR909 | No | - | - | - |
| *Dumortiera hirsuta (Sw.) Nees* | China | 25.39066 | 110.130461 | SP | CH126 | WR9, WR119 | Yes | WR9-D, WR119-A, WR119-B | epGT_53, epGT_53, epGT_15 | Undescribed Archeaosporales A, Undescribed Archeaosporales A, Glomeraceae |
| *Dumortiera hirsuta (Sw.) Nees* | China | 25.392005 | 110.136049 | SP | CH2 | WR12 | Yes | WR12-A | epGT_47 | Acaulosporaceae |
| *Dumortiera hirsuta (Sw.) Nees* | Colombia | 7.67903 | -74.02178 | JGD | Co14 | WR1146 | No | - | - | - |
| *Dumortiera hirsuta (Sw.) Nees* | Colombia | 7.68090 | -74.01162 | JGD | Co21 | WR1150 | No | - | - | - |
| *Dumortiera hirsuta (Sw.) Nees* | England | 50.86947 | 0.62812 | JGD | Dh1 | WR238 | Yes | WR238-D | epGT_43 | Diversisporaceae |
| *Dumortiera hirsuta (Sw.) Nees* | England | 50.86887 | 0.62923 | JGD | Dh2 | WR239 | No | - | - | - |
| *Dumortiera hirsuta (Sw.) Nees* | England | 50.86704 | 0.63055 | JGD | Dh3 | WR240 | No | - | - | - |
| *Dumortiera hirsuta (Sw.) Nees* | India | 27.04494 | 88.26283 | JGD & SP | IE10 | wr490 | No | - | - | - |
| *Dumortiera hirsuta (Sw.) Nees* | India | 27.08794 | 88.69850 | JGD & SP | IE114 | wr570 | Yes | WR570-C | epGT_50 | Undescribed Archeaosporales A |
| *Dumortiera hirsuta (Sw.) Nees* | India | 27.10478 | 88.63897 | JGD & SP | IE94 | wr554 | No | - | - | - |
| *Dumortiera hirsuta (Sw.) Nees* | India | 31.11111 | 77.14764 | JGD & SP | IW123 | wr469 | No | - | - | - |
| *Dumortiera hirsuta (Sw.) Nees* | India | 31.11847 | 77.14308 | JGD & SP | IW78 | wr424 | No | - | - | - |
| *Dumortiera hirsuta (Sw.) Nees* | Malaysia | -6.74133 | 107.00748 | SP | MA_84.1 | 8880, 8880.b | Yes | 8880.B-A | epGT_11 | Glomeraceae |
| *Dumortiera hirsuta (Sw.) Nees* | Malaysia | -6.74133 | 107.00748 | SP | MA_84.2 | 8881 | Yes | 8881-A | Singleton | Paraglomerales |
| *Dumortiera hirsuta (Sw.) Nees* | Malaysia | 4.47353 | 101.38228 | SP | MA_59.1 | 8864, 8864.b | No | - | - | - |
| *Dumortiera hirsuta (Sw.) Nees* | Malaysia | 4.47353 | 101.38228 | SP | MA_59.2 | 8865 | Yes | 8865-A | epGT_39 | Claroideoglomeraceae |
| *Dumortiera hirsuta (Sw.) Nees* | Vietnam | 11.641481 | 107.742325 | SP | VI_5a | WR127 | Yes | WR127-A | epGT_39 | Claroideoglomeraceae |
| *Dumortiera hirsuta (Sw.) Nees* | Vietnam | 11.641481 | 107.742325 | SP | VI_5b | WR128 | Yes | WR128-B, WR128-E | epGT_20, epGT_20 | Glomeraceae, Glomeraceae |
| *Dumortiera hirsuta (Sw.) Nees* | Vietnam | 11.641481 | 107.742325 | SP | VI_5c | WR129 | Yes | WR129-E | epGT_35 | Glomeraceae |
| *Dumortiera hirsuta (Sw.) Nees* | Indonesia | -8.27201 | 115.15108 | DJR | BA1(Bali) | WR253 | No | - | - | - |
| *Dumortiera hirsuta (Sw.) Nees* | Indonesia | -8.27791 | 115.15130 | DJR | BA11 | WR264 | No | - | - | - |
| *Dumortiera hirsuta (Sw.) Nees* | Indonesia | -8.27648 | 115.14914 | DJR | BA6 | WR258 | No | - | - | - |
| *Dumortiera hirsuta (Sw.) Nees* | South Africa | -29.00715 | 29.42321 | JGD & SP | SA19 | WR47 | Yes | WR47-B | epGT_31 | Glomeraceae |
| *Dumortiera hirsuta (Sw.) Nees* | South Africa | -29.00531 | 29.42344 | JGD & SP | SA12.a | WR39, WR39.a | No | - | - | - |
| *Dumortiera hirsuta (Sw.) Nees* | South Africa | -29.00531 | 29.42344 | JGD & SP | SA12.b | WR40, WR40.b | No | - | - | - |
| *Fossombronia angulifolia Perold* | Lesotho | -29.29667 | 27.69897 | JGD & SP | L35 | WR102 | Yes | WR102-D | epGT_29 | Glomeraceae |
| *Fossombronia australis Mitt.* | New Zealand | -42.73889 | 171.80000 | JGD & SP | 2N_88 | 8796 | Yes | 8796-D | epGT_16 | Glomeraceae |
| *Fossombronia australis Mitt.* | New Zealand | -42.57500 | 171.45833 | JGD, KJF & MIB | 3N_105 | WR201 | No | - | - | - |
| *Fossombronia australis Mitt.* | New Zealand | -41.10833 | 172.96667 | JGD, KJF & MIB | 3N_22 | WR154 | No | - | - | - |
| *Fossombronia australis Mitt.* | New Zealand | -41.15556 | 172.91667 | JGD, KJF & MIB | 3N_24 | WR155 | Yes | WR155-A, WR155-B | epGT_53, epGT_39 | Undescribed Archeaosporales A, Claroideoglomeraceae |
| *Fossombronia australis Mitt.* | New Zealand | -42.30556 | 172.11667 | JGD, KJF & MIB | 3N_94 | WR192 | No | - | - | - |
| *Fossombronia australis Mitt.* | New Zealand | -43.13333 | 171.77000 | JGD, KJF & WRR | 4N_158 | WR849 | No | - | - | - |
| *Fossombronia australis Mitt.* | New Zealand | -44.75750 | 167.95750 | JGD, KJF & WRR | 4N_55 | WR754 | No | - | - | - |
| *Fossombronia australis Mitt.* | New Zealand | -42.30750 | 172.10750 | JGD, KJF & WRR | 4N_99 | WR796 | Yes | WR796-B | epGT_38 | Claroideoglomeraceae |
| *Fossombronia australis Mitt.* | New Zealand | -38.59083 | 176.81667 | JGD, KJF & WRR | 4NN_10 | WR652 | Yes | WR652-A | epGT_25 | Glomeraceae |
| *Fossombronia australis Mitt.* | New Zealand | -39.38333 | 176.43889 | JGD, KJF & WRR | 4NN_44 | WR677 | No | - | - | - |
| *Fossombronia caespitiformis Corb.* | Italy | 37.85135 | 15.28997 | JGD | sc13 | WR886 | No | - | - | - |
| *Fossombronia caespitiformis Corb.* | Italy | 37.29200 | 13.58153 | JGD | sc25 | WR893 | Yes | WR893-B | epGT_54 | Undescribed Archeaosporales B |
| *Fossombronia caespitiformis Corb.* | Italy | 37.29200 | 13.58153 | JGD | sc31 | WR874 | No | - | - | - |
| *Fossombronia caespitiformis Corb.* | Italy | 37.28875 | 13.60044 | JGD | sc34b | WR898 | No | - | - | - |
| *Fossombronia caespitiformis Corb.* | Italy | 37.28875 | 13.60044 | JGD | sc36 | WR900 | Yes | - | - | - |
| *Fossombronia caespitiformis Corb.* | Italy | 37.28875 | 13.60044 | JGD | sc37 | WR877 | No | - | - | - |
| *Fossombronia caespitiformis Corb.* | Italy | 37.28875 | 13.60044 | JGD | sc39 | WR878 | No | - | - | - |
| *Fossombronia caespitiformis Corb.* | Italy | 37.28875 | 13.60044 | JGD | sc41 | WR879 | Yes | WR879-D | epGT_56 | Undescribed Archeaosporales B |
| *Fossombronia echinata Macvicar* | Italy | 40.66189 | 14.80657 | JGD | F1 | 8616 | No | - | - | - |
| *Fossombronia echinata Macvicar* | Spain | 41.36719 | 2.15283 | JGD | BA1 | WR1166 | Yes | WR1166-C | epGT_56 | Undescribed Archeaosporales B |
| *Fossombronia echinata Macvicar* | Spain | 41.36719 | 2.15283 | JGD | BA2 | WR1167 | No | - | - | - |
| *Fossombronia foveolata Lindb.* | England | 51.15950 | 0.71128 | JGD & WRR | Th1 | TS60 | No | - | - | - |
| *Fossombronia foveolata Lindb.* | England | 51.15950 | 0.71128 | JGD & WRR | Th2 | TS61 | No | - | - | - |
| *Fossombronia foveolata Lindb.* | England | 51.15950 | 0.71128 | JGD & WRR | Th3 | TS62 | No | - | - | - |
| *Fossombronia foveolata Lindb.* | England | 51.15950 | 0.71128 | JGD & WRR | Th4 | TS63 | Yes | TS63.2 | epGT_53 | Undescribed Archeaosporales A |
| *Fossombronia foveolata Lindb.* | England | 51.15950 | 0.71128 | JGD & WRR | Th5 | TS64 | No | - | - | - |
| *Fossombronia foveolata Lindb.* | Iceland | 64.02548 | -21.21254 | SP | IC088 | WR1067, WR1067.a | No | - | - | - |
| *Fossombronia foveolata Lindb.* | Iceland | 64.02568 | -21.21242 | SP | IC090 | WR956, WR956.a | No | - | - | - |
| *Fossombronia foveolata Lindb.* | South Africa | -29.00715 | 29.42321 | JGD & SP | SA20 | WR48 | Yes | WR48-A, WR48-B | Singleton, epGT_14 | Glomeraceae, Glomeraceae |
| *Fossombronia foveolata Lindb.* | South Africa | -29.00681 | 29.42283 | JGD & SP | SA6 | WR33 | No | - | - | - |
| *Fossombronia husnotii Corb.* | Ascension Island | -7.95031 | -14.34674 | JGD & SP | 2AI184 | WR1105 | No | - | - | - |
| *Fossombronia husnotii Corb.* | Ascension Island | -7.95347 | -14.34647 | JGD & SP | 2AI201 | WR1109 | No | - | - | - |
| *Fossombronia husnotii Corb.* | Ascension Island | -7.95527 | -14.34565 | JGD & SP | 2AI5 | WR948 | No | - | - | - |
| *Fossombronia husnotii Corb.* | Ascension Island | -7.95527 | -14.34565 | JGD & SP | 2AI6 | WR949 | Yes | WR949-B | epGT_23 | Glomeraceae |
| *Fossombronia husnotii Corb.* | Ascension Island | -7.94912 | -14.35190 | JGD & SP | AI11 | TS8 | Yes | TS8N.4 | epGT_07 | Glomeraceae |
| *Fossombronia husnotii Corb.* | Ascension Island | -7.94851 | -14.33731 | JGD & SP | AI15 | TS12 | Yes | TS12b.2 | epGT_41 | Diversisporaceae |
| *Fossombronia husnotii Corb.* | Ascension Island | -7.94916 | -14.33666 | JGD & SP | AI20 | TS17 | No | - | - | - |
| *Fossombronia husnotii Corb.* | Ascension Island | -7.95046 | -14.35047 | JGD | F61.A | 8555 | Yes | 8555-3 | epGT_31 | Glomeraceae |
| *Fossombronia husnotii Corb.* | Ascension Island | -7.95047 | -14.34989 | JGD | F61.B | 8556 | Yes | 8556-4 | epGT_07 | Glomeraceae |
| *Fossombronia hyalorhiza Perold* | South Africa | -29.27269 | 29.52067 | JGD & SP | SA24 | WR52 | No | - | - | - |
| *Fossombronia hyalorhiza Perold* | South Africa | -29.27900 | 29.51686 | JGD & SP | SA26 | WR54 | Yes | WR54-A | epGT_58 | Archeosporaceae |
| *Fossombronia incurva Lindb.* | Iceland | 64.31394 | -20.30596 | SP | IC051 | WR958, WR958.a | No | - | - | - |
| *Fossombronia incurva Lindb.* | Iceland | 64.31394 | -20.30594 | SP | IC015 | WR1057, WR1057.a | No | - | - | - |
| *Fossombronia incurva Lindb.* | Iceland | 64.31394 | -20.30596 | SP | IC052 | WR959, WR959.a | No | - | - | - |
| *Fossombronia incurva Lindb.* | Iceland | 64.31347 | -20.30150 | SP | IC055 | WR963 | Yes | WR963-E, WR963-H | epGT_27, epGT_58 | Glomeraceae, Archaeosporaceae |
| *Fossombronia indica Steph.* | Ascension Island | -7.95117 | -14.34665 | JGD & SP | AI183 | TS46 | Yes | TS46.4 | Singleton | Claroideoglomeraceae |
| *Fossombronia indica Steph.* | Ascension Island | -7.95117 | -14.34665 | JGD & SP | AI185 | TS48 | Yes | TS48.2 | Singleton | Glomeraceae |
| *Fossombronia indica Steph.* | Ascension Island | -7.95099 | -14.34360 | JGD & SP | AI215 | TS55 | Yes | TS55b.1, TS55b.2 | epGT_22, epGT_44 | Glomeraceae, Gigasporaceae |
| *Fossombronia indica Steph.* | Ascension Island | -7.96211 | -14.34446 | JGD & SP | AI216 | TS56 | No | - | - | - |
| *Fossombronia indica Steph.* | Ascension Island | -7.94916 | -14.33666 | JGD & SP | AI22 | TS19 | Yes | TS19.1 | epGT_23 | Glomeraceae |
| *Fossombronia indica Steph.* | Ascension Island | -7.94916 | -14.33666 | JGD & SP | AI23 | TS20 | Yes | TS20.4 | epGT_24 | Glomeraceae |
| *Fossombronia indica Steph.* | Ascension Island | -7.95051 | -14.33554 | JGD & SP | AI26 | TS23 | Yes | TS23n.2 | epGT_07 | Glomeraceae |
| *Fossombronia indica Steph.* | Ascension Island | -7.95183 | -14.35385 | JGD & SP | AI36 | TS31 | Yes | TS31.2 | epGT_14 | Glomeraceae |
| *Fossombronia indica Steph.* | Ascension Island | -7.95884 | -14.34445 | JGD & SP | AI96 | TS41 | Yes | TS41b.4 | epGT_14 | Glomeraceae |
| *Fossombronia indica Steph.* | Ascension Island | -7.95043 | -14.35015 | JGD | F66 | 8561 | Yes | 8561-3 | epGT_29 | Glomeraceae |
| *Fossombronia indica Steph.* | Ascension Island | -7.95031 | -14.34674 | JGD & SP | 2AI184A | WR1106 | No | - | - | - |
| *Fossombronia kashyapii S.C.Srivast. & Udar* | India | 31.11372 | 77.15108 | JGD & SP | IW125 | wr471 | No | - | - | - |
| *Fossombronia kashyapii S.C.Srivast. & Udar* | India | 31.09756 | 77.18058 | JGD & SP | IW36 | wr376 | Yes | WR376-A, WR376-C | epGT_42, epGT_49 | Diversisporaceae, Acaulosporaceae |
| *Fossombronia kashyapii S.C.Srivast. & Udar* | India | 31.09931 | 77.26333 | JGD & SP | IW60a | wr403 | No | - | - | - |
| *Fossombronia kashyapii S.C.Srivast. & Udar* | India | 31.09667 | 77.27481 | JGD & SP | IW84 | wr430 | No | - | - | - |
| *Fossombronia maritima (Paton) Paton* | England | 50.66803 | -4.76013 | JGD | FM | 8489 | Yes | 8489 | Singleton | Glomeraceae |
| *Fossombronia porphyrorhiza (Nees) Prosk.* | Brazil | -22.96946 | -43.22479 | SP | 2BR9 | WR1162 | No | - | - | - |
| *Fossombronia pusilla (L.) Nees* | India | 27.08961 | 88.66194 | JGD & SP | IE109 | wr568 | No | - | - | - |
| *Fossombronia pusilla (L.) Nees* | India | 27.04494 | 88.26283 | JGD & SP | IE19 | wr499 | Yes | WR499-B | epGT_15 | Glomeraceae |
| *Fossombronia pusilla (L.) Nees* | India | 27.06839 | 88.38844 | JGD & SP | IE58 | wr530 | No | - | - | - |
| *Fossombronia pusilla (L.) Nees* | India | 31.24469 | 77.50289 | JGD & SP | IW108a | wr453 | Yes | WR453-C | epGT_42 | Diversisporaceae |
| *Fossombronia pusilla (L.) Nees* | India | 31.09967 | 77.18286 | JGD & SP | IW4 | wr350 | Yes | WR350-A, WR350-B | epGT_52, epGT_52 | Undescribed Archeaosporales A, Undescribed Archeaosporales A |
| *Fossombronia pusilla (L.) Nees* | India | 31.10778 | 77.25389 | JGD & SP | IW59 | wr400 | No | - | - | - |
| *Fossombronia pusilla (L.) Nees* | India | 31.09931 | 77.26333 | JGD & SP | IW61 | wr404 | No | - | - | - |
| *Fossombronia pusilla (L.) Nees* | India | 31.24347 | 77.50050 | JGD & SP | IW95a | wr441 | No | - | - | - |
| *Fossombronia pusilla (L.) Nees* | India | 31.24347 | 77.50050 | JGD & SP | IW95b | wr442 | Yes | WR442-D | epGT_58 | Archeosporaceae |
| *Fossombronia pusilla (L.) Nees* | Italy | 37.28875 | 13.60044 | JGD | sc34a | WR897 | Yes | WR897-F | epGT_11 | Glomeraceae |
| *Fossombronia pusilla (L.) Nees* | New Zealand | -42.37000 | 171.40000 | JGD & SP | 2N_50 | 8782 | Yes | 8782-A | epGT_49 | Acaulosporaceae |
| *Fossombronia pusilla (L.) Nees* | New Zealand | -41.73889 | 171.47500 | JGD & SP | 2N_68 | 8786 | Yes | 8786-B | epGT_57 | Archeosporaceae |
| *Fossombronia pusilla (L.) Nees* | New Zealand | -36.96331 | 174.47362 | JGD & SP | 2NN_6 | 8820 | No | - | - | - |
| *Fossombronia pusilla (L.) Nees* | New Zealand | -41.09167 | 172.97917 | JGD, KJF & MIB | 3N_17 | WR153 | No | - | - | - |
| *Fossombronia pusilla (L.) Nees* | New Zealand | -41.83804 | 171.50458 | JGD, KJF & MIB | 3N_68 | WR180 | No | - | - | - |
| *Fossombronia pusilla (L.) Nees* | New Zealand | -42.72917 | 170.95833 | JGD, KJF & MIB | 3N_93 | WR191 | Yes | WR191-C | epGT_28 | Glomeraceae |
| *Fossombronia pusilla (L.) Nees* | New Zealand | -41.15500 | 172.17417 | JGD, KJF & WRR | 4N_125 | WR820 | Yes | WR820-A | epGT_02 | Glomeraceae |
| *Fossombronia pusilla (L.) Nees* | New Zealand | -41.25000 | 172.10500 | JGD, KJF & WRR | 4N_130 | WR825 | No | - | - | - |
| *Fossombronia pusilla (L.) Nees* | New Zealand | -41.25333 | 172.18333 | JGD, KJF & WRR | 4N_135 | WR830 | No | - | - | - |
| *Fossombronia pusilla (L.) Nees* | New Zealand | -46.59083 | 169.35500 | JGD, KJF & WRR | 4N_19 | WR720 | Yes | WR720-B | epGT_17 | Glomeraceae |
| *Fossombronia pusilla (L.) Nees* | New Zealand | -46.26667 | 167.90500 | JGD, KJF & WRR | 4N_31 | WR733 | Yes | WR733-B | Singleton | Glomeraceae |
| *Fossombronia pusilla (L.) Nees* | New Zealand | -45.80852 | 170.49072 | JGD, KJF & WRR | 4N_5 | WR706 | Yes | WR706-A | epGT_45 | Acaulosporaceae |
| *Fossombronia pusilla (L.) Nees* | New Zealand | -43.45875 | 170.02059 | JGD, KJF & WRR | 4N_78 | WR775 | Yes | WR775-B | epGT_39 | Claroideoglomeraceae |
| *Fossombronia pusilla (L.) Nees* | New Zealand | -42.15000 | 171.77167 | JGD, KJF & WRR | 4N_98 | WR795 | Yes | WR795-B, WR795-D | epGT_16, epGT_17 | Glomeraceae, Glomeraceae |
| *Fossombronia pusilla (L.) Nees* | New Zealand | -42.30750 | 172.10750 | JGD, KJF & WRR | 4N_99a | WR797 | No | - | - | - |
| *Fossombronia pusilla (L.) Nees* | New Zealand | -38.65500 | 176.08833 | JGD, KJF & WRR | 4NN_2 | WR644 | No | - | - | - |
| *Fossombronia pusilla (L.) Nees* | New Zealand | -39.29083 | 175.35500 | JGD, KJF & WRR | 4NN_32 | WR670 | Yes | WR670-C | epGT_39 | Claroideoglomeraceae |
| *Fossombronia pusilla (L.) Nees* | New Zealand | -38.22222 | 176.40000 | JGD, KJF & WRR | 4NN_59 | WR691 | No | - | - | - |
| *Fossombronia pusilla (L.) Nees* | New Zealand | -38.78333 | 176.23333 | JGD, KJF & WRR | 4NN_65 | WR697 | No | - | - | - |
| *Fossombronia pusilla (L.) Nees* | USA | **37.89555** | -122.24225 | JGD | CA1 | 8622 | No | - | - | - |
| *Fossombronia pusilla (L.) Nees* | USA | 35.99714 | -121.47842 | JGD | CA10 | 8631 | No | - | - | - |
| *Fossombronia pusilla (L.) Nees* | USA | 36.53714 | -118.78233 | JGD | CA19 | 8640 | No | - | - | - |
| *Fossombronia pusilla (L.) Nees* | USA | 37.87297 | -122.23714 | JGD | CA5 | 8626 | No | - | - | - |
| *Fossombronia pusilla (L.) Nees* | Wales | 52.89717 | -3.91958 | JGD & SP | NW3 | WR602 | Yes | WR602-D | epGT_53 | Undescribed Archeaosporales A |
| *Fossombronia reticulata Steph.* | New Zealand | -39.38333 | 176.43889 | JGD, KJF & WRR | 4NN_51 | WR683 | No | - | - | - |
| *Fossombronia sp.* | Australia | -37.64317 | 145.54547 | JGD | V_1 | 8823, 8823.b | No | - | - | - |
| *Fossombronia sp.* | Australia | -38.63669 | 143.89144 | JGD | V_7 | 8828 | Yes | 8828-D | epGT_33 | Glomeraceae |
| *Fossombronia sp.* | Australia | -37.64317 | 145.54547 | JGD | V4 | 8825 | Yes | 8825-C | epGT_42 | Diversisporaceae |
| *Fossombronia sp.* | China | 25.08303 | 110.28797 | SP | CH13 | WR16 | Yes | WR16-C | epGT_31 | Glomeraceae |
| *Fossombronia sp.* | Lesotho | -28.92669 | 28.75517 | JGD & SP | L15 | WR80 | No | - | - | - |
| *Fossombronia sp.* | Lesotho | -29.66478 | 27.79692 | JGD & SP | L24 | WR91 | No | - | - | - |
| *Fossombronia sp.* | Lesotho | -29.11014 | 28.47772 | JGD & SP | L37 | WR104 | No | - | - | - |
| *Fossombronia sp.* | Lesotho | -29.11993 | 28.43698 | JGD & SP | L39 | WR106 | Yes | WR106-A, WR106-D | epGT_49, epGT_58 | Acaulosporaceae, Archaeosporaceae |
| *Fossombronia sp.* | Lesotho | -28.76403 | 28.64683 | JGD & SP | L8 | WR73 | No | - | - | - |
| *Fossombronia wondraczekii (Corda) Dumort.* | Iceland | 64.31394 | -20.30594 | SP | IC008 | WR1060 | No | - | - | - |
| *Fossombronia wondraczekii (Corda) Dumort.* | Iceland | 64.67018 | -21.25922 | SP | IC010 | WR1059, WR1059.a | Yes | WR1059.a-D | epGT_31 | Glomeraceae |
| *Fossombronia wondraczekii (Corda) Dumort.* | Iceland | 64.02567 | -21.21242 | SP | IC029 | WR1065 | Yes | WR1065-B | Singleton | Diversisporales |
| *Fossombronia wondraczekii (Corda) Dumort.* | Iceland | 64.02797 | -21.21464 | SP | IC056 | WR962 | Yes | WR962-C | Singleton | Glomeraceae |
| *Fossombronia wondraczekii (Corda) Dumort.* | Iceland | 64.23622 | -21.04461 | SP | IC084 | WR1066, WR1066.a | No | - | - | - |
| *Fossombronia wondraczekii (Corda) Dumort.* | Iceland | 64.86800 | -23.86221 | SP | IC092 | WR964, WR964.a | Yes | WR964.a-C | epGT_40 | Claroideoglomeraceae |
| *Fossombronia wondraczekii (Corda) Dumort.* | Iceland | 64.31343 | -20.29799 | SP | IC112 | WR1068, WR1068.a | No | - | - | - |
| *Fossombronia wondraczekii (Corda) Dumort.* | Iceland | 64.31394 | -20.30594 | SP | IC138 | WR1073 | No | - | - | - |
| *Fossombronia wondraczekii (Corda) Dumort.* | Wales | 53.117461 | -4.027806 | JGD & SP | NW2 | WR601 | Yes | WR601-C, WR601-E | epGT_56, epGT-56 | Undescribed Archeaosporales B, Undescribed Archeaosporales B |
| *Haplomitrium dentatum (D. Kumar & Udar) J.J. Engel* | India | 27.08444 | 88.66975 | JGD & SP | IE100 | wr559 | No | - | - | - |
| *Haplomitrium dentatum (D. Kumar & Udar) J.J. Engel* | India | 27.00372 | 88.26317 | JGD & SP | IE49 | wr523 | No | - | - | - |
| *Haplomitrium gibbsiae (Stephani) R.M. Schust.* | New Zealand | -44.06250 | 169.35833 | JGD, KJF & MIB | 3N_118 | WR206 | No | - | - | - |
| *Haplomitrium gibbsiae (Stephani) R.M. Schust.* | New Zealand | -42.37500 | 172.39583 | JGD, KJF & MIB | 3N_97 | WR194 | No | - | - | - |
| *Haplomitrium gibbsiae (Stephani) R.M. Schust.* | New Zealand | -42.30750 | 172.10750 | JGD, KJF & WRR | 4N_100 | WR798 | No | - | - | - |
| *Haplomitrium gibbsiae (Stephani) R.M. Schust.* | New Zealand | -42.29083 | 172.08833 | JGD, KJF & WRR | 4N_103 | WR801 | No | - | - | - |
| *Haplomitrium gibbsiae (Stephani) R.M. Schust.* | New Zealand | -46.59083 | 169.35500 | JGD, KJF & WRR | 4N_18 | WR719 | No | - | - | - |
| *Haplomitrium gibbsiae (Stephani) R.M. Schust.* | New Zealand | -46.26667 | 167.90500 | JGD, KJF & WRR | 4N_34 | WR736 | No | - | - | - |
| *Haplomitrium gibbsiae (Stephani) R.M. Schust.* | New Zealand | -44.10750 | 169.35000 | JGD, KJF & WRR | 4N_66 | WR764 | No | - | - | - |
| *Haplomitrium gibbsiae (Stephani) R.M. Schust.* | New Zealand | -39.15500 | 175.76667 | JGD, KJF & WRR | 4NN_26 | WR666 | No | - | - | - |
| *Haplomitrium hookeri (Sm.) Nees* | USA | 44.26289 | -71.29989 | JGD | NH70 | WR287 | No | - | - | - |
| *Haplomitrium hookeri (Sm.) Nees* | USA | 44.26286 | -71.29966 | JGD | NH71 | WR288 | No | - | - | - |
| *Haplomitrium hookeri (Sm.) Nees* | Wales | 53.117461 | -4.027806 | JGD & SP | NW1 | WR600 | No | - | - | - |
| *Haplomitrium mnioides (Lindb.) R.M. Schust.* | China | 25.14433 | 110.57483 | SP | CH16a | WR21 | No | - | - | - |
| *Haplomitrium mnioides (Lindb.) R.M. Schust.* | China | 25.39359 | 110.159592 | SP | CH16b | WR22, WR22.b | No | - | - | - |
| *Haplomitrium ovalifolium Schust.* | New Zealand | -39.30750 | 175.50750 | JGD, KJF & WRR | 4NN_34 | WR671 | No | - | - | - |
| *Hymenophyton flabellatum (Labill.) Dumort.* | New Zealand | -43.98333 | 168.60833 | JGD, KJF & MIB | 3N_133 | WR217 | No | - | - | - |
| *Hymenophyton flabellatum (Labill.) Dumort.* | New Zealand | -43.90833 | 171.25833 | JGD, KJF & MIB | 3N_147 | WR224 | No | - | - | - |
| *Hymenophyton flabellatum (Labill.) Dumort.* | New Zealand | -43.89583 | 171.22917 | JGD, KJF & MIB | 3N_151 | WR227 | No | - | - | - |
| *Hymenophyton flabellatum (Labill.) Dumort.* | New Zealand | -43.89583 | 171.22500 | JGD, KJF & MIB | 3N_153 | WR229 | Yes | WR229-D | epGT_45 | Acaulosporaceae |
| *Jensenia crassifrons (Steph.) S.Schuette & Stotler* | Falkland Islands | -51.69234 | -57.77730 | JGD | F49 | 8549 | Yes | 8549-1 | epGT_04 | Glomeraceae |
| *Lunularia cruciata (L.) Dumort. ex Lindb.* | Australia | -38.66611 | 143.86203 | JGD | V_5 | 8826 | Yes | 8826-B | epGT_37 | Claroideoglomeraceae |
| *Lunularia cruciata (L.) Dumort. ex Lindb.* | Brazil | -22.96013 | -43.27534 | SP | 2BR1 | WR1156 | No | - | - | - |
| *Lunularia cruciata (L.) Dumort. ex Lindb.* | Brazil | -24.735617 | -48.335817 | SP | BR15a | WR917 | No | - | - | - |
| *Lunularia cruciata (L.) Dumort. ex Lindb.* | Colombia | 4.60507 | -74.05797 | JGD | Co25 | WR1153 | No | - | - | - |
| *Lunularia cruciata (L.) Dumort. ex Lindb.* | England | 51.22298 | -3.78422 | JGD | Lc1 | WR244 | No | - | - | - |
| *Lunularia cruciata (L.) Dumort. ex Lindb.* | England | 51.13796 | -3.75664 | JGD | Lc2 | WR245 | Yes | WR245-A, WR245-D | Singleton, epGT_15 | Glomeraceae, Glomeraceae |
| *Lunularia cruciata (L.) Dumort. ex Lindb.* | England | 51.49595 | -0.17843 | JGD | Lc3 | WR246 | No | - | - | - |
| *Lunularia cruciata (L.) Dumort. ex Lindb.* | England | 51.15048 | -0.71916 | JGD & WRR | Lun | WR1170 | No | - | - | - |
| *Lunularia cruciata (L.) Dumort. ex Lindb.* | England | 51.57634 | -0.14336 | JGD & WRR | Lun2 | WR1172 | No | - | - | - |
| *Lunularia cruciata (L.) Dumort. ex Lindb.* | England | 51.15887 | -0.69046 | JGD | T1 | 8601 | No | - | - | - |
| *Lunularia cruciata (L.) Dumort. ex Lindb.* | Germany | 52.45255 | 13.30826 | JGD | 12H5 | WR1121 | No | - | - | - |
| *Lunularia cruciata (L.) Dumort. ex Lindb.* | India | 27.04494 | 88.26283 | JGD & SP | IE11 | wr491 | No | - | - | - |
| *Lunularia cruciata (L.) Dumort. ex Lindb.* | Indonesia | -8.27201 | 115.15108 | DJR | BA3(Bali) | WR255 | Yes | WR255-A | epGT_31 | Glomeraceae |
| *Lunularia cruciata (L.) Dumort. ex Lindb.* | Italy | 37.29200 | 13.58153 | JGD | sc26 | WR894 | No | - | - | - |
| *Lunularia cruciata (L.) Dumort. ex Lindb.* | Italy | 37.28875 | 13.60044 | JGD | sc42 | WR901 | No | - | - | - |
| *Lunularia cruciata (L.) Dumort. ex Lindb.* | Italy | 37.07391 | 15.28536 | JGD | sc5 | WR882 | Yes | WR882-C | epGT_37 | Claroideoglomeraceae |
| *Lunularia cruciata (L.) Dumort. ex Lindb.* | New Zealand | -41.74914 | 171.46600 | JGD & SP | 2N_69 | 8787 | Yes | 8787-B | epGT_45 | Acaulosporaceae |
| *Lunularia cruciata (L.) Dumort. ex Lindb.* | New Zealand | -36.96179 | 174.51439 | JGD & SP | 2NN_4 | 8818 | Yes | 8818-C | epGT_39 | Claroideoglomeraceae |
| *Lunularia cruciata (L.) Dumort. ex Lindb.* | New Zealand | -44.08889 | 169.35000 | JGD, KJF & MIB | 3N_123 | WR211 | No | - | - | - |
| *Lunularia cruciata (L.) Dumort. ex Lindb.* | New Zealand | -40.00833 | 171.17500 | JGD, KJF & MIB | 3N_157 | WR231 | No | - | - | - |
| *Lunularia cruciata (L.) Dumort. ex Lindb.* | New Zealand | -40.00833 | 171.17500 | JGD, KJF & MIB | 3N_158 | WR232 | No | - | - | - |
| *Lunularia cruciata (L.) Dumort. ex Lindb.* | New Zealand | -41.09167 | 172.97917 | JGD, KJF & MIB | 3N_16 | WR152 | Yes | WR152-A | epGT_08 | Glomeraceae |
| *Lunularia cruciata (L.) Dumort. ex Lindb.* | New Zealand | -43.60556 | 172.65000 | JGD, KJF & MIB | 3N_160 | WR234 | No | - | - | - |
| *Lunularia cruciata (L.) Dumort. ex Lindb.* | New Zealand | -41.87500 | 171.49167 | JGD, KJF & MIB | 3N_63 | WR176 | Yes | WR176-A | epGT_38 | Claroideoglomeraceae |
| *Lunularia cruciata (L.) Dumort. ex Lindb.* | New Zealand | -42.25556 | 173.83333 | JGD, KJF & MIB | 3N_7 | WR144, WR144.b | No | - | - | - |
| *Lunularia cruciata (L.) Dumort. ex Lindb.* | New Zealand | -42.72917 | 170.95833 | JGD, KJF & MIB | 3N_92 | WR190 | Yes | WR190-A, WR190-B | epGT_28, epGT_40 | Glomeraceae, Claroideoglomeraceae |
| *Lunularia cruciata (L.) Dumort. ex Lindb.* | New Zealand | -41.15500 | 172.17417 | JGD, KJF & WRR | 4N_123 | WR818 | No | - | - | - |
| *Lunularia cruciata (L.) Dumort. ex Lindb.* | New Zealand | -41.25000 | 172.10500 | JGD, KJF & WRR | 4N_133 | WR828 | Yes | WR828-B | epGT_31 | Glomeraceae |
| *Lunularia cruciata (L.) Dumort. ex Lindb.* | New Zealand | -38.65500 | 176.08833 | JGD, KJF & WRR | 4NN_1 | WR643 | No | - | - | - |
| *Lunularia cruciata (L.) Dumort. ex Lindb.* | New Zealand | -38.22222 | 176.40000 | JGD, KJF & WRR | 4NN_57 | WR689 | Yes | WR689-A | epGT_39 | Claroideoglomeraceae |
| *Lunularia cruciata (L.) Dumort. ex Lindb.* | New Zealand | -38.78333 | 176.23333 | JGD, KJF & WRR | 4NN_66 | WR698 | No | - | - | - |
| *Lunularia cruciata (L.) Dumort. ex Lindb.* | New Zealand | -38.60500 | 176.73333 | JGD, KJF & WRR | 4NN_9 | WR651 | No | - | - | - |
| *Lunularia cruciata (L.) Dumort. ex Lindb.* | South Africa | -33.98816 | 18.43269 | JGD & SP | SA35 | WR63 | Yes | WR63-D | epGT_31 | Glomeraceae |
| *Lunularia cruciata (L.) Dumort. ex Lindb.* | Spain | 41.36733 | 2.15294 | JGD | BA3 | WR1168 | Yes | WR1168-C | epGT_37 | Claroideoglomeraceae |
| *Lunularia cruciata (L.) Dumort. ex Lindb.* | Spain | 41.36733 | 2.15294 | JGD | BA4 | WR1169 | No | - | - | - |
| *Lunularia cruciata (L.) Dumort. ex Lindb.* | USA | 37.87297 | -122.23714 | JGD | CA4 | 8625 | Yes | 8625.1.4 | Singleton | Glomeraceae |
| *Mannia gracilis (F.Weber) Schill & D.G.Long* | Italy | 45.79529 | 8.40823 | JGD | IS7 | WR625 | No | - | - | - |
| *Mannia sp.* | China | 25.392005 | 110.136049 | SP | CH9 | WR15, WR15.b | No | - | - | - |
| *Mannia sp.* | India | 31.09619 | 77.17853 | JGD & SP | IE122 | wr577 | Yes | WR577-A | epGT_16 | Glomeraceae |
| *Mannia sp.* | India | 31.24469 | 77.50289 | JGD & SP | IW105 | wr451 | No | - | - | - |
| *Mannia sp.* | India | 31.24469 | 77.50289 | JGD & SP | IW108 | wr452 | No | - | - | - |
| *Marchantia berteroana Lehm. & Lindenb.* | Brazil | -24.735617 | -48.335817 | SP | BR13 | WR915 | Yes | WR915-A | epGT_53 | Undescribed Archeaosporales A |
| *Marchantia berteroana Lehm. & Lindenb.* | Chile | -54.93851 | -67.62371 | JGD | CH8 | WR1165 | No | - | - | - |
| *Marchantia berteroana Lehm. & Lindenb.* | Falkland Islands | -51.67496 | -58.11217 | JGD | F22 | 8542 | No | - | - | - |
| *Marchantia berteroana Lehm. & Lindenb.* | Falkland Islands | -51.40244 | -59.22743 | JGD | F33 | 8545 | No | - | - | - |
| *Marchantia berteroana Lehm. & Lindenb.* | Falkland Islands | -51.89599 | -58.39153 | JGD | F47 | 8547 | Yes | 8547-3 | epGT_05 | Glomeraceae |
| *Marchantia berteroana Lehm. & Lindenb.* | Indonesia | -8.27791 | 115.15130 | DJR | BA10 | WR263 | No | - | - | - |
| *Marchantia berteroana Lehm. & Lindenb.* | New Zealand | -44.82417 | 168.10750 | JGD, KJF & WRR | 4N_50 | WR749 | No | - | - | - |
| *Marchantia berteroana Lehm. & Lindenb.* | New Zealand | -44.75750 | 167.95750 | JGD, KJF & WRR | 4N_54 | WR753 | Yes | WR753-A | epGT_25 | Glomeraceae |
| *Marchantia berteroana Lehm. & Lindenb.* | New Zealand | -38.78333 | 176.23333 | JGD, KJF & WRR | 4NN_67 | WR699 | Yes | WR699-A | epGT_11 | Glomeraceae |
| *Marchantia chenopoda L.* | Brazil | -22.96861 | -43.22182 | SP | 2BR10 | WR1163 | Yes | WR1163-A, WR1163-C | epGT_03, epGT_03 | Glomeraceae, Glomeraceae |
| *Marchantia chenopoda L.* | Brazil | -22.96581 | -43.22728 | SP | 2BR7 | WR1160 | No | - | - | - |
| *Marchantia chenopoda L.* | Colombia | 7.67813 | -74.02133 | JGD | Co13 | WR1145 | Yes | WR1145-C | epGT_40 | Claroideoglomeraceae |
| *Marchantia chenopoda L.* | Colombia | 4.60507 | -74.05797 | JGD | Co26 | WR1154 | No | - | - | - |
| *Marchantia chenopoda L.* | Colombia | 4.60507 | -74.05797 | JGD | Co27 | WR1155 | No | - | - | - |
| *Marchantia chenopoda L.* | Colombia | 7.69972 | -74.02917 | JGD | Co5 | WR1141 | Yes | WR1141-A, WR1141-C, WR1141-D | epGT_57, epGT_30, epGT_31 | Archeosporaceae, Glomeraceae, Glomeraceae |
| *Marchantia chenopoda L.* | Colombia | 7.69972 | -74.02917 | JGD | Co6 | WR1142 | Yes | WR1142-A, WR1142-B, WR1142-C | epGT_10, epGT_24, epGT_26 | Glomeraceae, Glomeraceae, Glomeraceae |
| *Marchantia debilis K.I. Goebel* | South Africa | -29.01932 | 29.48285 | JGD & SP | SA4.a | WR30 | Yes | WR30-A | epGT_31 | Glomeraceae |
| *Marchantia debilis K.I. Goebel* | Lesotho | -29.42350 | 27.91636 | JGD & SP | L44 | WR110, WR110.b | Yes | WR110.B-A, WR110.B-D | epGT_26, epGT_26 | Glomeraceae, Glomeraceae |
| *Marchantia debilis K.I. Goebel* | South Africa | -29.27345 | 29.52104 | JGD & SP | SA23 | WR51 | Yes | WR51-D | epGT_26 | Glomeraceae |
| *Marchantia debilis K.I. Goebel* | South Africa | -29.01932 | 29.48285 | JGD & SP | SA4.b | WR31 | Yes | WR31-A | epGT_31 | Glomeraceae |
| *Marchantia foliacea Mitt.* | New Zealand | -44.06250 | 169.35833 | JGD, KJF & MIB | 3N_120 | WR208 | Yes | WR208-C | epGT_04 | Glomeraceae |
| *Marchantia foliacea Mitt.* | New Zealand | -43.98333 | 168.60000 | JGD, KJF & MIB | 3N_130 | WR214 | Yes | WR214-A | Singleton | Glomeraceae |
| *Marchantia foliacea Mitt.* | New Zealand | -41.09167 | 172.97917 | JGD, KJF & MIB | 3N_15 | WR151, WR151.b | No | - | - | - |
| *Marchantia foliacea Mitt.* | New Zealand | -43.89583 | 171.22917 | JGD, KJF & MIB | 3N_150 | WR226 | Yes | WR226-D | epGT_38 | Claroideoglomeraceae |
| *Marchantia foliacea Mitt.* | New Zealand | -43.75000 | 171.74417 | JGD, KJF & MIB | 3N_159 | WR233 | No | - | - | - |
| *Marchantia foliacea Mitt.* | New Zealand | -41.20000 | 172.80833 | JGD, KJF & MIB | 3N_34 | WR158 | Yes | WR158-A | epGT_18 | Glomeraceae |
| *Marchantia foliacea Mitt.* | New Zealand | -41.21667 | 172.71667 | JGD, KJF & MIB | 3N_57 | WR172 | No | - | - | - |
| *Marchantia foliacea Mitt.* | New Zealand | -42.25556 | 173.83333 | JGD, KJF & MIB | 3N_8 | WR145, WR145.b | Yes | WR145.B-A | epGT_18 | Glomeraceae |
| *Marchantia foliacea Mitt.* | New Zealand | -43.45556 | 170.45556 | JGD, KJF & MIB | 3N_83 | WR185 | Yes | WR185-D | epGT_25 | Glomeraceae |
| *Marchantia foliacea Mitt.* | New Zealand | -43.40000 | 170.18333 | JGD, KJF & MIB | 3N_86 | WR187 | Yes | WR187-D | epGT_25 | Glomeraceae |
| *Marchantia foliacea Mitt.* | New Zealand | -42.80000 | 171.55750 | JGD, KJF & WRR | 4N_151 | WR844 | No | - | - | - |
| *Marchantia foliacea Mitt.* | New Zealand | -38.60500 | 176.85000 | JGD, KJF & WRR | 4NN_16 | WR658 | Yes | WR658-D | Singleton | Claroideoglomeraceae |
| *Marchantia foliacea Mitt.* | New Zealand | -39.15500 | 175.76667 | JGD, KJF & WRR | 4NN_25 | WR665 | No | - | - | - |
| *Marchantia foliacea Mitt.* | New Zealand | -39.26667 | 175.38667 | JGD, KJF & WRR | 4NN_31 | WR669 | Yes | WR669-B, WR669-C | epGT_18, Singleton | Glomeraceae, Glomeraceae |
| *Marchantia foliacea Mitt.* | New Zealand | -39.38333 | 176.43889 | JGD, KJF & WRR | 4NN_46 | WR679 | No | - | - | - |
| *Marchantia paleacea Bertol.* | India | 31.10592 | 77.14097 | JGD & SP | IW44 | wr384 | Yes | WR384-A, WR384-D | epGT_31, epGT_58 | Glomeraceae, Archaesporaceae |
| *Marchantia paleacea Bertol.* | India | 31.09667 | 77.27481 | JGD & SP | IW87 | wr433 | Yes | WR433-C, WR433-D | epGT_43, epGT_43 | Diversisporaceae, Diversisporaceae |
| *Marchantia paleacea Bertol.* | Indonesia | -8.27648 | 115.14914 | DJR | BA8a | WR260 | No | - | - | - |
| *Marchantia paleacea Bertol.* | Indonesia | -8.27648 | 115.14914 | DJR | BA8b | WR261 | No | - | - | - |
| *Marchantia paleacea Bertol.* | Italy | 40.71405 | 14.84720 | JGD | Mar | 8618 | No | - | - | - |
| *Marchantia paleacea Bertol.* | Malaysia | 4.47353 | 101.38228 | SP | MA_60 | 8866, 8866.b | No | - | - | - |
| *Marchantia papillata Raddi* | India | 31.09878 | 77.18156 | JGD & SP | IE12 | wr492 | Yes | WR492-A, WR492-B, WR492-C | epGT_35, epGT_11, epGT_09 | Glomeraceae, Glomeraceae, Glomeraceae |
| *Marchantia papillata Raddi* | Malaysia | 4.47353 | 101.38228 | SP | MA_61 | 8867 | Yes | 8867-B | epGT_14 | Glomeraceae |
| *Marchantia papillata Raddi* | Malaysia | 3.72493 | 101.71435 | SP | MA_80 | 8877, 8877.b | Yes | 8877-A, 8877.B-A, 8877.B-E | epGT_09, epGT_19, epGT_19 | Glomeraceae, Glomeraceae, Glomeraceae |
| *Marchantia papillata Raddi* | Malaysia | 3.72493 | 101.71435 | SP | MA_81 | 8878 | Yes | 8878-A | epGT_03 | Glomeraceae |
| *Marchantia pappeana Lehm.* | Ascension Island | -7.95138 | -14.34529 | JGD & SP | AI186 | TS49 | Yes | TS49.7 | epGT_29 | Glomeraceae |
| *Marchantia pappeana Lehm.* | Ascension Island | -7.95138 | -14.34529 | JGD & SP | AI187 | TS50 | Yes | TS50.1 | epGT_31 | Glomeraceae |
| *Marchantia pappeana Lehm.* | South Africa | -29.01571 | 29.48223 | JGD & SP | SA1.a | WR25 | Yes | WR25-A, WR25-C | epGT_31, epGT_26 | Glomeraceae, Glomeraceae |
| *Marchantia pappeana Lehm.* | South Africa | -33.98902 | 18.43201 | JGD & SP | SA36 | WR64, WR64.b | No | - | - | - |
| *Marchantia pappeana Lehm.* | South Africa | -29.27182 | 29.52069 | JGD & SP | SA22 | WR50 | Yes | WR50-D | epGT_26 | Glomeraceae |
| *Marchantia pappeana Lehm.* | South Africa | -29.01571 | 29.48223 | JGD & SP | SA1.b | WR26 | Yes | WR26-A, WR26-B | epGT_26, epGT_31 | Glomeraceae, Glomeraceae |
| *Marchantia polymorpha L.* | New Zealand | -41.25000 | 172.10500 | JGD, KJF & WRR | 4N_131 | WR826 | No | - | - | - |
| *Marchantia polymorpha subsp. montivagans Bischl. & Boissel.* | France | 45.67485 | 6.88097 | JGD | 12H3 | WR1119 | No | - | - | - |
| *Marchantia polymorpha subsp. montivagans Bischl. & Boissel.* | France | 44.69049 | 6.97570 | JGD | IS4 | WR622 | No | - | - | - |
| *Marchantia polymorpha subsp. montivagans Bischl. & Boissel.* | Iceland | 63.61594 | -19.99230 | SP | IC131 | WR1071, WR1071.a | No | - | - | - |
| *Marchantia polymorpha subsp. montivagans Bischl. & Boissel.* | Iceland | 63.40449 | -19.04686 | SP | IC183 | WR1079, WR1079.a | No | - | - | - |
| *Marchantia polymorpha subsp. montivagans Bischl. & Boissel.* | Iceland | 63.40449 | -19.04686 | SP | IC184 | WR1080, WR1080.a | No | - | - | - |
| *Marchantia polymorpha subsp. montivagans Bischl. & Boissel.* | Iceland | 63.53358 | -19.36061 | SP | IC192 | WR1082, WR1082.a | No | - | - | - |
| *Marchantia polymorpha subsp. montivagans Bischl. & Boissel.* | Iceland | 63.61594 | -19.99230 | SP | IC199 | WR966, WR966.a | No | - | - | - |
| *Marchantia polymorpha subsp. montivagans Bischl. & Boissel.* | Italy | 46.48255 | 8.38711 | JGD | IS11 | WR629 | No | - | - | - |
| *Marchantia polymorpha subsp. ruderalis Bischl. & Boissel.* | Italy | 45.79529 | 8.40823 | JGD | IS12 | WR630 | No | - | - | - |
| *Marchantia polymorpha subsp. ruderalis Bischl. & Boissel.* | Italy | 45.79529 | 8.40823 | JGD | IS14 | WR632 | No | - | - | - |
| *Marchantia sp.* | Indonesia | -8.27648 | 115.14914 | DJR | BA7 | WR259 | Yes | WR259-A | Singleton | Glomeraceae |
| *Moerckia blyttii (Mørch) Brockm.* | Switzerland | 46.48014 | 8.40008 | JGD | IS10 | WR628 | Yes | WR628-B, WR628-D | Singleton, epGT_13 | Diversisporales, Glomeraceae |
| *Monoclea forsteri Hook.* | New Zealand | -41.86250 | 171.50000 | JGD & SP | 2N_101 | 8801, 8801.b | No | - | - | - |
| *Monoclea forsteri Hook.* | New Zealand | -42.89583 | 171.12500 | JGD & SP | 2N_31 | 8773 | Yes | 8773-C | epGT_25 | Glomeraceae |
| *Monoclea forsteri Hook.* | New Zealand | -43.49167 | 170.04583 | JGD & SP | 2N_35 | 8776 | No | - | - | - |
| *Monoclea forsteri Hook.* | New Zealand | -42.35333 | 171.35500 | JGD & SP | 2N_48 | 8781 | No | - | - | - |
| *Monoclea forsteri Hook.* | New Zealand | -42.09194 | 171.33775 | JGD & SP | 2N_73 | 8789 | Yes | 8789-B | epGT_36 | Claroideoglomeraceae |
| *Monoclea forsteri Hook.* | New Zealand | -36.96179 | 174.51439 | JGD & SP | 2NN_3 | 8817, 8817.b | No | - | - | - |
| *Monoclea forsteri Hook.* | New Zealand | -42.37500 | 172.39583 | JGD, KJF & MIB | 3N_104 | WR200 | No | - | - | - |
| *Monoclea forsteri Hook.* | New Zealand | -44.94167 | 169.26667 | JGD, KJF & MIB | 3N_127 | WR213 | No | - | - | - |
| *Monoclea forsteri Hook.* | New Zealand | -43.98333 | 168.60833 | JGD, KJF & MIB | 3N_132 | WR216 | No | - | - | - |
| *Monoclea forsteri Hook.* | New Zealand | -43.90833 | 171.25833 | JGD, KJF & MIB | 3N_140 | WR220 | No | - | - | - |
| *Monoclea forsteri Hook.* | New Zealand | -43.90833 | 171.25833 | JGD, KJF & MIB | 3N_144 | WR223 | No | - | - | - |
| *Monoclea forsteri Hook.* | New Zealand | -43.89583 | 171.22500 | JGD, KJF & MIB | 3N_155 | WR230 | Yes | WR230-A, WR230-B, WR230-C, WR230-D | epGT_53, Singleton, Singleton, epGT_53 | Undescribed Archeaosporales A, Glomeraceae, Diversisporales, Undescribed Archeaosporales A |
| *Monoclea forsteri Hook.* | New Zealand | -42.24815 | 173.82602 | JGD, KJF & MIB | 3N_4 | WR142, WR142.b | No | - | - | - |
| *Monoclea forsteri Hook.* | New Zealand | -41.20556 | 172.83333 | JGD, KJF & MIB | 3N_44 | WR161 | Yes | WR161-A | epGT_35 | Glomeraceae |
| *Monoclea forsteri Hook.* | New Zealand | -41.87500 | 171.49167 | JGD, KJF & MIB | 3N_64 | WR177 | No | - | - | - |
| *Monoclea forsteri Hook.* | New Zealand | -45.80852 | 170.49072 | JGD, KJF & WRR | 4N_1 | WR702 | No | - | - | - |
| *Monoclea forsteri Hook.* | New Zealand | -42.18833 | 171.93833 | JGD, KJF & WRR | 4N_104 | WR802 | Yes | WR802-B | epGT_18 | Glomeraceae |
| *Monoclea forsteri Hook.* | New Zealand | -41.13833 | 172.18333 | JGD, KJF & WRR | 4N_109 | WR806 | No | - | - | - |
| *Monoclea forsteri Hook.* | New Zealand | -41.15000 | 172.19083 | JGD, KJF & WRR | 4N_117 | WR812 | No | - | - | - |
| *Monoclea forsteri Hook.* | New Zealand | -41.00000 | 172.16667 | JGD, KJF & WRR | 4N_120 | WR815 | No | - | - | - |
| *Monoclea forsteri Hook.* | New Zealand | -41.10500 | 172.10000 | JGD, KJF & WRR | 4N_127 | WR822 | No | - | - | - |
| *Monoclea forsteri Hook.* | New Zealand | -46.58333 | 169.35000 | JGD, KJF & WRR | 4N_13 | WR714 | No | - | - | - |
| *Monoclea forsteri Hook.* | New Zealand | -41.25333 | 172.18333 | JGD, KJF & WRR | 4N_137 | WR832 | No | - | - | - |
| *Monoclea forsteri Hook.* | New Zealand | -41.42417 | 172.10000 | JGD, KJF & WRR | 4N_139 | WR834 | No | - | - | - |
| *Monoclea forsteri Hook.* | New Zealand | -46.52000 | 169.55500 | JGD, KJF & WRR | 4N_27 | WR728 | No | - | - | - |
| *Monoclea forsteri Hook.* | New Zealand | -46.50308 | 169.48669 | JGD, KJF & WRR | 4N_29 | WR730 | No | - | - | - |
| *Monoclea forsteri Hook.* | New Zealand | -43.71667 | 169.25000 | JGD, KJF & WRR | 4N_71 | WR768 | No | - | - | - |
| *Monoclea forsteri Hook.* | New Zealand | -43.43922 | 169.96317 | JGD, KJF & WRR | 4N_74 | WR771 | Yes | WR771-A | epGT_38 | Claroideoglomeraceae |
| *Monoclea forsteri Hook.* | New Zealand | -45.83264 | 170.49983 | JGD, KJF & WRR | 4N_8 | WR709 | No | - | - | - |
| *Monoclea forsteri Hook.* | New Zealand | -43.47000 | 170.01667 | JGD, KJF & WRR | 4N_80 | WR777 | Yes | WR777-B, WR777-D | epGT_52, epGT_25 | Undescribed Archeaosporales A, Glomeraceae |
| *Monoclea forsteri Hook.* | New Zealand | -42.90500 | 170.81667 | JGD, KJF & WRR | 4N_83 | WR780 | Yes | WR780-D | epGT_38 | Claroideoglomeraceae |
| *Monoclea forsteri Hook.* | New Zealand | -42.33833 | 171.47167 | JGD, KJF & WRR | 4N_89 | WR786 | No | - | - | - |
| *Monoclea forsteri Hook.* | New Zealand | -39.40000 | 176.42500 | JGD, KJF & WRR | 4NN_49 | WR682 | No | - | - | - |
| *Monoclea gottschei Lindb.* | Brazil | -22.96946 | -43.22479 | SP | 2BR8 | WR1161 | No | - | - | - |
| *Neohodgsonia mirabilis (Perss.) Perss.* | New Zealand | -42.80000 | 171.56250 | JGD & SP | 2N_1 | 8762, 8763 | No | - | - | - |
| *Neohodgsonia mirabilis (Perss.) Perss.* | New Zealand | -42.37500 | 172.39583 | JGD, KJF & MIB | 3N_102 | WR198 | Yes | WR198-A | epGT_38 | Claroideoglomeraceae |
| *Neohodgsonia mirabilis (Perss.) Perss.* | New Zealand | -41.20000 | 172.80833 | JGD, KJF & MIB | 3N_42 | WR159 | No | - | - | - |
| *Neohodgsonia mirabilis (Perss.) Perss.* | New Zealand | -41.22500 | 172.76667 | JGD, KJF & MIB | 3N_49 | WR164 | Yes | WR164-A | epGT_41 | Diversisporaceae |
| *Neohodgsonia mirabilis (Perss.) Perss.* | New Zealand | -42.25556 | 173.83333 | JGD, KJF & MIB | 3N_5 | WR143 | Yes | WR143-B | epGT_41 | Diversisporaceae |
| *Neohodgsonia mirabilis (Perss.) Perss.* | New Zealand | -41.22500 | 172.70000 | JGD, KJF & MIB | 3N_55 | WR170 | No | - | - | - |
| *Neohodgsonia mirabilis (Perss.) Perss.* | New Zealand | -44.82417 | 168.10750 | JGD, KJF & WRR | 4N_52 | WR751 | Yes | WR751-C | epGT_40 | Claroideoglomeraceae |
| *Neohodgsonia mirabilis (Perss.) Perss.* | New Zealand | -42.57167 | 171.47000 | JGD, KJF & WRR | 4N_87 | WR784 | Yes | WR784-A | epGT_38 | Claroideoglomeraceae |
| *Noteroclada confluens Taylor ex Hook. f. & Wilson* | Falkland Islands | -51.61641 | -59.52163 | JGD | F11.B | 8538 | Yes | 8538.2-3 | epGT_04 | Glomeraceae |
| *Noteroclada confluens Taylor ex Hook. f. & Wilson* | Falkland Islands | -51.61401 | -59.52175 | JGD | F11.C | 8539 | No | - | - | - |
| *Noteroclada confluens Taylor ex Hook. f. & Wilson* | Falkland Islands | -51.69707 | -60.07882 | JGD | F15 | 8541 | Yes | 8541-3, 8541-4 | epGT_04, epGT_38 | Glomeraceae, Claroideoglomeraceae |
| *Noteroclada confluens Taylor ex Hook. f. & Wilson* | Falkland Islands | -51.89599 | -58.39153 | JGD | F48 | 8548 | Yes | 8548.2-1 | epGT_04 | Glomeraceae |
| *Oxymitra incrassata (Brot.) Sérgio & Sim-Sim* | Italy | 37.65773 | 14.89341 | JGD | sc18 | WR889 | No | - | - | - |
| *Pallavicinia xiphoides (Hook. f. & Taylor) Trevis.* | Falkland Islands | -51.69707 | -60.07882 | JGD | F13 | 8540 | Yes | 8540-2 | epGT_05 | Glomeraceae |
| *Pallavicinia xiphoides (Hook. f. & Taylor) Trevis.* | Falkland Islands | -51.40626 | -57.55999 | JGD | F31 | 8543 | Yes | 8543-1 | epGT_04 | Glomeraceae |
| *Pallavicinia xiphoides (Hook. f. & Taylor) Trevis.* | Falkland Islands | -51.74508 | -58.18785 | JGD | F39 | 8546 | Yes | 8546-4 | epGT_05 | Glomeraceae |
| *Pallavicinia xiphoides (Hook. f. & Taylor) Trevis.* | Falkland Islands | -51.78403 | -58.72350 | JGD | F57 | 8552 | Yes | 8552-1 | epGT_05 | Glomeraceae |
| *Pallavicinia xiphoides (Hook. f. & Taylor) Trevis.* | New Zealand | -41.22500 | 172.70000 | JGD, KJF & MIB | 3N_52 | WR167 | Yes | WR167-B | epGT_04 | Glomeraceae |
| *Pellia endiviifolia (Dicks.) Dumort.* | India | 31.10406 | 77.14475 | JGD & SP | IW42 | wr382 | Yes | WR382-C, WR382-D | epGT_57, epGT_57 | Archeosporaceae, Archeosporaceae |
| *Pellia endiviifolia (Dicks.) Dumort.* | Wales | 53.10877 | -4.03385 | JGD & SP | NW14 | WR613 | No | - | - | - |
| *Pellia epiphylla (L.) Corda* | England | 50.86947 | 0.62812 | JGD | Pe1 | WR241 | No | - | - | - |
| *Pellia epiphylla (L.) Corda* | England | 50.86704 | 0.63055 | JGD | Pe2 | WR242 | No | - | - | - |
| *Pellia epiphylla (L.) Corda* | England | 51.56896 | -0.16794 | JGD | Pe3 | WR247 | No | - | - | - |
| *Pellia epiphylla (L.) Corda* | England | 51.56895 | -0.16794 | JGD | Pe4 | WR248 | No | - | - | - |
| *Pellia epiphylla (L.) Corda* | France | 45.67485 | 6.88097 | JGD | 12H4 | WR1120 | No | - | - | - |
| *Pellia epiphylla (L.) Corda* | Iceland | 63.61594 | -19.99230 | SP | IC003 | WR955 | Yes | WR955-H | epGT_19 | Glomeraceae |
| *Pellia epiphylla (L.) Corda* | Ireland | 53.06575 | -6.45381 | JGD | 12H13 | WR1134 | No | - | - | - |
| *Pellia epiphylla (L.) Corda* | Ireland | 53.06360 | -6.45439 | JGD | 12H14 | WR1135 | No | - | - | - |
| *Pellia epiphylla (L.) Corda* | USA | 44.15334 | -71.68335 | JGD | NH129 | WR308 | No | - | - | - |
| *Pellia epiphylla (L.) Corda* | USA | 44.25927 | -71.25570 | JGD | NH31 | WR272 | Yes | WR272-A | epGT_13 | Glomeraceae |
| *Pellia epiphylla (L.) Corda* | USA | 44.26727 | -71.23222 | JGD | NH55 | WR284 | No | - | - | - |
| *Pellia epiphylla (L.) Corda* | USA | 44.25665 | -71.24552 | JGD | NH64 | WR286 | Yes | WR286-A | epGT_21 | Glomeraceae |
| *Pellia epiphylla (L.) Corda* | Wales | 53.10275 | -3.845714 | JGD & SP | NW5 | WR604 | Yes | WR604-D | epGT_46 | Acaulosporaceae |
| *Pellia neesiana (Gottsche) Limpr.* | England | 51.56726 | -0.17054 | JGD | Pn1 | WR249 | No | - | - | - |
| *Phyllothallia nivicola E.A. Hodgs* | New Zealand | -41.22500 | 172.70000 | JGD, KJF & MIB | 3N_53 | WR168 | No | - | - | - |
| *Phyllothallia nivicola E.A. Hodgs* | New Zealand | -41.21667 | 172.71667 | JGD, KJF & MIB | 3N_61A | WR173 | No | - | - | - |
| *Phyllothallia nivicola E.A. Hodgs* | New Zealand | -41.21667 | 172.71667 | JGD, KJF & MIB | 3N_61B | WR174 | No | - | - | - |
| *Plagiochasma eximium (Schiffner) Steph.* | Lesotho | -29.44240 | 27.70287 | JGD & SP | L25 | WR92, WR92.b | No | - | - | - |
| *Plagiochasma eximium (Schiffner) Steph.* | Lesotho | -29.48271 | 27.75326 | JGD & SP | L27 | WR94, WR94.b | No | - | - | - |
| *Plagiochasma eximium (Schiffner) Steph.* | Lesotho | -29.06914 | 28.39172 | JGD & SP | L41 | WR108, WR108.b | No | - | - | - |
| *Plagiochasma eximium (Schiffner) Steph.* | South Africa | -29.00419 | 29.42408 | JGD & SP | SA14 | WR42, WR42.b | No | - | - | - |
| *Plagiochasma eximium (Schiffner) Steph.* | South Africa | -29.00681 | 29.42283 | JGD & SP | SA9 | WR36, WR36.b | No | - | - | - |
| *Plagiochasma rupestre (J. R. Forst. & G. Forst.) Steph.* | Ascension Island | -7.94728 | -14.35070 | JGD & SP | AI16 | TS13 | Yes | TS13n.5 | epGT_07 | Glomeraceae |
| *Plagiochasma rupestre (J. R. Forst. & G. Forst.) Steph.* | Ascension Island | -7.95169 | -14.34757 | JGD & SP | AI18 | TS15 | Yes | TS15b.3 | epGT_31 | Glomeraceae |
| *Plagiochasma rupestre (J. R. Forst. & G. Forst.) Steph.* | Ascension Island | -7.95117 | -14.34665 | JGD & SP | AI188 | TS51 | Yes | TS51.2 | epGT_30 | Glomeraceae |
| *Plagiochasma rupestre (J. R. Forst. & G. Forst.) Steph.* | Ascension Island | -7.95051 | -14.33554 | JGD & SP | AI27 | TS24 | Yes | TS24.4 | epGT_31 | Glomeraceae |
| *Plagiochasma rupestre (J. R. Forst. & G. Forst.) Steph.* | Ascension Island | -7.94919 | -14.34427 | JGD & SP | AI44 | TS37 | Yes | TS37n.1, TS37n.4 | epGT_07, epGT_14 | Glomeraceae, Glomeraceae |
| *Plagiochasma rupestre (J. R. Forst. & G. Forst.) Steph.* | Ascension Island | -7.95884 | -14.34445 | JGD & SP | AI95 | TS40 | Yes | TS40b.1 | epGT_31 | Glomeraceae |
| *Plagiochasma rupestre (J. R. Forst. & G. Forst.) Steph.* | Ascension Island | -7.95047 | -14.34989 | JGD | F62 | 8557 | Yes | 8557-2, 8557-4 | epGT_31, epGT_07 | Glomeraceae, Glomeraceae |
| *Plagiochasma rupestre (J. R. Forst. & G. Forst.) Steph.* | India | 31.21769 | 77.43119 | JGD & SP | IW89 | wr435 | No | - | - | - |
| *Plagiochasma rupestre (J. R. Forst. & G. Forst.) Steph.* | Italy | 40.70813 | 14.84466 | JGD | Pl | 8619 | No | - | - | - |
| *Plagiochasma rupestre (J. R. Forst. & G. Forst.) Steph.* | Italy | 37.85135 | 15.28997 | JGD | sc11 | WR885 | No | - | - | - |
| *Plagiochasma rupestre (J. R. Forst. & G. Forst.) Steph.* | Italy | 37.07391 | 15.28536 | JGD | sc4 | WR881 | Yes | WR881-C | epGT_37 | Claroideoglomeraceae |
| *Plagiochasma rupestre (J. R. Forst. & G. Forst.) Steph.* | Italy | 37.85375 | 15.28836 | JGD | sc7 | WR884 | No | - | - | - |
| *Plagiochasma rupestre (J. R. Forst. & G. Forst.) Steph.* | Lesotho | -29.14869 | 27.68169 | JGD & SP | L22 | WR89 | No | - | - | - |
| *Plagiochasma rupestre (J. R. Forst. & G. Forst.) Steph.* | Lesotho | -29.48332 | 27.74900 | JGD & SP | L26 | WR93, WR93.b | No | - | - | - |
| *Plagiochasma rupestre (J. R. Forst. & G. Forst.) Steph.* | Lesotho | -29.34737 | 27.84863 | JGD & SP | L29 | WR96, WR96.b | No | - | - | - |
| *Plagiochasma rupestre (J. R. Forst. & G. Forst.) Steph.* | Lesotho | -29.42350 | 27.91636 | JGD & SP | L45 | WR111, WR111.b | No | - | - | - |
| *Plagiochasma rupestre (J. R. Forst. & G. Forst.) Steph.* | New Zealand | -36.98663 | 174.47456 | JGD & SP | 2NN_1 | 8815 | No | - | - | - |
| *Plagiochasma rupestre (J. R. Forst. & G. Forst.) Steph.* | New Zealand | -42.65000 | 173.32917 | JGD, KJF & MIB | 3N_1 | WR141, WR141.b | No | - | - | - |
| *Plagiochasma rupestre (J. R. Forst. & G. Forst.) Steph.* | South Africa | -29.00331 | 29.42424 | JGD & SP | SA17 | WR45, WR45.b | No | - | - | - |
| *Plagiochasma rupestre (J. R. Forst. & G. Forst.) Steph.* | South Africa | -29.00531 | 29.42344 | JGD & SP | SA18 | WR46 | Yes | WR46-A, WR46-B | epGT_39, epGT_58 | Claroideoglomeraceae, Archeosporaceae |
| *Plagiochasma rupestre (J. R. Forst. & G. Forst.) Steph.* | South Africa | -28.51347 | 28.61889 | JGD & SP | SA32 | WR60 | Yes | WR60-C | epGT_52 | Undescribed Archeaosporales A |
| *Plagiochasma sp.* | China | 25.392005 | 110.136049 | SP | CH5 | WR14, WR14.b | Yes | WR14.B-A | Singleton | Glomeraceae |
| *Plagiochasma sp.* | India | 26.86794 | 88.28483 | JGD & SP | IE2 | wr482 | No | - | - | - |
| *Plagiochasma sp.* | India | 27.04003 | 88.21850 | JGD & SP | IE34 | wr509 | No | - | - | - |
| *Plagiochasma sp.* | India | 27.06839 | 88.38844 | JGD & SP | IE59 | wr531 | No | - | - | - |
| *Plagiochasma sp.* | India | 27.03861 | 88.26361 | JGD & SP | IE78 | wr544 | Yes | WR544-C | epGT_34 | Glomeraceae |
| *Plagiochasma sp.* | India | 31.09967 | 77.18286 | JGD & SP | IW11 | wr356 | No | - | - | - |
| *Plagiochasma sp.* | India | 25.78563 | 84.73277 | JGD & SP | IW112 | wr458 | Yes | WR458-D | epGT_27 | Glomeraceae |
| *Plagiochasma sp.* | India | 31.09619 | 77.17853 | JGD & SP | IW115 | wr461 | No | - | - | - |
| *Plagiochasma sp.* | India | 31.11275 | 77.15022 | JGD & SP | IW126 | wr472 | Yes | WR472-C | epGT_11 | Glomeraceae |
| *Plagiochasma sp.* | India | 31.09756 | 77.18058 | JGD & SP | IW21 | wr364 | Yes | WR364-A, WR364-D | epGT_55, epGT_55 | Undescribed Archeaosporales B, Undescribed Archeaosporales B |
| *Plagiochasma sp.* | India | 31.09756 | 77.18058 | JGD & SP | IW23 | wr365 | No | - | - | - |
| *Plagiochasma sp.* | India | 31.09756 | 77.18058 | JGD & SP | IW30 | wr371 | No | - | - | - |
| *Plagiochasma sp.* | India | 31.09756 | 77.18058 | JGD & SP | IW33 | wr373 | No | - | - | - |
| *Plagiochasma sp.* | India | 31.09756 | 77.18058 | JGD & SP | IW34 | wr374 | Yes | WR374-A | epGT_42 | Diversisporaceae |
| *Plagiochasma sp.* | India | 31.09967 | 77.18286 | JGD & SP | IW5 | wr351 | No | - | - | - |
| *Plagiochasma sp.* | India | 31.10778 | 77.25389 | JGD & SP | IW58 | wr398 | Yes | WR398-F | epGT_42 | Diversisporaceae |
| *Plagiochasma sp.* | India | 31.09931 | 77.26333 | JGD & SP | IW63 | wr406 | No | - | - | - |
| *Plagiochasma sp.* | India | 31.09931 | 77.26333 | JGD & SP | IW64 | wr407 | No | - | - | - |
| *Plagiochasma sp.* | India | 31.10778 | 77.25389 | JGD & SP | IW67 | wr411 | Yes | WR411-D | epGT_36 | Claroideoglomeraceae |
| *Plagiochasma sp.* | India | 31.11847 | 77.14308 | JGD & SP | IW71 | wr415 | No | - | - | - |
| *Plagiochasma sp.* | India | 31.09667 | 77.27481 | JGD & SP | IW83 | wr429 | No | - | - | - |
| *Plagiochasma sp.* | India | 31.24347 | 77.50050 | JGD & SP | IW95 | wr440 | Yes | WR440-A, WR440-B | epGT_36, epGT_36 | Claroideoglomeraceae, Claroideoglomeraceae |
| *Podomitrium phyllanthus (Hook.) Mitt.* | New Zealand | -43.98333 | 168.60833 | JGD, KJF & MIB | 3N_135 | WR219 | Yes | WR219-A | epGT_04 | Glomeraceae |
| *Podomitrium phyllanthus (Hook.) Mitt.* | New Zealand | -43.45556 | 170.45556 | JGD, KJF & MIB | 3N_84 | WR186 | No | - | - | - |
| *Preissia quadrata (Scop.) Nees/Marchantia quadrata (Scop.)* | England | 54.22991 | -2.20303 | JGD | T7 | 8607 | Yes | 8607-1 | Singleton | Glomeraceae |
| *Preissia quadrata (Scop.) Nees/Marchantia quadrata (Scop.)* | Iceland | 63.61594 | -19.99230 | SP | IC006 | WR1062 | Yes | WR1062-A, WR1062-B | epGT_10, epGT_39 | Glomeraceae, Claroideoglomeraceae |
| *Preissia quadrata (Scop.) Nees/Marchantia quadrata (Scop.)* | Iceland | 63.94058 | -21.98383 | SP | IC127 | WR1069 | Yes | WR1069-G | epGT_42 | Diversisporaceae |
| *Preissia quadrata (Scop.) Nees/Marchantia quadrata (Scop.)* | Iceland | 63.94058 | -21.98383 | SP | IC146 | WR1075 | Yes | WR1075-A | epGT_06 | Glomeraceae |
| *Preissia quadrata (Scop.) Nees/Marchantia quadrata (Scop.)* | Iceland | 63.61594 | -19.99230 | SP | IC147 | WR1076 | Yes | WR1076-C, WR1076-D | epGT_06, epGT_49 | Glomeraceae, Acaulosporaceae |
| *Preissia quadrata (Scop.) Nees/Marchantia quadrata (Scop.)* | Ireland | 53.06284 | -6.45480 | JGD | 12H11 | WR1132 | Yes | WR1132-B | epGT_06 | Glomeraceae |
| *Preissia quadrata (Scop.) Nees/Marchantia quadrata (Scop.)* | Italy | 45.25797 | 6.88108 | JGD | 14pq | WR940 | Yes | WR940-D | epGT_31 | Glomeraceae |
| *Preissia quadrata (Scop.) Nees/Marchantia quadrata (Scop.)* | Italy | 46.25101 | 8.03816 | JGD | IS15 | WR633 | Yes | WR633-D | epGT_31 | Glomeraceae |
| *Preissia quadrata (Scop.) Nees/Marchantia quadrata (Scop.)* | Wales | 53.10877 | -4.03385 | JGD & SP | NW6 | WR605 | Yes | WR605-A, WR605-E | epGT_11, Singleton | Glomeraceae, Glomeraceae |
| *Reboulia hemisphaerica (L.) Raddi* | England | 51.15873 | -0.68907 | JGD | T2 | 8602 | No | - | - | - |
| *Reboulia hemisphaerica (L.) Raddi* | England | 54.24382 | -2.18939 | JGD | T5 | 8605 | No | - | - | - |
| *Reboulia hemisphaerica (L.) Raddi* | England | 54.15583 | -2.24897 | JGD | T9 | 8609 | No | - | - | - |
| *Reboulia hemisphaerica (L.) Raddi* | France | 45.67485 | 6.88097 | JGD | 12H2 | WR1118 | No | - | - | - |
| *Reboulia hemisphaerica (L.) Raddi* | Italy | 40.70765 | 14.84338 | JGD | R1 | 8621 | No | - | - | - |
| *Reboulia hemisphaerica (L.) Raddi* | Italy | 37.65773 | 14.89341 | JGD | sc17 | WR888 | No | - | - | - |
| *Reboulia hemisphaerica (L.) Raddi* | Italy | 37.85899 | 15.27638 | JGD | sc2 | WR880 | No | - | - | - |
| *Reboulia hemisphaerica (L.) Raddi* | Italy | 37.07391 | 15.28536 | JGD | sc6 | WR883 | Yes | Low quality sequence but confirmed to be AMF | - | - |
| *Reboulia hemisphaerica (L.) Raddi* | New Zealand | -41.20556 | 172.82500 | JGD, KJF & MIB | 3N_43 | WR160 | Yes | WR160-A, WR160-C | epGT_26, epGT_11 | Glomeraceae, Glomeraceae |
| *Sauteria alpina (Nees & Bisch.) Nees* | India | 31.24347 | 77.50050 | JGD & SP | IW98 | wr446 | No | - | - | - |
| *Sauteria alpina (Nees & Bisch.) Nees* | Switzerland | 46.48255 | 8.38711 | JGD | IS9 | WR627 | No | - | - | - |
| *Sewardiella tuberifera Kashyap* | India | 31.09803 | 77.18667 | JGD & SP | IW39 | wr379 | Yes | WR379-D | epGT_42 | Diversisporaceae |
| *Sewardiella tuberifera Kashyap* | India | 31.10508 | 77.14392 | JGD & SP | IW49 | wr387 | Yes | WR387-A, WR387-D | epGT_08, Singleton | Glomeraceae, Archaeosporales |
| *Sewardiella tuberifera Kashyap* | India | 31.10778 | 77.25389 | JGD & SP | IW68 | wr412 | No | - | - | - |
| *Symphyogyna brasiliensis Nees* | Ascension Island | -7.95117 | -14.34665 | JGD & SP | AI184 | TS47 | Yes | TS47.1 | epGT_41 | Diversisporaceae |
| *Symphyogyna brasiliensis Nees* | Ascension Island | -7.94916 | -14.33666 | JGD & SP | AI24 | TS21 | Yes | TS21.2, TS21.4 | epGT_23, Singleton | Glomeraceae, Glomeraceae |
| *Symphyogyna brasiliensis Nees* | Ascension Island | -7.95051 | -14.33554 | JGD & SP | AI31 | TS27 | Yes | TS27.4 | epGT_23 | Glomeraceae |
| *Symphyogyna brasiliensis Nees* | Ascension Island | -7.95424 | -14.33612 | JGD & SP | AI32 | TS28 | No | - | - | - |
| *Symphyogyna brasiliensis Nees* | Ascension Island | -7.95183 | -14.35385 | JGD & SP | AI46 | TS38 | Yes | TS38.3 | epGT_23 | Glomeraceae |
| *Symphyogyna brasiliensis Nees* | Ascension Island | -7.95286 | -14.34847 | JGD & SP | AI7 | TS6 | Yes | TS6.1 | Singleton | Diversisporales |
| *Symphyogyna brasiliensis Nees* | Ascension Island | -7.95047 | -14.34989 | JGD | F63 | 8558 | No | - | - | - |
| *Symphyogyna brasiliensis Nees* | Brazil | -22.97328 | -43.25040 | SP | 2BR5 | WR1159 | Yes | WR1159-A | epGT_21 | Glomeraceae |
| *Symphyogyna brasiliensis Nees* | Colombia | 7.69533 | -74.02985 | JGD | Co9 | WR1143 | Yes | WR1143-D | epGT_30 | Glomeraceae |
| *Symphyogyna brongniartii Mont.* | Brazil | -22.95886 | -43.27752 | SP | 2BR2 | WR1157 | No | - | - | - |
| *Symphyogyna brongniartii Mont.* | Colombia | 7.68090 | -74.01162 | JGD | Co22 | WR1151 | Yes | WR1151-D | epGT_48 | Acaulosporaceae |
| *Symphyogyna hochstetteri Nees & Mont.* | Falkland Islands | -51.78403 | -58.72350 | JGD | F55 | 8551 | Yes | 8551-3 | epGT_05 | Glomeraceae |
| *Symphyogyna hochstetteri Nees & Mont.* | New Zealand | -42.37500 | 172.39583 | JGD, KJF & MIB | 3N_103 | WR199 | Yes | WR199-A | epGT_38 | Claroideoglomeraceae |
| *Symphyogyna hymenophyllum (Hook.) Mont. & Nees* | New Zealand | -43.44583 | 169.97500 | JGD, KJF & MIB | 3N_111 | WR205 | No | - | - | - |
| *Symphyogyna hymenophyllum (Hook.) Mont. & Nees* | New Zealand | -44.06250 | 169.35833 | JGD, KJF & MIB | 3N_121 | WR209 | Yes | WR209-D | epGT_45 | Acaulosporaceae |
| *Symphyogyna hymenophyllum (Hook.) Mont. & Nees* | New Zealand | -43.98333 | 168.60000 | JGD, KJF & MIB | 3N_131 | WR215 | Yes | WR215-A, WR215-D | epGT_25, Singleton | Glomeraceae, Archaeosporales |
| *Symphyogyna hymenophyllum (Hook.) Mont. & Nees* | New Zealand | -41.87500 | 171.49167 | JGD, KJF & MIB | 3N_65 | WR178 | No | - | - | - |
| *Symphyogyna hymenophyllum (Hook.) Mont. & Nees* | New Zealand | -42.25556 | 173.83333 | JGD, KJF & MIB | 3N_9 | WR146 | Yes | WR146-A, WR146-B | epGT_41, epGT_56 | Diversisporaceae, Undescribed Archeaosporales B |
| *Symphyogyna hymenophyllum (Hook.) Mont. & Nees* | New Zealand | -39.15500 | 175.76667 | JGD, KJF & WRR | 4NN_20 | WR660 | Yes | WR660-A | epGT_11 | Glomeraceae |
| *Targionia hypophylla L.* | Colombia | 7.68090 | -74.01162 | JGD | Co22a | WR1152 | No | - | - | - |
| *Targionia hypophylla L.* | India | 26.86794 | 88.28483 | JGD & SP | IE1 | wr481 | Yes | WR481-B, WR481-D | epGT_01, epGT_01 | Glomeraceae, Glomeraceae |
| *Targionia hypophylla L.* | India | 27.04003 | 88.21850 | JGD & SP | IE36 | wr511 | No | - | - | - |
| *Targionia hypophylla L.* | India | 27.03861 | 88.26361 | JGD & SP | IE76 | wr542 | No | - | - | - |
| *Targionia hypophylla L.* | India | 25.78379 | 84.72812 | JGD & SP | IW114a | wr460 | No | - | - | - |
| *Targionia hypophylla L.* | India | 31.09878 | 77.18156 | JGD & SP | IW20 | wr443 | No | - | - | - |
| *Targionia hypophylla L.* | India | 31.09756 | 77.18058 | JGD & SP | IW29 | wr370 | No | - | - | - |
| *Targionia hypophylla L.* | India | 31.09967 | 77.18286 | JGD & SP | IW3 | wr349 | No | - | - | - |
| *Targionia hypophylla L.* | India | 31.10508 | 77.14392 | JGD & SP | IW51a | wr390 | No | - | - | - |
| *Targionia hypophylla L.* | Italy | 45.77060 | 8.43172 | JGD | 14Th1 | WR937 | No | - | - | - |
| *Targionia hypophylla L.* | Italy | 45.77060 | 8.43172 | JGD | 14Th2 | WR938 | No | - | - | - |
| *Targionia hypophylla L.* | Italy | 45.79685 | 8.40987 | JGD | 14Th3 | WR939 | No | - | - | - |
| *Targionia hypophylla L.* | New Zealand | -43.60556 | 172.65000 | JGD, KJF & MIB | 3N_161 | WR235 | No | - | - | - |
| *Targionia hypophylla L.* | New Zealand | -43.60556 | 172.65000 | JGD, KJF & MIB | 3N_162 | WR236 | No | - | - | - |
| *Targionia hypophylla L.* | USA | 35.99714 | -121.47842 | JGD | CA11 | 8632 | No | - | - | - |
| *Targionia hypophylla L.* | USA | 35.99889 | -121.46814 | JGD | CA15 | 8636 | No | - | - | - |
| *Targionia hypophylla L.* | USA | 36.53714 | -118.78233 | JGD | CA18 | 8639 | No | - | - | - |
| *Targionia hypophylla L.* | Colombia | 7.67813 | -74.02133 | JGD | Co11 | WR1144 | No | - | - | - |
| *Targionia hypophylla L.* | Lesotho | -29.34986 | 27.85103 | JGD & SP | L32 | WR99 | No | - | - | - |
| *Targionia hypophylla L.* | Lesotho | -29.06914 | 28.39172 | JGD & SP | L40 | WR107 | No | - | - | - |
| *Targionia hypophylla L.* | Lesotho | -29.42350 | 27.91636 | JGD & SP | L46 | WR112, WR112.b | No | - | - | - |
| *Targionia hypophylla L.* | Lesotho | -28.76403 | 28.64683 | JGD & SP | L5 | WR70 | Yes | WR70-B | epGT_26 | Glomeraceae |
| *Targionia hypophylla L.* | South Africa | -29.00531 | 29.42344 | JGD & SP | SA16 | WR44, WR44.b | Yes | WR44.B-A | epGT_55 | Undescribed Archeaosporales B |
| *Targionia hypophylla L.* | South Africa | -29.27613 | 29.51911 | JGD & SP | SA27 | WR55, WR55.b | Yes | WR55.B-D | epGT_30 | Glomeraceae |
| *Targionia hypophylla L.* | South Africa | -29.00681 | 29.42283 | JGD & SP | SA8 | WR35 | No | - | - | - |
| *Targionia hypophylla L.* | Wales | 52.22258 | -3.08003 | JGD | 12H10a | WR1129 | No | - | - | - |
| *Targionia hypophylla L.* | Wales | 52.22312 | -3.07970 | JGD | 12H10b | WR1130 | No | - | - | - |
| *Targionia hypophylla L.* | Wales | 52.22298 | -3.07842 | JGD | 12H10c | WR1131 | No | - | - | - |
| *Targionia hypophylla L.* | Wales | 52.85592 | -3.37886 | JGD | 12H9a | WR1127 | No | - | - | - |
| *Targionia hypophylla L.* | Wales | 52.85506 | -3.37842 | JGD | 12H9b | WR1128 | No | - | - | - |
| *Targionia hypophylla L.* | Italy | 37.29200 | 13.58153 | JGD | sc23 | WR891 | No | - | - | - |
| *Targionia hypophylla L.* | Italy | 37.29200 | 13.58153 | JGD | sc24 | WR892 | Yes | WR892-B, WR892-D | epGT_54, epGT_37 | Undescribed Archeaosporales B, Claroideoglomeraceae |
| *Targionia hypophylla L.* | Italy | 37.29200 | 13.58153 | JGD | sc28 | WR895 | Yes | WR895-B, WR895-D | epGT_54, epGT_56 | Undescribed Archeaosporales B, Undescribed Archeaosporales B |
| *Targionia hypophylla L.* | Italy | 37.28875 | 13.60044 | JGD | sc33 | WR876 | Yes | WR876-D | Singleton | Claroideoglomeraceae |
| *Treubia lacunosa (Colenso) Prosk.* | New Zealand | -42.27500 | 172.08333 | JGD & SP | 2N_113 | 8806, 8807 | No | - | - | - |
| *Treubia lacunosa (Colenso) Prosk.* | New Zealand | -42.27500 | 172.08333 | JGD & SP | 2N_117 | 8811, 8812, 8812.b | No | - | - | - |
| *Treubia lacunosa (Colenso) Prosk.* | New Zealand | -42.89583 | 171.12500 | JGD & SP | 2N_37 | 8777 | No | - | - | - |
| *Treubia lacunosa (Colenso) Prosk.* | New Zealand | -42.37500 | 172.39583 | JGD, KJF & MIB | 3N_100 | WR196 | No | - | - | - |
| *Treubia lacunosa (Colenso) Prosk.* | New Zealand | -43.44583 | 169.97500 | JGD, KJF & MIB | 3N_110 | WR204 | No | - | - | - |
| *Treubia lacunosa (Colenso) Prosk.* | New Zealand | -44.06250 | 169.35833 | JGD, KJF & MIB | 3N_119 | WR207 | No | - | - | - |
| *Treubia lacunosa (Colenso) Prosk.* | New Zealand | -44.08889 | 169.35000 | JGD, KJF & MIB | 3N_125 | WR212 | No | - | - | - |
| *Treubia lacunosa (Colenso) Prosk.* | New Zealand | -43.90833 | 171.25833 | JGD, KJF & MIB | 3N_141 | WR221 | No | - | - | - |
| *Treubia lacunosa (Colenso) Prosk.* | New Zealand | -41.20000 | 172.80833 | JGD, KJF & MIB | 3N_25 | WR156 | No | - | - | - |
| *Treubia lacunosa (Colenso) Prosk.* | New Zealand | -41.20833 | 172.83889 | JGD, KJF & MIB | 3N_45 | WR162 | No | - | - | - |
| *Treubia lacunosa (Colenso) Prosk.* | New Zealand | -42.95000 | 170.71667 | JGD, KJF & MIB | 3N_72 | WR181 | No | - | - | - |
| *Treubia lacunosa (Colenso) Prosk.* | New Zealand | -42.37500 | 172.39583 | JGD, KJF & MIB | 3N_96 | WR193 | No | - | - | - |
| *Treubia lacunosa (Colenso) Prosk.* | New Zealand | -41.18667 | 172.15750 | JGD, KJF & WRR | 4N_107-1 | WR803 | No | - | - | - |
| *Treubia lacunosa (Colenso) Prosk.* | New Zealand | -41.18667 | 172.15750 | JGD, KJF & WRR | 4N_107-2 | WR804 | No | - | - | - |
| *Treubia lacunosa (Colenso) Prosk.* | New Zealand | -41.13833 | 172.18333 | JGD, KJF & WRR | 4N_113 | WR809 | No | - | - | - |
| *Treubia lacunosa (Colenso) Prosk.* | New Zealand | -41.00000 | 172.16667 | JGD, KJF & WRR | 4N_119 | WR814 | No | - | - | - |
| *Treubia lacunosa (Colenso) Prosk.* | New Zealand | -41.42417 | 172.10000 | JGD, KJF & WRR | 4N_138 | WR833 | No | - | - | - |
| *Treubia lacunosa (Colenso) Prosk.* | New Zealand | -46.58333 | 169.35000 | JGD, KJF & WRR | 4N_15 | WR716 | No | - | - | - |
| *Treubia lacunosa (Colenso) Prosk.* | New Zealand | -42.80000 | 171.55750 | JGD, KJF & WRR | 4N_150 | WR843 | No | - | - | - |
| *Treubia lacunosa (Colenso) Prosk.* | New Zealand | -46.58333 | 169.35000 | JGD, KJF & WRR | 4N_17 | WR718 | No | - | - | - |
| *Treubia lacunosa (Colenso) Prosk.* | New Zealand | -46.57995 | 169.43821 | JGD, KJF & WRR | 4N_22 | WR723 | No | - | - | - |
| *Treubia lacunosa (Colenso) Prosk.* | New Zealand | -46.50308 | 169.48669 | JGD, KJF & WRR | 4N_24 | WR725 | No | - | - | - |
| *Treubia lacunosa (Colenso) Prosk.* | New Zealand | -46.50308 | 169.48669 | JGD, KJF & WRR | 4N_28 | WR729 | No | - | - | - |
| *Treubia lacunosa (Colenso) Prosk.* | New Zealand | -46.52000 | 169.55500 | JGD, KJF & WRR | 4N_29a | WR731 | No | - | - | - |
| *Treubia lacunosa (Colenso) Prosk.* | New Zealand | -46.26667 | 167.90500 | JGD, KJF & WRR | 4N_33 | WR735 | No | - | - | - |
| *Treubia lacunosa (Colenso) Prosk.* | New Zealand | -46.26667 | 167.90500 | JGD, KJF & WRR | 4N_36 | WR738 | No | - | - | - |
| *Treubia lacunosa (Colenso) Prosk.* | New Zealand | -44.67167 | 167.92417 | JGD, KJF & WRR | 4N_57 | WR756 | No | - | - | - |
| *Treubia lacunosa (Colenso) Prosk.* | New Zealand | -44.15750 | 169.26667 | JGD, KJF & WRR | 4N_64 | WR762 | No | - | - | - |
| *Treubia lacunosa (Colenso) Prosk.* | New Zealand | -44.10750 | 169.35000 | JGD, KJF & WRR | 4N_67 | WR765 | No | - | - | - |
| *Treubia lacunosa (Colenso) Prosk.* | New Zealand | -43.43922 | 169.96317 | JGD, KJF & WRR | 4N_72 | WR769 | No | - | - | - |
| *Treubia lacunosa (Colenso) Prosk.* | New Zealand | -43.43922 | 169.96317 | JGD, KJF & WRR | 4N_73 | WR770 | No | - | - | - |
| *Treubia lacunosa (Colenso) Prosk.* | New Zealand | -42.90500 | 170.81667 | JGD, KJF & WRR | 4N_84 | WR781 | No | - | - | - |
| *Treubia lacunosa (Colenso) Prosk.* | New Zealand | -42.57167 | 171.47000 | JGD, KJF & WRR | 4N_85 | WR782 | No | - | - | - |
| *Treubia lacunosa (Colenso) Prosk.* | New Zealand | -42.33833 | 171.47167 | JGD, KJF & WRR | 4N_88 | WR785 | No | - | - | - |
| *Treubia lacunosa (Colenso) Prosk.* | New Zealand | -42.15000 | 171.77167 | JGD, KJF & WRR | 4N_94 | WR791 | No | - | - | - |
| *Treubia lacunosa (Colenso) Prosk.* | New Zealand | -39.15500 | 175.76667 | JGD, KJF & WRR | 4NN_21 | WR661 | No | - | - | - |
| *Treubia lacunosa (Colenso) Prosk.* | New Zealand | -39.26667 | 175.38667 | JGD, KJF & WRR | 4NN_27 | WR667 | No | - | - | - |
| *Treubia lacunosa (Colenso) Prosk.* | New Zealand | -39.32917 | 175.49583 | JGD, KJF & WRR | 4NN_36 | WR672 | No | - | - | - |
| *Treubia lacunosa (Colenso) Prosk.* | New Zealand | -38.36667 | 176.61667 | JGD, KJF & WRR | 4NN_4 | WR646 | No | - | - | - |
| *Treubia lacunosa (Colenso) Prosk.* | New Zealand | -39.40000 | 176.42500 | JGD, KJF & WRR | 4NN_48 | WR681 | No | - | - | - |
| *Treubia lacunosa (Colenso) Prosk.* | New Zealand | -39.38333 | 176.43889 | JGD, KJF & WRR | 4NN_52 | WR684 | No | - | - | - |
| *Treubia lacunosa (Colenso) Prosk.* | New Zealand | -38.78333 | 176.23333 | JGD, KJF & WRR | 4NN_64 | WR696 | No | - | - | - |
| *Treubia lacunosa (Colenso) Prosk.* | New Zealand | -38.53833 | 176.71667 | JGD, KJF & WRR | 4NN_7 | WR649 | No | - | - | - |
| *Treubia lacunosa (Colenso) Prosk.* | New Zealand | -46.32417 | 167.82417 | JGD, KJF & WRR | 4N_38 | WR740 | No | - | - | - |
| *Treubia lacunosa (Colenso) Prosk.* | New Zealand | -46.32417 | 167.82417 | JGD, KJF & WRR | 4N_39 | WR741 | No | - | - | - |
| *Treubia pygmaea R.M. Schust.* | New Zealand | -42.29105 | 172.06208 | JGD & SP | 2N_121 | 8813, 8813.b, 8814, 8814.b | No | - | - | - |
| *Treubia pygmaea R.M. Schust.* | New Zealand | -43.44583 | 169.97500 | JGD, KJF & MIB | 3N_108 | WR202 | No | - | - | - |
| *Treubia pygmaea R.M. Schust.* | New Zealand | -42.32000 | 172.11667 | JGD, KJF & WRR | 4N_102 | WR800 | No | - | - | - |
| *Treubia pygmaea R.M. Schust.* | New Zealand | -42.92167 | 171.55750 | JGD, KJF & WRR | 4N_152 | WR845 | No | - | - | - |
| *Treubia pygmaea R.M. Schust.* | New Zealand | -42.92417 | 171.55750 | JGD, KJF & WRR | 4N_153 | WR846 | No | - | - | - |
| *Treubia pygmaea R.M. Schust.* | New Zealand | -44.75750 | 167.95750 | JGD, KJF & WRR | 4N_53 | WR752 | No | - | - | - |
| *Treubia pygmaea R.M. Schust.* | New Zealand | -44.70333 | 167.95750 | JGD, KJF & WRR | 4N_62 | WR760 | No | - | - | - |
| *Treubia pygmaea R.M. Schust.* | New Zealand | -39.32917 | 175.49583 | JGD, KJF & WRR | 4NN_38 | WR673 | No | - | - | - |
| *Treubia pygmaea R.M. Schust.* | New Zealand | -39.32917 | 175.49583 | JGD, KJF & WRR | 4NN_39 | WR674 | No | - | - | - |
| *Treubia pygmaea R.M. Schust.* | New Zealand | -39.36250 | 175.47917 | JGD, KJF & WRR | 4NN_40 | WR675 | No | - | - | - |
| *Treubia pygmaea R.M. Schust.* | New Zealand | -39.36250 | 175.47917 | JGD, KJF & WRR | 4NN_41 | WR676 | No | - | - | - |

**Table S2. Summary of liverwort genera analysed and their colonising Glomeromycotina.** Some plant samples could only be identified to the genus level, as such an asterisk indicates this is the minimum number of different species analysed for the genus. In some cases Glomeromycotina order is given instead of family as the sequence could only be confidently classified to order. Country initials are as follows: Ascension Island (AI), Australia (AU), Brazil (BR), Chile (CL), China (CH), Colombia (CO), England (EN), Falkland Islands (FI), France (FR), Germany (GE), Iceland (IC), India (IN), Indonesia (ID), Ireland (IR), Italy (IT), Lesotho (LE), Malaysia (MA), New Zealand (NZ), South Africa (SA), Spain (SP), Switzerland (SW), USA (US), Vietnam (VI), Wales (WA).

| **Order** | **Family** | **Genus** | **Number of samples** | **Number of species** | **Collection location** | **AMF detected** |
| --- | --- | --- | --- | --- | --- | --- |
| **Haplomitriopsida** | |  |  |  |  |  |
| Treubiales | Treubiaceae | *Treubia* | 56 | 2 | NZ | - |
| Calobryales | Haplomitriaceae | *Haplomitrium* | 16 | 5 | CH, IN, NZ, US, WA | - |
|  |  |  |  |  |  |  |
| **Marchantiopsida** | |  |  |  |  |  |
| Neohodgsoniales | Neohodgsoniaceae | *Neohodgsonia* | 8 | 1 | NZ | Claroideoglomeraceae, Diversisporaceae, Archaeosporales |
| Lunulariales | Lunulariaceae | *Lunularia* | 36 | 1 | AU, BR, CO, EN, GE, IN, IT, NZ, SA, SP, US | Claroideoglomeraceae, Glomeraceae, Acaulosporaceae |
| Marchantiales | Marchantiaceae | *Marchantia* | 63 | 10* | AI, BR, CL, CO, FI, FR, IC, IN, IT, LE, MA, NZ, SA | Claroideoglomeraceae, Glomeraceae, Diversisporaceae, Archaeosporaceae, Undescribed Archaeosporales A |
|  |  | *Preissia* | 9 | 1 | EN, IC, IR, IT, WA | Claroideoglomeraceae, Glomeraceae, Acaulosporaceae, Diversisporaceae |
|  | Aytoniaceae | *Asterella* | 81 | 12* | AU, CO, IN, LE, NZ, SA, SW, US | Claroideoglomeraceae, Glomeraceae, Diversisporaceae, Archaeosporales |
|  |  | *Cryptomitrium* | 6 | 2 | IN, LE | - |
|  |  | *Mannia* | 5 | 1* | CH, IT, IN | Glomeraceae |
|  |  | *Plagiochasma* | 48 | 2* | AI, CH, IN, IT, LE, NZ, SA | Claroideoglomeraceae, Glomeraceae, Diversisporaceae, Archaeosporaceae, Undescribed Archaeosporales A & B |
|  |  | *Reboulia* | 9 | 1 | EN, FR, IT, NZ | Glomeraceae |
|  | Cleveaceae | *Athalamia* | 1 | 1 | IN | Diversisporaceae |
|  |  | *Clevea* | 2 | 1 | IN | Diversisporaceae, Diversisporales |
|  |  | *Sauteria* | 2 | 1 | IN, SW | - |
|  | Conocephalaceae | *Conocephalum* | 30 | 3 | CH, EN, IC, IN, IR, IT, LE, US, WA | Claroideoglomeraceae, Glomeraceae, Undescribed Archaeosporales A |
|  | Cyathodiaceae | *Cyathodium* | 7 | 3* | BR, CO, IN | - |
|  | Corsiniaceae | *Corsinia* | 4 | 1 | IT | - |
|  | Oxymitraceae | *Oxymitra* | 1 | 1 | IT | - |
|  | Targioniaceae | *Targionia* | 34 | 1 | CO, IN, IT, LE, NZ, SA, US, WA | Claroideoglomeraceae, Glomeraceae, Undescribed Archaeosporales B |
|  | Monocleaceae | *Monoclea* | 34 | 2 | BR, NZ | Claroideoglomeraceae, Glomeraceae, Diversisporales, Undescribed Archaeosporales A |
|  | Dumortieraceae | *Dumortiera* | 31 | 1 | BR, CH, CO, EN, IN, ID, SA, VI | Claroideoglomeraceae, Glomeraceae, Acaulosporaceae, Diversisporaceae, Undescribed Archaeosporales A, Paraglomeraceae |
|  |  |  |  |  |  |  |
| **Pelliidae** |  |  |  |  |  |  |
| Pelliales | Noterocladaceae | *Noteroclada* | 4 | 1 | FI | Claroideoglomeraceae, Glomeraceae |
|  | Pelliaceae | *Pellia* | 16 | 3 | EN, FR, IC, IN, IR, US, WA | Glomeraceae, Acaulosporaceae, Archaeosporaceae |
| Fossombroniales | Calyculariaceae | *Calycularia* | 10 | 1 | CH, IN | Glomeraceae, Acaulosporaceae, Undescribed Archaeosporales A |
|  |  | *Sewardiella* | 3 | 1 | IN | Glomeraceae, Diversisporaceae, Archeaosporales |
|  |  |  |  |  |  |  |
|  | Fossombroniaceae | *Allisonia* | 7 | 1 | NZ | Claroideoglomeraceae, Glomeraceae, Archaeosporales |
|  |  | *Fossombronia* | 116 | 15* | AI, AU, BR, CH, EN, IC, IN, IT, LE, NZ, SA, SP, US, WA | Claroideoglomeraceae, Glomeraceae, Acaulosporaceae, Diversisporaceae, Gigasporaceae, Diversisporales, Archaeosporaceae, Undescribed Archaeosporales A & B |
| Pallaviciniales | Phyllothalliaceae | *Phyllothallia* | 3 | 1 | NZ | - |
|  | Moerckiaceae | *Moerckia* | 1 | 1 | SW | Glomeraceae, Diversisporales |
|  | Hymenophytaceae | *Hymenophyton* | 4 | 1 | NZ | Acaulosporaceae |
|  | Pallaviciaceae | *Jensenia* | 1 | 1 | FI | Claroideoglomeraceae, Glomeraceae |
|  |  | *Pallavicinia* | 5 | 1 | FI, NZ | Glomeraceae |
|  |  | *Podomitrium* | 2 | 1 | NZ | Glomeraceae |
|  |  | *Symphyogyna* | 19 | 4 | AI, BR, CO, FI, NZ | Claroideoglomeraceae, Glomeraceae, Acaulosporaceae, Diversisporaceae, Diversisporales, Archaeosporales, Undescribed Archaeosporales B |

**Table S3. Glomeromycotina detection rate.** The numbers of samples colonised by Glomeromycotina. A total of 257 samples contained Glomeromycotina, of which 255 produced unambiguous sequences used in phylogenetic analysis. For some samples the Glomeromycotina could only be assigned to order and not family. The number in brackets gives the colonisation detection rate. The detection rates of orders/families are out of the Glomeromycotina samples and not all samples. The total percentages exceed 100% as 23 samples were colonised by more than one family.

|  | All samples | Haplomitriopsida | Marchantiopsida | Pelliidae |
| --- | --- | --- | --- | --- |
|  |  |  |  |  |
| Glomerales | 205 (80%) | - | 142 (88%) | 63 (67%) |
| Glomeraceae | 162 (63%) |  | 108 (67%) | 54 (57%) |
| Claroideoglomeraceae | 43 (17%) |  | 34 (21%) | 9 (10%) |
|  |  |  |  |  |
| Diversisporales | 36 (14%) | - | 16 (10%) | 20 (22%) |
| Acaulosporaceae | 12 (5%) |  | 3 (2%) | 9 (10%) |
| Diversisporaceae | 18 (7%) |  | 11 (7%) | 7 (8%) |
| Gigasporaceae | 1 (0.4%) |  | 0 | 1 (1%) |
|  |  |  |  |  |
| Archaeosporales | 37 (14%) | - | 17 (10%) | 20 (21%) |
| Archaeosporaceae | 9 (4%) |  | 3 (2%) | 6 (6%) |
| Undescribed Archaeosporales A | 14 (5%) |  | 8 (5%) | 6 (6%) |
| Undescribed Archaeosporales B | 9 (4%) |  | 4 (2%) | 5 (5%) |
|  |  |  |  |  |
| Paraglomerales | 1 (0.4%) | - | 1 (1%) | 0 |
| Paraglomeraceae | 1 (0.4%) |  | 1 (1%) |  |
|  |  |  |  |  |
| **Total** |  |  |  |  |
| Number of samples | 674 | 72 | 411 | 191 |
| Samples colonised by Glomeromycotina | 257 (38%) | 0 | 163 (40%) | 94 (49%) |

**Table S4. List of the virtual taxa found to colonise liverworts, hornworts, lycopods and ferns.**

| Virtual taxa ID | *Genus* | Family |
| --- | --- | --- |
| VTX00005 | *Archaeospora* | Archaeosporaceae |
| VTX00015 | *Acaulospora* | Acaulosporaceae |
| VTX00023 | *Acaulospora* | Acaulosporaceae |
| VTX00056 | *Claroideoglomus* | Claroideoglomeraceae |
| VTX00057 | *Claroideoglomus* | Claroideoglomeraceae |
| VTX00061 | *Diversispora* | Diversisporaceae |
| VTX00062 | *Diversispora* | Diversisporaceae |
| VTX00064 | *Septoglomus* | Glomeraceae |
| VTX00065 | *Glomus* | Glomeraceae |
| VTX00069 | *Glomus* | Glomeraceae |
| VTX00072 | *Glomus* | Glomeraceae |
| VTX00074 | *Glomus* | Glomeraceae |
| VTX00084 | *Glomus* | Glomeraceae |
| VTX00088 | *Glomus* | Glomeraceae |
| VTX00089 | *Glomus* | Glomeraceae |
| VTX00090 | *Glomus* | Glomeraceae |
| VTX00092 | *Glomus* | Glomeraceae |
| VTX00108 | *Glomus* | Glomeraceae |
| VTX00112 | *Glomus* | Glomeraceae |
| VTX00113 | *Glomus* | Glomeraceae |
| VTX00114 | *Glomus* | Glomeraceae |
| VTX00115 | *Glomus* | Glomeraceae |
| VTX00125 | *Glomus* | Glomeraceae |
| VTX00149 | *Glomus* | Glomeraceae |
| VTX00153 | *Glomus* | Glomeraceae |
| VTX00163 | *Glomus* | Glomeraceae |
| VTX00174 | *Glomus* | Glomeraceae |
| VTX00193 | *Entrophospora* | Acaulosporaceae |
| VTX00199 | *Glomus* | Glomeraceae |
| VTX00219 | *Glomus* | Glomeraceae |
| VTX00223 | *Glomus* | Glomeraceae |
| VTX00269 | *Glomus* | Glomeraceae |
| VTX00295 | *Glomus* | Glomeraceae |
| VTX00309 | *Glomus* | Glomeraceae |
| VTX00327 | *Glomus* | Glomeraceae |
| VTX00328 | *Acaulospora* | Acaulosporaceae |
| VTX00329 | *Glomus* | Glomeraceae |
| VTX00338 | *Archaeospora* | Archaeosporaceae |
| VTX00345 | *Glomus* | Glomeraceae |
| VTX00362 | *Glomus* | Glomeraceae |
| VTX00372 | *Glomus* | Glomeraceae |
| VTX00386 | *Glomus* | Glomeraceae |
| VTX00387 | *Glomus* | Glomeraceae |
| VTX00410 | *Glomus* | Glomeraceae |
| VTX00414 | *Glomus* | Glomeraceae |

**Table S5. Non-Glomeraceae detection rates in surveys of AMF in plants.** The proportion of AMF sequences that are non-Glomeraceae is the percentage of AMF sequences produced that are not members of the Glomeraceae family. The proportion of plants exclusively colonised by non-Glomeraceae is the percentage of Glomeromycotina colonised plant samples that only contained non-Glomeraceae AMF. The data required to calculate this value was not available in all studies. An asterisk indicates that the data required was not published. In those surveys where Restriction Fragment Length Polymorphism (RFLP) was used for identification of clones instead of DNA sequencing the values are in italics. In some studies, samples/data were pooled (†) so the result of 0% of AM samples being exclusively colonised by non-Glomeraceae fungi may not be accurate. For one study only data for newly discovered AMF taxa was available (‡). References are as follows: 1 - Desirò *et al.* Proc Biol Sci 280, 20130207 (2013); 2 - Rimington *et al.* New Phytol 205, 1394-1398 (2015); 3 - Davison *et al.* Science 349, 970-973 (2015); 4 - Öpik *et al.* Mycorrhiza 23, 411-430 (2013); 5 - Davison *et al.* FEMS Microbiol Ecol 78, 103-115 (2011); 6 - Öpik *et al.* New Phytol 184, 424-437 (2009); 7 - Öpik *et al.* New Phytol 179, 867-876 (2008); 8 - Heinemeyer *et al.* Glob Chang Biol 10, 52-64 (2003); 9 - Daniell *et al.* FEMS Microbiol Ecol 36, 203-209 (2001); 10 - Husband *et al.* Mol Ecol 11, 2669-2678 (2002); 11 - Douhan *et al.* Mycorrhiza 15, 365-372 (2005); 12 - Scheublin *et al.* Appl Environ Microbiol 70, 6240-6246 (2004); 13 - Bidartondo *et al.* Nature 419, 389-392 (2002).

| **Plant** | **Proportion of AMF sequences/clones non-Glomeraceae** | **No. sequences/clones produced (no. non-Glomeraceae)** | **Proportion of AM plants exclusively colonised by non-Glomeraceae AMF** | **No. AMF-positive samples (no. exclusively colonised by non-Glomeraceae)** | **Ecosystem** | **No. plant species** | **Analysis method** | **Reference** |
| --- | --- | --- | --- | --- | --- | --- | --- | --- |
| **Early-diverging plants** |  |  |  |  |  |  |  |  |
| Liverworts | 40% | 326 (131) | 36% | 255 (93) | Various (global survey) | ≥85 | Molecular cloning | This study |
| Hornworts | 56% | 195 (109) | 52% | 86 (45) | Various (global survey) | ≥24 | Molecular cloning | (1) |
| Lycopods | 0% | 6 (0) | 0% | 4 (0) | Various (global survey) | 20 | Molecular cloning | (2) |
| Ferns | 21% | 28 (6) | 13% | 32 (4) | Various (global survey) | 19 | Molecular cloning | (2) |
|  |  |  |  |  |  |  |  |  |
| **Flowering plants** |  |  |  |  |  |  |  |  |
| Various flowering plants | 6% | 912,515 (58,927) | 0.2% | 836 (2) | Various (global survey) | ≥153 | 454 sequencing | (3) |
| Various flowering plants | 2%‡ | 6011 (128) | 0%/- | 257 (*) | Various (global survey) | 96 | 454 sequencing and molecular cloning | (4) |
| Herbaceous plants | 7% | 2,486 (164) | 0%/- | 212 (*) | Boreal forest | 11 | Molecular cloning | (5) |
| Herbaceous plants | 1% | 126,655 (982) | 0%/- | 489 (0†) | Boreal forest | 10 | 454 sequencing | (6) |
| Herbaceous plants | 4% | 911 (37) | 0%/- | 90 (0†) | Boreal forest | 5 | Molecular cloning | (7) |
| Grassland plants | *4%* | *510 (21)* | - | * | Grassland (experimental setup) | 6 | Molecular cloning and RFLP | (8) |
| Arable crops | *4%* | *303 (12)* | - | 79 (*) | Arable fields | 4 | Molecular cloning and RFLP | (9) |
| Tree seedlings | *5%* | *1536 (71)* | 0%/- | 9 (0†) | Tropical forest | 3 | Molecular cloning and RFLP | (10) |
| Woodland plants | 5% | 57 (3) | 0% | 16 (0) | Oak woodland | 3 | Molecular cloning | (11) |
| Legumes | *4%* | * | - | 24 (*) | Grassland | 3 | Molecular cloning and RFLP | (12) |
| Myco-heterotrophs | 2% | 102 (2) | 0% | 18 (0) | Rain forest | 7 | Molecular cloning | (13) |

**Table S6. Occurrence and size of fungal structures in Glomeromycotina-liverwort symbiosis.** Values estimated from electron micrographs. Values separated by dashes indicate the range and average (bold) of measurements.

|  | **Vesicles (µm)** | **Major hyphae (µm)** | **Arbuscules (µm)** | **Coils + other features (µm)** |
| --- | --- | --- | --- | --- |
| **Pelliidae** |  |  |  |  |
| *Allisonia cockaynei* | 23 | 1.9-**4.3-**5.5 | 0.8-**1.1**-1.3 |  |
| *Calycularia crispula* |  | 2.9-**3.1-**3.7 |  |  |
| *Fossombronia husnotii* | 14 | 3.1-**4.1**-4.8 | 0.9-**1.2**-1.4 |  |
| *F. foveolata* (North Wales) |  | 4.4-**5.4**-5.8 | 1.0-**1.2**-1.4 | Coils 1.6-**1.8**-2.4  Swellings 3.6-**4.0**-5.4 |
| *F. foveolata* (South East England) | 14,21,30 | 3.2-**3.9-**4.7 | 0.8-**1.3**-1.5 | Coils 1.4-**1.9**-2.5  Cauliflory 2.0-**3.3**-4.7  Swellings 3.6-**4.2**-5.0 |
| *Pallavicinia xiphoides* |  | 2.9-**3.5**-4.4 | 0.9-**1.3**-1.5 | Coils 1.9-**2.3**-3.3 |
| *Pellia epiphylla* | 45 | 4.6-**4.8-**5.1 | 0.9-**1.0**-1.1 | Coils 2.8-**2.9**-3.8 |
| *Sewardiella tuberifera* | 8 | 2.3-**2.8**-3.2 | 1.3-**1.5**-1.7 |  |
| *Symphogyna brasiliensis* |  | 3.2-**3.7-**5.5 | 0.7-**0.8**-1.0 |  |
|  |  |  |  |  |
| **Marchantiopsida** |  |  |  |  |
| *Asterella australis* |  | 2.1-**2.7**-3.4 | 1.2-**1.4**-1.7 | Coils 1.6-**2.2**-2.4 |
| *Conocephalum conicum* |  | 3.6-**4.3-**6.3 | 0.8-**0.9**-1.5 | Coils 1.8-**2.1**-2.3 |
| *Dumortiera hirsuta* | 16 | 2.8-**3.3**-3.8 | 0.7-**0.9**-1.4 | Coils 1.5-**2.0**-2.3 |
| *Lunularia cruciata* |  | 2.4**-4.4**-6.5 | 0.8-**1.2**-1.5 |  |
| *Marchantia foliacea* | 25 | 2.7-**3.2**-3.8 | 0.5-**0.7**-1.1 | Coils 1.4-**1.8**-2.2 |
| *M. paleacea* |  | 3.6-**5.0**-7.0 | 0.5-**0.7**-1.1 | Coils 2.1-**2.5**-3.2 |
| *M. pappeana* | 25 | 2.1-**3.6**-5.5 | 0.9-**1.2**-1.4 | Coils 1.9-**2.4**-2.8 |
| *Monoclea forsteri* |  | 1.5-**2.9-**3.6 | 1.0-**1.2**-1.5 |  |
| *Neohodgsonia mirabilis* | >30 | 2.2-**3.2**-5.1 | 0.5-**0.8**-1.8 | Coils 1.2-**1.8**-2.8 |
| *Plagiochasma rupestre* | 31 | 3.1-**3.3**-3.8 | 0.1-**1.0-**1.8 |  |
| *Preissia (Marchantia) quadrata* | 18.5 | 4.7-**5.6**-6.3 | 0.9-**1.3**-1.6 |  |
