# Supplementary material for: Ancient plants with ancient fungi: liverworts associate with early-diverging arbuscular mycorrhizal fungi
Source: Proc Biol Sci. 2018 Oct 10;285(1888):20181600. doi: 10.1098/rspb.2018.1600 (PMC6191707; doi:10.1098/rspb.2018.1600)
Supplement: Habitats: Ecological and morphological considerations [file rspb20181600supp3.docx]

**Habitats: Ecological and morphological considerations**

Full details of the ecology and morphology of Glomeromycotina-containing thalloid liverworts can be found in regional floras, in particular Paton (1999) and Blockeel *et al.* (2014) for the British Isles and Schuster (1992a,b) for North America. This published information, together with our own observations when collecting and dissecting all the specimens used in the present study, reveal some general features of these associations. Liverworts colonised by Glomeromycotina fungi almost always grow directly on mineral soils with rhizoids and/or axes penetrating the substrata. They are absent from the saxicolous family, the Cyathodiaceae, from *Phyllothallia* which lacks rhizoids and grows over other bryophytes, the submerged aquatic *Monoselenium* and from collections of *Conocephalum* growing in very wet habitats. Only a handful of Glomeromycotina-containing thalloid liverworts are regarded as strict calcicoles/basicoles (eg *Preissia quadrata, Reboulia hemispherica, Plagiochasma rupestre, Targionia hypophylla, Pellia endiviifolia*). With the striking exception of many members of the Fossombroniaceae most of the fungus-containing taxa are long-lived perennials.

Considering the large number of species covered by the present global survey, there are very few instances where we found different Glomeromycotina-containing liverworts growing together and thus where liverwort-liverwort host swapping might be possible. The closest fungal matches are between *Pellia epiphylla, Dumortiera* and *Conocephalum* which often grow together. Similarly, Glomeromycotina-containing liverworts are rarely found next to liverworts with ascomycete and basidiomycete symbionts. *Aneura pinguis* is the most notable exception. Undoubtedly, the biggest ecological symbiotic paradox in terms of shared highly seasonal short-lived life cycles is the numerous *Fossombronia* species growing together with the always fungus-free Sphaerocarpaceae, Ricciaceae and Oxymitraceae.

Turning to thallus anatomy, Glomeromycotina fungi are invariably restricted to fleshy parenchymatous midrib regions and are never found on thin lamellar extensions (Fig. S7-S10). The paucity of such tissues could well account for the absence of symbionts in Cyathodiaceae, Sphaerocarpaceae and Riellaceae, but not Ricciaceae, Oxymitraceae or Exomothecaceae. It is also noteworthy that the fungus-free *Moerckia flotoviana* and *M. hibernica* both lack the fleshy fungus-containing midrib found in *M. blyttii* and *Pallavicinia* species lacking fungi (eg *P. lyellii*) lack the underground rhizomes that harbour the endophytes in other members of the family.

**References**

**Blockeel TL, Bosanquet SDS, Hill MO, Preston CD. 2014**. *Atlas of British and Irish bryophytes.* *Vol.1.* Newbury: Pisces Publications.

**Paton JA. 1999.** *The liverwort flora of the British Isles.* Colchester: Harley.

**Schuster RM. 1992a.** *The Hepaticae and Anthocerotae of North America, east of the hundredth meridian. Vol. V.* Chicago: Field Museum of Natural History.

**Schuster RM. 1992b.** *The Hepaticae and Anthocerotae of North America, east of the hundredth meridian. Vol. VI.* Chicago: Field Museum of Natural History.
